# The traffic concentration effects of urban navigation services

**DOI:** 10.1038/s41467-026-75254-8

**Published:** 2026-07-11

**Authors:** Giuliano Cornacchia, Mirco Nanni, Dino Pedreschi, Luca Pappalardo

**Affiliations:** 1 Institute of Information Science and Technologies (ISTI), National Research Council (CNR), Pisa, Italy; 2 Department of Computer Science, University of Pisa, Pisa, Italy; 3 Scuola Normale Superiore, Pisa, Italy

**Keywords:** Geography, Computer science

## Abstract

The collective impact of navigation services remains unclear: while often beneficial to individual drivers, they can unintentionally reshape urban traffic patterns. We simulate their impact in Florence, Milan, and Rome (Italy), integrating GPS data, road networks, and route recommendations from leading providers. We identify a concentration effect: as adoption increases, route diversity declines, and traffic and emissions converge onto fewer roads. At full adoption, route diversity decreases by up to 14% compared to a baseline where recommendations are ignored. Moreover, navigation services reduce CO_2_ emissions at low adoption levels, but these benefits diminish, disappear, or even reverse beyond a city- and service-specific threshold. We replicate our experiments in an abstract setting, obtaining results consistent with those observed in real-world cities.

## Abstract (web summary)

Navigation services concentrate traffic onto a few roads as adoption increases. Simulations in Florence, Milan, and Rome show route diversity drops by up to 14% at full adoption, and CO_2_ benefits vanish or reverse beyond a city- and service-specific threshold.

## Introduction

Navigation services (e.g., Google Maps and TomTom) recommend routes based on traffic conditions, guiding the movements of millions of drivers worldwide. While beneficial to individual drivers, algorithmic navigation has been linked to numerous documented cases of localised congestion^1–4^. Yet, the trade-off between their benefits and detrimental effects on traffic dynamics remains poorly understood^5,6^.

Research in this domain yields fragmented and contradictory results, primarily focused on specific navigation services and urban contexts^5–8^. A simulation conducted by Google Maps in Salt Lake City reported measurable benefits from the use of its routing system, including an average reduction of 6.5% in travel time and a 1.7% decrease in CO_2_ emissions^9^. Other studies find that some routing strategies can effectively mitigate CO_2_ and NO_*x*_ emissions^10–13^, reduce energy consumption^10,11^, vehicle miles travelled^14^, and accident risks^15^. Nevertheless, research also highlights unintended drawbacks, such as increased travel times, heightened exposure of populations to NO_*x*_, elevated traffic volumes in certain areas^12,13^, and a redistribution of traffic from highways to local streets, parks, and tourist destinations^14,15^. These studies are difficult to generalise, replicate, and compare because they rely on specific data sources and methodologies. Overall, there is a lack of a robust, unified framework to assess the multifaceted impact of algorithmic navigation across multiple cities. This study quantifies the impact of various navigation services on road network usage and CO_2_ emissions in three cities of different sizes.

We design a simulation framework that reproduces urban traffic dynamics by integrating mobility demand information from GPS data, road networks from OpenStreetMap, and route recommendations from several popular navigation services: Bing Maps, Google Maps, Mapbox, and TomTom. We use this framework to assess the impact of these navigation services in Florence, Milan, and Rome (Italy) by simulating different levels of service adoption. For each adoption rate, vehicles are randomly assigned to treatment and control groups, with the treatment group following algorithmic route recommendations. This setup enables a causal assessment of how adoption rates of navigation services determine changes in traffic dynamics.

We find that route recommendations substantially reshape road network usage and CO_2_ emissions. Across all cities and navigation services, higher adoption rates cause a reduction in route diversity, i.e., recommended routes increasingly concentrate on a smaller set of roads. Under low traffic load, navigation services reduce CO_2_ emissions. Under high traffic load, navigation services reduce emissions at low adoption levels; however, these benefits weaken, vanish, or reverse as adoption increases. To assess the generalisability of these findings, we replicate our experiments using an abstract representation of road networks and traffic dynamics, finding solid agreement with the patterns observed in real-world cities.

Our study sheds light on the multifaceted impact of algorithmic navigation across multiple cities and services, and it is replicable worldwide, given the widespread availability of mobility demand and road network data. Ultimately, our framework supports urban decision-makers in assessing the impacts of navigation services and designing targeted policy interventions.

## Results

### Simulation framework

Our simulation framework leverages SUMO^16–18^, an open-source, agent-based traffic simulator developed by the German Aerospace Center and used in several large-scale studies for its realism in replicating traffic dynamics^8,9,19–21^. SUMO simulations capture the behaviour of a set of vehicles moving and interacting on a road network, including dynamics at road intersections, queues at traffic lights, and slowdowns due to traffic congestion.

A city’s road network is represented as a directed multigraph, where nodes correspond to road intersections and edges to road segments. We extract road networks from OpenStreetMap (see “Methods” for details). Urban mobility demand is modelled through an Origin-Destination (OD) matrix, which captures the volume of trips between different areas of the city. The OD matrix can be inferred from various data sources, including travel surveys, GPS data, and mobile phone data^22–25^.

We partition a city’s area into equally sized square tiles of 1 km^2^ and estimate OD flows between these tiles using GPS data describing trips of private vehicles (see “Methods”). Square tiles provide a spatially uniform discretisation, avoiding biases introduced by administrative boundaries (e.g., census zones or districts), which may vary in size, shape, and functional meaning. This choice ensures consistent spatial resolution across the city and facilitates comparability across urban areas. The inferred OD matrix is then translated into a mobility demand, i.e., a set of individual vehicle trips that serve as inputs to the traffic simulation. See Supplementary Note 1 for further details on GPS data and the computation of the mobility demand.

We assign routes to trips in the mobility demand using the public APIs provided by several navigation services. These services recommend a route between an origin edge and a destination edge considering various factors, such as the typical traffic conditions at the time when the trip starts. API requests allow us to simulate drivers’ common queries, such as “Take me from location A to location B by car at 8:30 a.m.” Although the algorithms underlying navigation services are opaque to us, we assume that they provide route recommendations optimised for individual requests rather than favouring system-level coordination. This is justified by their inability to enforce or forecast driver compliance, which makes system-optimal solutions based on Wardrop’s second principle^26,27^ unreachable.

We query routes for future dates relative to the request time, thereby forcing the APIs to rely on typical traffic information rather than real-time conditions. This choice avoids incorporating signals shaped by exogenous and unpredictable events—such as accidents, strikes, or roadworks—that cannot be modelled within the simulation.

**Fig. 1:**
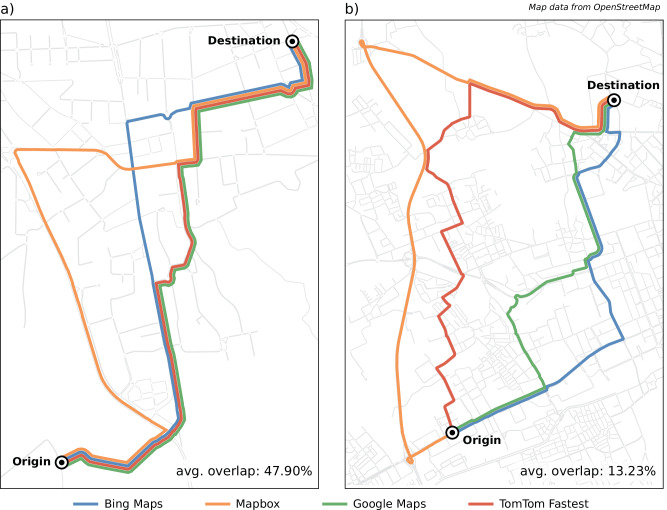
Routes recommended by navigation services. **a**, **b** Two Origin-Destination pairs in Milan and the routes suggested by Bing Maps, Google Maps, Mapbox, and TomTom Fastest. **a** An illustrative example where recommendations largely overlap (average overlap = 47.90%); **b** a case where recommendations largely diverge (average overlap = 13.23%). The difference among navigation services occurs because they rely on different criteria and diverse historical data on traffic conditions. Road network data from OpenStreetMap (ⓒ OpenStreetMap contributors), available under the Open Database License (ODbL; https://www.openstreetmap.org/copyright). OpenStreetMap documentation is published under CC BY-SA 2.0 (https://creativecommons.org/licenses/by-sa/2.0/).

We consider a set of navigation services that provide public APIs: Bing Maps, Google Maps, Mapbox, TomTom Fastest, TomTom Shortest, and TomTom Eco-routing. At the time of the experiments, Apple Maps, Waze, and Google Maps eco-routing could not be included due to the unavailability of public APIs. These services were selected because of their commercial relevance and widespread adoption, which makes them suitable for large-scale, reproducible experiments (see Supplementary Note 2 and Table 1 for details). Different services may recommend different routes for the same trip (Fig. 1a, b), reflecting differences in their optimisation objectives, heuristics, and underlying historical traffic data. We find that the average route overlap across services ranges from 68% to 97% with an average of 76.4% (see Supplementary Note 2). We account for this variability by comparing results across multiple navigation services rather than relying on a single one. We refer to a trip that follows a route suggested by a navigation service *s* as an *s*-routed trip.

**Table 1:** Characteristics of selected navigation services

	provider	profile name	profile description
Bing Maps	Bing Maps	TimeWithTraffic	Optimisation of travel time using current traffic information.
Google Maps	Google Maps	Not available	Not available
Mapbox	Mapbox	Driving-traffic	Lowest probability of slowdowns given current and historical traffic conditions.
TomTom Eco-routing	TomTom	Eco	Trade-off between travel time and fuel consumption.
TomTom Fastest	TomTom	Fastest	Shortest travel time while keeping the routes sensible.
TomTom Shortest	TomTom	Short	Trade-off between travel time and travel distance.

The table shows the service provider, the name of the profile used (i.e., the routing criteria), and a description of the criteria.

To assess the impact of a navigation service *s* on traffic in a given city, we conduct controlled experiments by varying the adoption rate of *s* from *r* = 0% (no adoption) to *r* = 100% (full adoption) in steps of 10%. We assign *r*% of vehicles to a treatment group and the remaining vehicles to a control group. Vehicles in the treatment group are *s*-routed, while those in the control group choose a route autonomously based on approximate knowledge of the road network and traffic patterns. To emulate this approximate knowledge, control vehicles follow a modified version of the fastest route computed by SUMO. This modification introduces a small random deviation from the fastest route to account for imperfections in human route choice, due to limited network knowledge, bounded rationality, and cognitive biases documented in the transportation and behavioural literature^28–30^. The full implementation details of this modification are described in “Methods”. The similarity between SUMO’s fastest routes and those suggested by navigation services lies in the 52–63% range, with an average of 57.2%. This overlap is substantially lower than the similarity observed among navigation services themselves (68–97%), indicating that SUMO relies on different routing criteria and cost functions.

For statistical robustness, we repeat the simulation for a given adoption rate *r* ten times, using different random compositions of the treatment and control groups in each run. Furthermore, we repeat all the above steps considering both low and high traffic loads. Low traffic load refers to situations with few circulating vehicles in the city, such as off-peak hours and nighttime. High traffic load indicates that traffic is approaching congestion. See Supplementary Note 3 for details on the definition of low and high traffic loads.

We evaluate the impact of navigation services both in isolation, where all the vehicles in the simulation use the same service, and in combination, where vehicles may choose among different navigation services. We present only the isolation scenario since the results are analogous (see Supplementary Note 2 for results on the combination scenarios). In our analysis, we focus on two measures: route diversity and CO_2_ emissions. Route diversity captures the spatial concentration of routes across the road network, and it is defined as the number of edges traversed at least once by any vehicle. CO_2_ emissions provide an aggregate, environmentally relevant outcome of traffic dynamics, integrating the effects of congestion, speed fluctuations, and acceleration patterns. We compute this measure using the microscopic emission model implemented in SUMO^18^, which estimates instantaneous vehicle emissions as a function of speed and acceleration. See “Methods” for details on the implementation of the two metrics.

A limitation of our experimental setup arises from interaction effects: since vehicles move on a shared road network, those assigned to the control group inevitably interact with vehicles in the treatment group^31,32^. As a result, the outcomes of individual vehicles are not fully independent and observable differences between groups are expected to be underestimated. This is a common situation when performing controlled experiments in complex social systems^33^. Our results should therefore be interpreted as conservative estimates of the impact induced by navigation services.

### Impact of navigation services

We conduct experiments in three cities in Italy: Florence, Milan, and Rome. These cities were chosen due to their heterogeneity in size, population, and road network structure (see Supplementary Fig. S1). For instance, Rome has an extensive and sparse road network with relatively long edges and moderately winding roads. Milan is slightly smaller and denser than Rome and is characterised by shorter edges and a more regular network structure. Florence, the smallest, has the most winding and spatially intricate network. The difference among cities enables the assessment of the robustness of algorithmic navigation effects across different urban morphologies. We simulate traffic in each city during the morning peak, defined from vehicular GPS data as the 1 h morning slot with the highest traffic intensity.

**Fig. 2:**
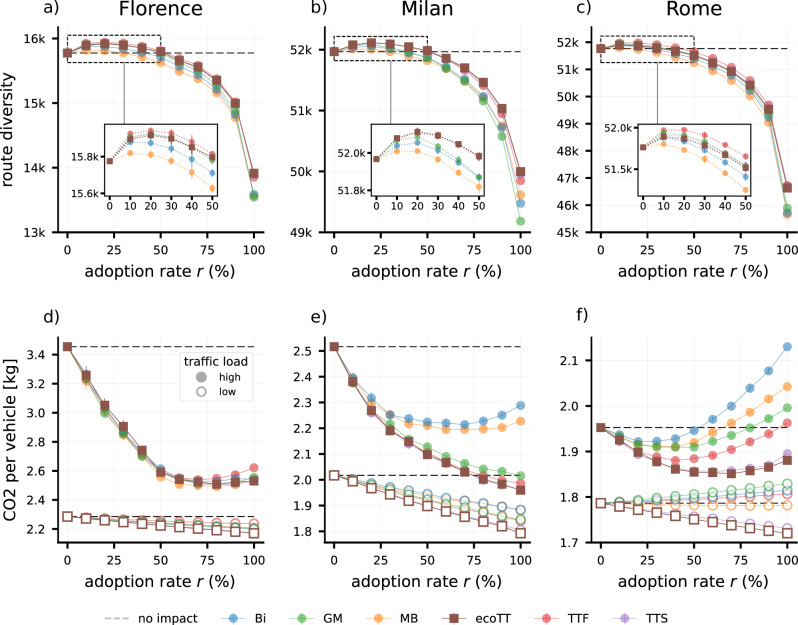
Impact of navigation services on route diversity and CO_2_ emissions. **a**–**c** Service adoption rate, *r*, versus route diversity at high traffic load in Florence, Milan, and Rome (Italy). The inset plots zoom in on the range *r* = 0%, …, 50%, to highlight the slight initial increase of route diversity. Increased adoption of navigation services causes a reduction in route diversity, with only minor fluctuations among simulation runs, cities, and navigation services. **d**–**f** Service adoption rate versus average CO_2_ emissions per vehicle at high traffic load (filled markers) and low traffic load (empty markers). At high traffic load, when *r* is low, CO_2_ emissions decrease considerably; when *r* exceeds a certain city- and service-dependent threshold, the benefits plateau and, in some cases, even reverse. In all **a**–**f**, the dashed line represents the *r* = 0% scenario; markers indicate the average route diversity or the average CO_2_ emissions over ten simulations with different random choices of *s*-routed vehicles. Square markers refer to the navigation service ecoTT, which employs eco-routing. Vertical bars, indicating the standard deviation across the ten simulations, are hardly visible because they are negligible. Navigation services are indicated as follows: Bi (Bing Maps), GM (Google Maps), MB (Mapbox), TTF (TomTom Fastest), TTS (TomTom Shortest), and ecoTT (TomTom Eco-routing).

Figure  2a–c illustrates the impact of varying the adoption rate *r* on route diversity in the three cities, considering high traffic load. Results under low traffic load are similar (see Supplementary Fig. S2). If the navigation service had a negligible impact, we would expect a constant route diversity as *r* increases (horizontal dashed line in Fig. 2a–c). Instead, route diversity varies with *r* following an exponential function (see Supplementary Note 4). When *r* is below a specific city- and service-dependent threshold (varying from about 25% to 50%), route diversity slightly increases by 0.15–1.05% in Florence, 0.08–0.34% in Milan, and 0.01–0.41% in Rome (see inset plots in Fig. 2a–c) compared to *r* = 0%. On the other hand, when *r* exceeds this threshold, route diversity considerably decreases. At the full adoption rate (*r* = 100%), route diversity is reduced by 11.80–14.34% in Florence, 3.79–6.87% in Milan, and 9.73–14.26% in Rome compared to *r* = 0%. We also examine the distribution of route diversity separately for vehicles in the two groups: vehicles in the treatment group exhibit lower route diversity compared to those in the control group, regardless of the adoption rate (see Supplementary Note 4). Our results have only minor fluctuations among different random compositions of the treatment and control groups and across cities and navigation services. Therefore, changes in route diversity can be confidently attributed to the adoption of algorithmic navigation.

Figure  2d–f shows the average CO_2_ emissions per vehicle produced at different traffic loads and adoption rates. When the traffic load is low, navigation services are generally beneficial: in most cases, CO_2_ emissions are lower than in the *r* = 0% scenario, showing a near-linear decreasing trend with *r*. At the full adoption rate, navigation services reduce CO_2_ emissions by 2.15–5% in Florence and 6.64–11.13% in Milan. In Rome, some navigation services reduce CO_2_ emissions by 0.26–3.69%, while Google Maps, TomTom Fastest, and Bing Maps slightly increase them by 2.4%, 1.13%, and 1.57%, respectively. See Supplementary Table S2 for a detailed comparison across cities and navigation services.

**Fig. 3:**
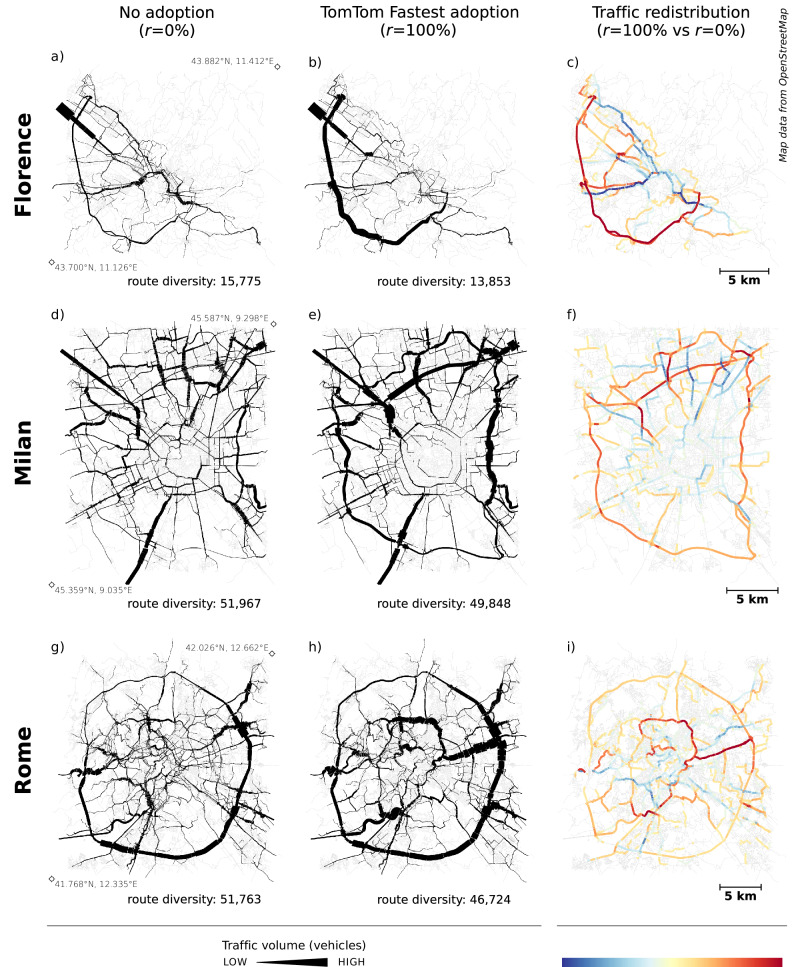
Redistribution of routes. Rows correspond to cities: Florence (**a**–**c**), Milan (**d**–**f**), and Rome (**g**–**i**). Columns represent navigation scenarios under TomTom Fastest (TTF): no navigation adoption (**a**, **d**, **g**), full adoption (**b**, **e**, **h**), and traffic redistribution (**c**, **f**, **i**). For the no-navigation and full-navigation scenarios, road edge widths are proportional to traffic volume. The redistribution scenario shows differences in road usage between the full-adoption and no-adoption scenarios: blue edges denote road segments where the full adoption of TTF reduces traffic relative to the no-adoption scenario; red edges indicate segments where traffic increases. Colour intensity is proportional to the magnitude of the traffic change. Across all cities, full TTF adoption concentrates traffic onto a smaller subset of road segments, with traffic increasingly redistributed onto high-capacity roads. Road network data from OpenStreetMap (ⓒ OpenStreetMap contributors), available under the Open Database License (ODbL; https://www.openstreetmap.org/copyright). OpenStreetMap documentation is published under CC BY-SA 2.0 (https://creativecommons.org/licenses/by-sa/2.0/).

However, at high traffic load, the impact of navigation services depends on the adoption rate. When *r* is low, CO_2_ emissions decrease; when *r* exceeds a certain city- and service-dependent threshold, the benefits plateau and in some cases CO_2_ emissions increase (see Fig. 2d–f). At full adoption (*r* = 100%), reduced route diversity leads to a concentration of CO_2_ emissions on fewer roads, thereby increasing inequality in their spatial distribution (see Supplementary Note 5). Fig. 3 illustrates this effect for TomTom Fastest: at *r* = 0% (Fig. 3a, d, g), the traffic is more evenly distributed, whereas at *r* = 100% (Fig. 3b, e, h), there is a concentration of traffic and CO_2_ emissions on fewer edges. This effect is further illustrated by the Lorenz curves and corresponding Gini coefficients of CO_2_ emissions across road segments (Supplementary Fig. S10), reported for adoption rates *r* = 0%, *r* = 50%, and *r* = 100%. In Florence and Milan, emissions are more evenly distributed at *r* = 50% than at either 0% or 100%, as indicated by a lower Gini coefficient, reflecting a temporary increase in route diversity before route convergence takes hold.

At *r* = 100%, emissions become more concentrated: the Gini coefficient increases by 0.023 in Florence, 0.025 in Milan, and 0.031 in Rome compared to 50% adoption. This corresponds to an additional 2.04% (Florence), 2.31% (Milan), and 3.9% (Rome) of total CO_2_ emissions being concentrated on the top 20% most polluted edges.

Across all cities, we observe a consistent shift of routes, and consequently CO_2_ emissions, towards high-capacity road infrastructures such as highways and major arterial corridors (see Fig. 3c, f, i). These infrastructures are typically favoured by navigation services because they offer higher speed limits and accommodate large traffic volumes. However, despite accounting for only about 6% of the road network, these high-capacity road infrastructures absorb a large share of CO_2_ emissions. This imbalance becomes even more pronounced as navigation service adoption increases. From 0% to 100% adoption, the share of emissions on high-capacity infrastructures rises from 17.61% to 36.25% in Florence, 9.94% to 26.42% in Milan, and 26.02% to 33.66% in Rome. The full breakdown of CO_2_ emissions by road type is provided in Supplementary Note 5.

Results further indicate city-specific variations in the observed effects. In Florence, the largest reduction in average CO_2_ emissions occurs at *r* = 70–80%; beyond this point, the benefits diminish (see Fig. 2d). In Milan, Google Maps and TomTom-based services reduce CO_2_ emissions as *r* increases, achieving reductions of 19–22% at the full adoption rate. In contrast, Mapbox and Bing Maps reach their highest reductions (≈12%) when *r* = 60% and *r* = 70%, respectively; beyond these rates, the benefits diminish (CO_2_ reductions decrease to 9% relative to *r* = 0%). In Rome, optimal adoption rates vary by service: 20% for Bing Maps, 40% for Mapbox, Google Maps, and TomTom Fastest, 60% for TomTom Shortest, and 70% for TomTom Eco-routing, leading to an average CO_2_ reduction of 3.29%. Notably, Bing Maps, Mapbox, Google Maps, and TomTom Fastest lead to increased CO_2_ emissions beyond certain thresholds compared to *r* = 0%: 9.10% for Bing Maps, 4.61% for Mapbox, 2.22% for Google Maps, and 0.52% for TomTom Fastest (see Supplementary Table S2 for details on the CO_2_ reductions). In general, we observe an adoption range for most navigation services in the three cities—between 60% and 80%—beyond which the environmental benefits of algorithmic navigation vanish.

A comparison of Florence, Milan, and Rome reveals both common and city-specific patterns: route concentration is a consistent outcome of increased service adoption, while CO_2_ emissions vary with the spatial redistribution of traffic induced by navigation services. In Florence, navigation services shift traffic towards an arterial road that is moderately used in the no-adoption scenario (Fig. 3a, b). This redistribution is associated with a substantial reduction in CO_2_ emissions that stabilises as this corridor becomes saturated at high adoption levels. In Rome, traffic is redirected onto a few major roads that are already heavily used in the baseline scenario (Fig. 3g, h). This pattern may explain the pronounced increase in CO_2_ emissions at high adoption rates. Milan lies between these two cases, navigation services concentrate traffic onto several major roads that are lightly trafficked in the baseline scenario (Fig. 3d, e), which is consistent with the moderate decreases in CO_2_ emissions observed at high adoption rates.

Our results show that even an eco-routing service (TomTom Eco-routing) exhibits CO_2_ trends comparable to those of other navigation services (see brown squares in Fig. 2). This indicates that optimising routes at the individual level with explicit environmental objectives does not necessarily translate into reduced emissions at the system level. Environmental sustainability depends not only on the properties of individual routes, but also on the collective distribution of routing decisions across the network.

To further investigate the relationship between route diversity and CO_2_ emissions under high traffic load, we examine the correlation between the marginal changes in route diversity (Δ*D*_*r*_) and those in CO_2_ emissions (Δ*E*_*r*_). Δ*D*_*r*_ is defined as the difference in route diversity between adoption rates *r* − 10% and *r*, and Δ*E*_*r*_ as the difference in total CO_2_ emissions over the same interval. Consequently, positive values of Δ*D*_*r*_ and Δ*E*_*r*_ indicate reductions in route diversity and CO_2_ emissions, respectively.

**Fig. 4:**
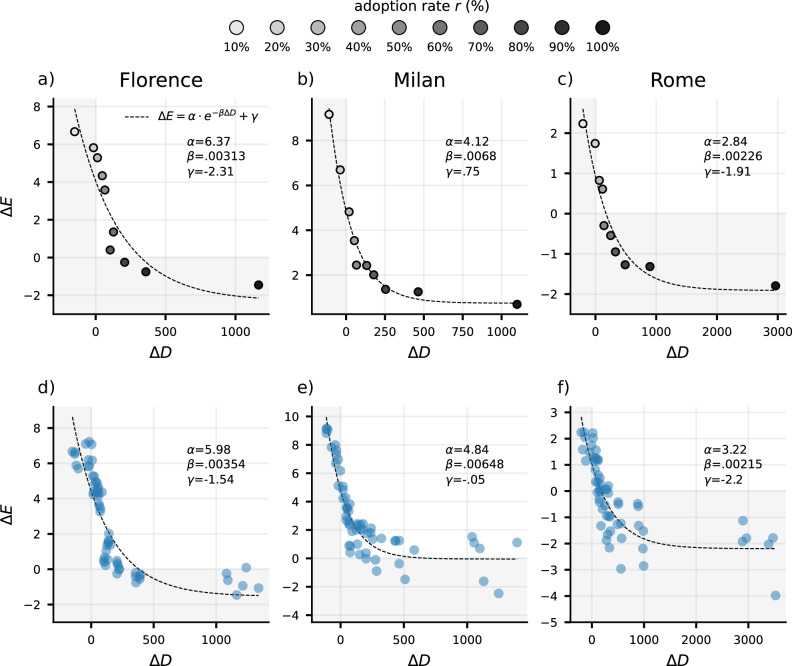
Relationship between route diversity and CO_2_ emissions. **a**–**c** The relationship between the marginal change in route diversity (Δ*D*) and the marginal change in CO_2_ emissions (Δ*E*) for TomTom Fastest (TTF) in Florence, Milan, and Rome (Italy). We find similar results for the other navigation services. The points are shaded from light grey to black, representing the service adoption rate (from *r* = 10% to *r* = 100%). **d**–**f** Same relationship for all navigation services. Regions where Δ*D* or Δ*E* are negative are highlighted in grey. The black dashed line represents the exponential decay fit for each scenario. At low adoption rates, slight increases in route diversity lead to substantial reductions in CO_2_ emissions. As *r* increases, small reductions in route diversity result in moderate CO_2_ reductions. However, as Δ*D* further increases, Δ*E* decreases, indicating a diminishing return effect. This pattern is consistent across all cities and navigation services.

Across all cities, we observe a strong negative association between Δ*D*_*r*_ and Δ*E*_*r*_: Spearman’s rank correlation coefficient is *ρ* = −0.958 in Florence, *ρ* = −0.882 in Milan, and *ρ* = −0.899 in Rome. This association is well described by an exponential decay function (see Fig. 4a–c). At low adoption rates, small increases in route diversity are associated with large reductions in CO_2_ emissions. As the adoption rate increases, small reductions in route diversity result in moderate CO_2_ reductions. Further losses in route diversity are accompanied by little or no additional benefit, indicating a diminishing return effect. This pattern is consistent across all cities and navigation services (Fig. 4d–f).

### Driving factors of concentration patterns

To demonstrate that the observed concentration effects do not depend merely on network layouts and mobility demands of the three cities, we replicate our experiments in an abstract setting. We model the road network as a regular grid in which all roads are bidirectional and have identical speed limits. This grid structure enables precise control of network topology while preserving essential flow interaction properties. As the grid network is synthetic, we cannot rely on navigation service APIs. Instead, we emulate navigation services by assigning vehicles the fastest route, which provides an individually optimised route to the destination. Accordingly, *s*-routed vehicles follow the fastest path, while the remaining vehicles are assigned perturbed alternatives, consistent with our experiments on real-world cities.

**Fig. 5:**
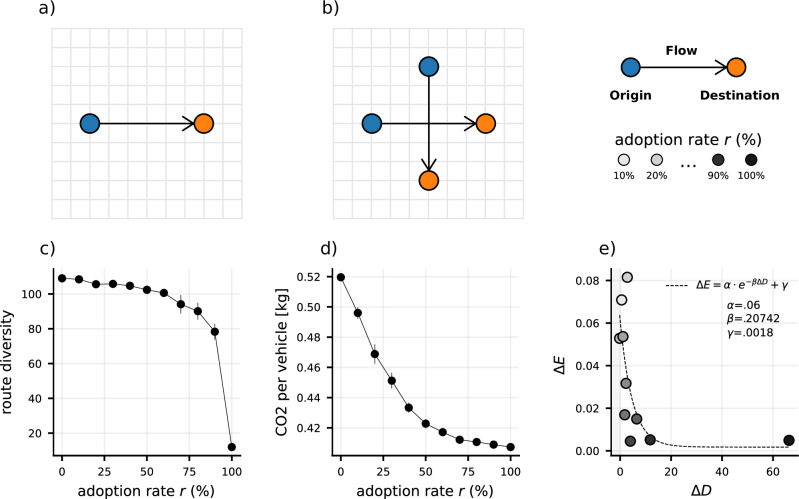
Impact of algorithmic routing in an abstract setting. **a**, **b** A 10 × 10 grid network with synthetic Origin-Destination (OD) flows. **a** Setting with a single OD flow. **b** Setting with two intersecting OD flows that increase vehicle interactions at the grid centre. Routed vehicles follow the fastest route, while non-routed vehicles take perturbed variants. **c** Route diversity as a function of adoption rate *r*. With two OD flows, route diversity decreases rapidly as *r* increases, replicating patterns observed in real-world cities. **d** Average CO_2_ emissions per vehicle versus adoption rate. Emissions initially decline, then plateau due to increased congestion at shared intersections. **e** Marginal relationship between changes in route diversity (Δ*D*) and CO_2_ emissions (Δ*E*), showing a nonlinear trend consistent with real-world cities. Points are shaded from light grey to black to represent increasing adoption rates from *r* = 10% to *r* = 100%.

We first simulate a single OD flow on a 10 × 10 grid network (see Fig. 5a), where all vehicles share the same origin and destination. We vary the adoption rate as in the experiments on real-world cities. We find that, as the adoption rate increases, route diversity declines but CO_2_ emissions continue to decrease (see Supplementary Fig. S13). This suggests that, in the absence of vehicle interactions at intersections, reduced route diversity alone does not necessarily lead to a diminishing return effect.

To enforce vehicle interactions, we simulate two orthogonal OD flows on the same grid (see Fig. 5b). In this configuration, the fastest routes intersect at the centre of the grid, becoming interdependent and producing collective concentration effects. All key patterns observed in real-world cities as the adoption rate increases are reproduced (see Fig. 5c–e): a sharp reduction in route diversity, a plateau in CO_2_ emissions, and a nonlinear relationship between Δ*D*_*r*_ and Δ*E*_*r*_. The simple abstract setting also captures the rise in CO_2_ inequality as the adoption rate approaches 100%, with the increasing Gini coefficient aligning closely with the patterns observed in real-world cities (see Supplementary Fig. S19).

We extend the analysis to a larger grid and randomised grid-like networks and find that the same patterns persist (see Supplementary Note 6 for full details). This confirms that the observed concentration patterns are not artefacts of network layouts.

## Discussion

In all three cities, as well as in the abstract setting, we observe a consistent pattern: increasing the adoption rate of navigation services leads to an increased concentration of routes and CO_2_ emissions. These results are consistent with findings from other domains, where algorithms and AI systems have been shown to reinforce conformist behaviour and existing inequalities (e.g., in the recommendation of urban venues^34,35^ and products^36^ as well as in content generation^37^).

We note that the urban road networks considered in this study are not unconstrained systems. Italian cities are characterised by the presence of regulated traffic zones, such as limited traffic zones and, in the case of Milan, a low-emission zone, which restrict access to specific areas or vehicle types. These regulatory constraints are already incorporated into the routing logic of navigation services and therefore into our simulations. The concentration effect we observe arises from the alignment of route recommendations across drivers and is expected to generalise to other urban contexts, even though the specific roads involved may differ.

The spatial concentration observed in our study emerges as a collective property of algorithmic route recommendations. Based on our results, we argue that this effect stems from the tendency of navigation services to suggest similar routes in response to similar requests. Optimal routes are typically funnelled through the same efficient roads; as a consequence, many optimal routes overlap on a limited set of road edges, producing traffic concentration on such edges. The provision of a small number of alternative routes per request (typically no more than three) can only marginally dilute this concentration and cannot eliminate it. Moreover, the coexistence of multiple navigation services is unlikely to mitigate the effect, as competing platforms rely on comparable optimisation principles and therefore produce aligned routing recommendations. The observed concentration pattern carries important implications for urban inequality, environmental sustainability, urban policy, and the governance of digital platforms.

The first implication concerns urban inequality. At high adoption rates, navigation services tend to funnel traffic onto highways and major arterial roads. This spatial redistribution carries various consequences. Neighbourhoods located near highways and major arterial roads—often situated at the urban periphery—are exposed to higher levels of emissions, pollution, and noise, potentially leading to a decline in quality of life and property values. Consequently, the observed concentration effect may exacerbate existing social disparities, in line with previous studies showing how algorithmic decision-making can amplify inequalities in urban environments^15,38–40^.

A second implication concerns environmental sustainability: the impact of navigation services is non-negligible and depends on both traffic load and adoption rate. While algorithmic navigation tends to reduce emissions under low-traffic conditions and low adoption, its effects become mixed at a higher traffic load, with benefits diminishing, vanishing, or even reversing as adoption increases. This context dependence helps explain the fragmentation and apparent contradictions in the existing literature, which often focuses on specific traffic conditions or adoption levels, and shows that the impact of algorithmic navigation cannot be reduced to a simple dichotomy of beneficial or detrimental effects. Notably, TomTom Eco-routing follows the same qualitative dynamics as other navigation services, highlighting a limitation of individualised optimisation to address the environmental footprint of algorithmic navigation. When many drivers are guided towards similar “eco-optimal” paths, traffic concentration on these paths can offset or even reverse the intended environmental benefits.

Third, our simulation framework offers a valuable tool for urban policymaking, as it can be readily instantiated across cities worldwide. The framework allows simulating traffic outcomes across different adoption levels, including counterfactual scenarios in which navigation services are not used. This counterfactual analysis may help assess whether the impacts of algorithmic navigation align with or undermine existing or prospective municipal policies. For example, by comparing the actual traffic with a baseline scenario in which navigation services are not used, policymakers can identify how algorithmic navigation reshapes urban mobility, e.g., amplifying traffic in sensitive areas such as school and hospital districts. This knowledge can inform targeted requests to navigation service providers aimed at mitigating or compensating for such effects. In this sense, our framework aligns with current regulations—such as the EU’s Digital Services Act^41^—which require very large online platforms to detect and understand the systemic risks they pose to society in terms of fundamental rights, public security, and well-being.

Our study provides a systematic investigation of the impact of algorithmic navigation on urban spaces across multiple cities, navigation services, traffic loads, and adoption rates. Our framework allows the simulation of hypothetical yet realistic scenarios that are difficult, if not impossible, to investigate empirically. Direct on-road experiments face several practical and methodological constraints. First, even if a navigation service provider conducted a controlled empirical study, it would capture only a subset of the urban vehicle fleet, leaving non-user movements and interactions unobserved. Second, these controlled studies are difficult to replicate under identical initial conditions, as traffic dynamics are continuously shaped by unpredictable exogenous factors, including mobility demand fluctuations, car accidents, and roadworks. Third, large-scale experiments would require explicit driver consent and coordinated interventions at the urban level, raising critical logistical, regulatory, and ethical challenges. Although simulations can provide only estimates rather than exact measurements, they currently represent a feasible approach for systematically studying the impact of navigation services at scale.

Our simulation framework can also be extended to study other urban mobility externalities associated with navigation services, including traffic safety (e.g., the risk and spatial distribution of car accidents) and neighbourhood-level segregation effects (e.g., the systematic avoidance of certain areas). Beyond navigation services, our simulation framework can be extended to other mobility services, such as car-sharing and ride-hailing. These services exploit matching and dispatching algorithms that associate drivers with users to serve rides on demand^42^. Recent research shows that these services have both beneficial and detrimental effects on traffic congestion, emissions, public transport usage, and social equity^7,43,44^. We can adapt our framework to simulate the impact of these services on various urban externalities. For instance, driver-user matching can be implemented using standard strategies such as assigning the closest idle vehicle^45^, while rides can be assigned to routes either through conventional routing algorithms or by querying navigation service APIs. As in our experimental setup, the adoption rate of these services can then be systematically varied to quantify their impact on route diversity, traffic distribution, and different forms of emissions.

Finally, our study contributes to the broader discussion on human-AI coevolution^6^. Navigation services operate within a dynamic feedback loop: traffic patterns shape algorithmic recommendations, these recommendations influence individual route choices, and the resulting collective behaviour modifies future traffic conditions, in a potentially never-ending cycle^6,46^. Recent work shows that feedback loop mechanisms can amplify existing biases in human-AI ecosystems, spanning domains from social media and online retail to human mobility^7^. In this context, the interdependence between adoption rates, route diversity, and CO_2_ emissions suggests that the concentration effects observed in our non-dynamic setting may be exacerbated when navigation services and users continuously adapt to one another within a feedback loop. Therefore, our results are probably an underestimation of the real impact, and a natural direction for future research is to investigate how the observed impact of navigation services evolves in a coevolutionary setting.

Looking ahead, the key challenge is to design navigation services that preserve route diversity, improving individual travel without inducing collective concentration. While our framework can be used to evaluate the impacts of alternative routing strategies, developing algorithms that account for the city as a complex system remains an open research question.

## Methods

### Road networks

We model a city’s road network as a directed multigraph, where nodes correspond to road intersections and edges to road segments. We extract the road networks of Florence, Milan, and Rome (Italy) from OpenStreetMap (OSM) using SUMO’s OSM Web Wizard. We manually fine-tune the road networks to correct inaccuracies that could cause deadlocks and other unrealistic behaviours during simulations. This fine-tuning phase includes correcting lane number inaccuracies, addressing road continuity disruptions, and modifying turns to align with real-world conditions^19^. We use Google Maps and Google Street View as benchmarks to ensure the accuracy of these adjustments. Overall, we manually corrected <0.15% of the road edges in the network.

### Impact measures

We measure the impact of navigation services on route diversity and CO_2_ emissions. Route diversity captures the extent to which a set of routes explores the road network. Formally, let *R* be a set of routes, route diversity is defined as RD(*R*) = ∣⋃_*r*∈*R*_{*e* ∈ *r*}∣, where *e* ∈ *r* indicates an edge in route *r*. Higher values of route diversity indicate a broader exploration of the network, whereas lower values reflect convergence onto a limited subset of edges.

Vehicle CO_2_ emissions are computed using the emission modelling framework implemented in SUMO, based on the HBEFA3/PC_G_EU4 model^47^. This model estimates instantaneous emissions at each trajectory point *j* as ${{{\mathcal{E}}}}(j)={c}_{0}+{c}_{1}sa+{c}_{2}s{a}^{2}+{c}_{3}s+{c}_{4}{s}^{2}+{c}_{5}{s}^{3}$, where *s* and *a* are the vehicle’s speed and acceleration at point *j*, respectively, and *c*_0_, …, *c*_5_ are vehicle- and emission-specific parameters extracted from the HBEFA database^48^, corresponding to SUMO’s default vehicle emission class, i.e., a gasoline-powered Euro 4 passenger car.

We find that CO_2_ emissions are strongly correlated with other measures, including fuel consumption, NO_*x*_ emissions, particulate matter, and noise, at both the road-segment and per-vehicle levels (Pearson correlation *r* > 0.994 across all cities and navigation services). We therefore report results for CO_2_ emissions only.

### Modified fastest route

Vehicles in the control group follow a modified version of the fastest route implemented by SUMO’s duarouter algorithm. Duarouter computes vehicle routes based on a parameter *w* ∈ [1, +*∞*), which controls the deviation of the generated route from the fastest route. When *w* = 1, duarouter calculates the standard fastest route. When *w* > 1, it dynamically alters the edge weights (expected travel time) by a random factor uniformly drawn from the interval [1, *w*). This random process ensures that different vehicles may get different routes even if their trip has the same origin and destination. As *w* increases, the extent of randomness in the route calculation also increases, resulting in routes that can diverge substantially from the fastest route. The injection of route variability helps us model the imperfections of human driving behaviour, which often deviates from the fastest route due to personal preferences, lack of complete knowledge of the road network, and irrational behaviours^28,30^. In our experiments, we perform simulations introducing a moderate level of randomness in the control group by varying *w* ∈ {3, 5, 7}. Our results are consistent across different values of *w*. For clarity and conciseness, in the main manuscript we report only the results for *w* = 5; results for *w* = 3 and *w* = 7 are discussed in Supplementary Note 7.

### GPS data

To estimate each city’s mobility demand, we use a GPS dataset provided by OCTO that describes the trajectories of thousands of vehicles for various Italian localities over an entire year. The market penetration of the dataset varies between 2 and 5% of the total registered vehicles. Our analysis focuses on Florence, Milan, and Rome (Italy). We selected these cities due to their diverse sizes, populations, and road network structures. The raw dataset includes 7,102,351 GPS points from 24,640 vehicles in Florence, 143,698,720 GPS points from 106,456 vehicles in Milan, and 26,801,872 GPS points from 30,763 vehicles in Rome. The dataset’s validity and reliability are corroborated by its use in prior studies^49–51^.

We process the GPS dataset to create segmented trajectories that represent semantic journeys as follows: first, we filter out GPS points with speeds exceeding 250 km/h^52^. Second, we use a stop detection algorithm^24,52^ to segment each trajectory into sub-trajectories based on identified stops. A stop is identified when a vehicle remains within a distance of 0.2 km from a trajectory point for at least 20 minutes. Third, we consider only trips that start and end within the predefined area of interest. For trips that start or end outside this area but traverse it, we use the first entry point within the area as the origin and the last exit point as the destination. This method preserves commuter trips originating or ending outside the city. Fourth, we keep all trips with a duration between 5 and 60 minutes that depart during the morning peak hours on each Wednesday. This allows us to model high-traffic scenarios (as peak hours represent periods of intense traffic) and to capture typical weekday traffic, avoiding anomalies associated with weekends. Finally, we exclude outlier holiday weeks from the analysis. After the pre-processing step, the dataset includes 5477 trips from 1184 vehicles for Florence (616 distinct flows); 113,323 trips from 15,997 vehicles for Milan (11,967 distinct flows); and 13,647 trips from 1965 vehicles for Rome (1757 distinct flows). See Supplementary Note 1 for further details on the pre-processing.

## Supplementary information

Supplementary Information

Transparent Peer Review file

## Data Availability

The data generated in this study (i.e., simulation outputs including route diversity, CO_2_ emissions, and other traffic measures, for all cities, navigation services, adoption rates, and traffic loads) are available at the repository https://github.com/GiulianoCornacchia/Urban-Impact-Navigators and permanently archived^53^. The processed road network data used in this study are also available at the same repository. The raw GPS trajectory data used in this study are not publicly available due to commercial restrictions; they were provided by OCTO under a data-sharing agreement and cannot be redistributed. To overcome this limitation, in the repository we have provided code to generate an OD matrix for Milan using a publicly accessible dataset available at https://data.d4science.org/ctlg/ResourceCatalogue/gps_track_milan_italy, as well as a routine to create random OD matrices for scenarios where trajectory data is unavailable. The navigation service route suggestions used in this study are not publicly available due to the terms of use of the respective APIs; we have instead provided code to generate the fastest route as a navigation service prototype, including routines to generate perturbations for non-routed vehicles, available at https://github.com/GiulianoCornacchia/Urban-Impact-Navigators.

## Code availability

The Python code implementing the simulation framework and for replicating the analyses in the study is publicly available at https://github.com/GiulianoCornacchia/Urban-Impact-Navigators and permanently archived at Zenodo^53^: 10.5281/zenodo.20001710.
