# Supplementary material for: The traffic concentration effects of urban navigation services
Source: Nat Commun. 2026 Jul 11;17:8548. doi: 10.1038/s41467-026-75254-8 (PMC13482866; doi:10.1038/s41467-026-75254-8)
Supplement: Supplementary file 1 [file 41467_2026_75254_MOESM1_ESM.pdf]

Supplementary Material

The traffic concentration effects of urban navigation services

Giuliano Cornacchia[1], Mirco Nanni[1], Dino Pedreschi[2], Luca Pappalardo[1,3]

1. ISTI-CNR, Pisa, Italy
2. Department of Computer Science, University of Pisa, Pisa, Italy
3. Scuola Normale Superiore, Pisa, Italy

Contents

1 Supplementary Figures **2**

2 Supplementary Tables **15**

3 Supplementary Notes **16**
Supplementary Note 1: GPS data and Mobility Demand 16
Supplementary Note 2: Navigation Services 17
Supplementary Note 3: Traffic Conditions 18
Supplementary Note 4: Route Diversity 18
Supplementary Note 5: Distribution of CO_2 Emissions Across Roads 18
Supplementary Note 6: Abstract Setting Experiments 19
Supplementary Note 7: Sensitivity analysis on the duarouter parameter w 21

1 Supplementary Figures

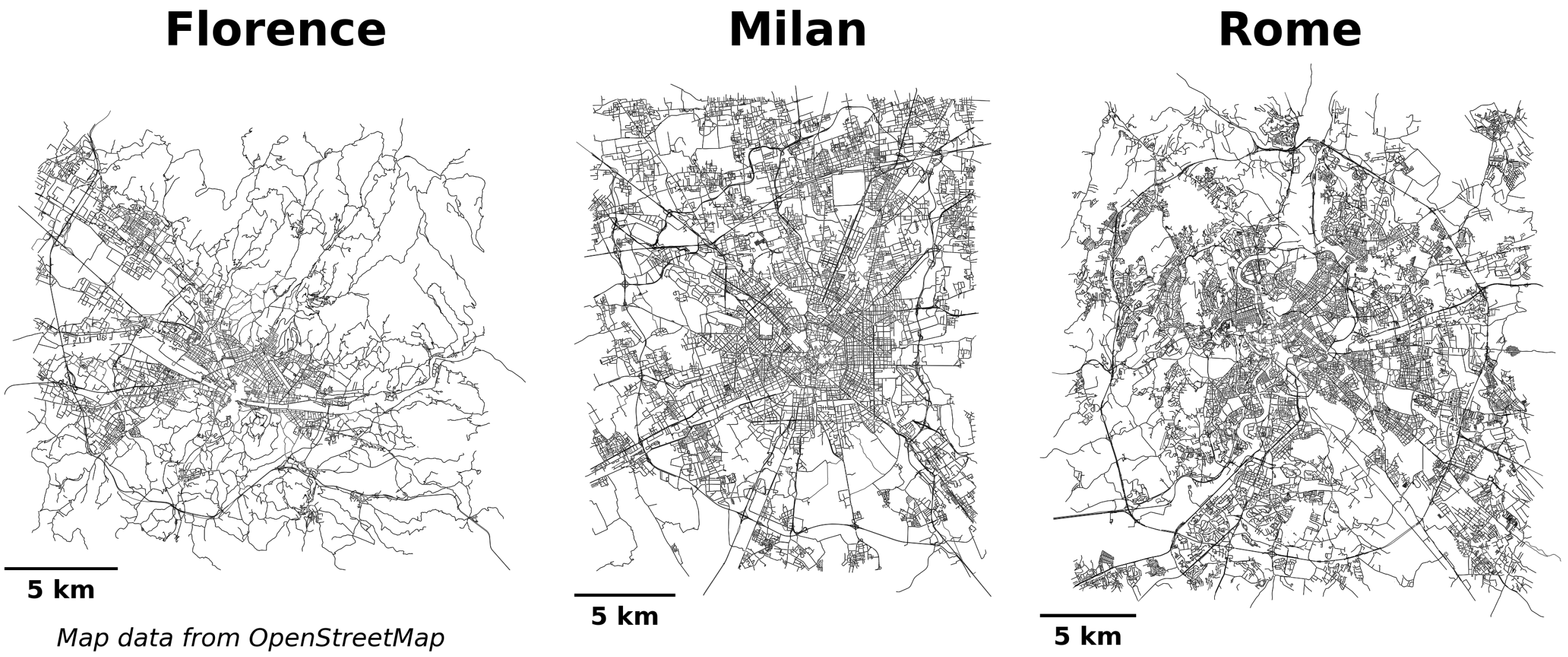

Figure S1: Road networks of Florence, Milan, and Rome extracted used in the simulations. Nodes represent intersections and edges correspond to road segments. The figure highlights differences in network size, density, and structure across cities. Road network data from OpenStreetMap (© OpenStreetMap contributors), available under the Open Database License (ODbL; `https://www.openstreetmap.org/copyright`). OpenStreetMap documentation is published under CC BY-SA 2.0 (`https://creativecommons.org/licenses/by-sa/2.0/`).

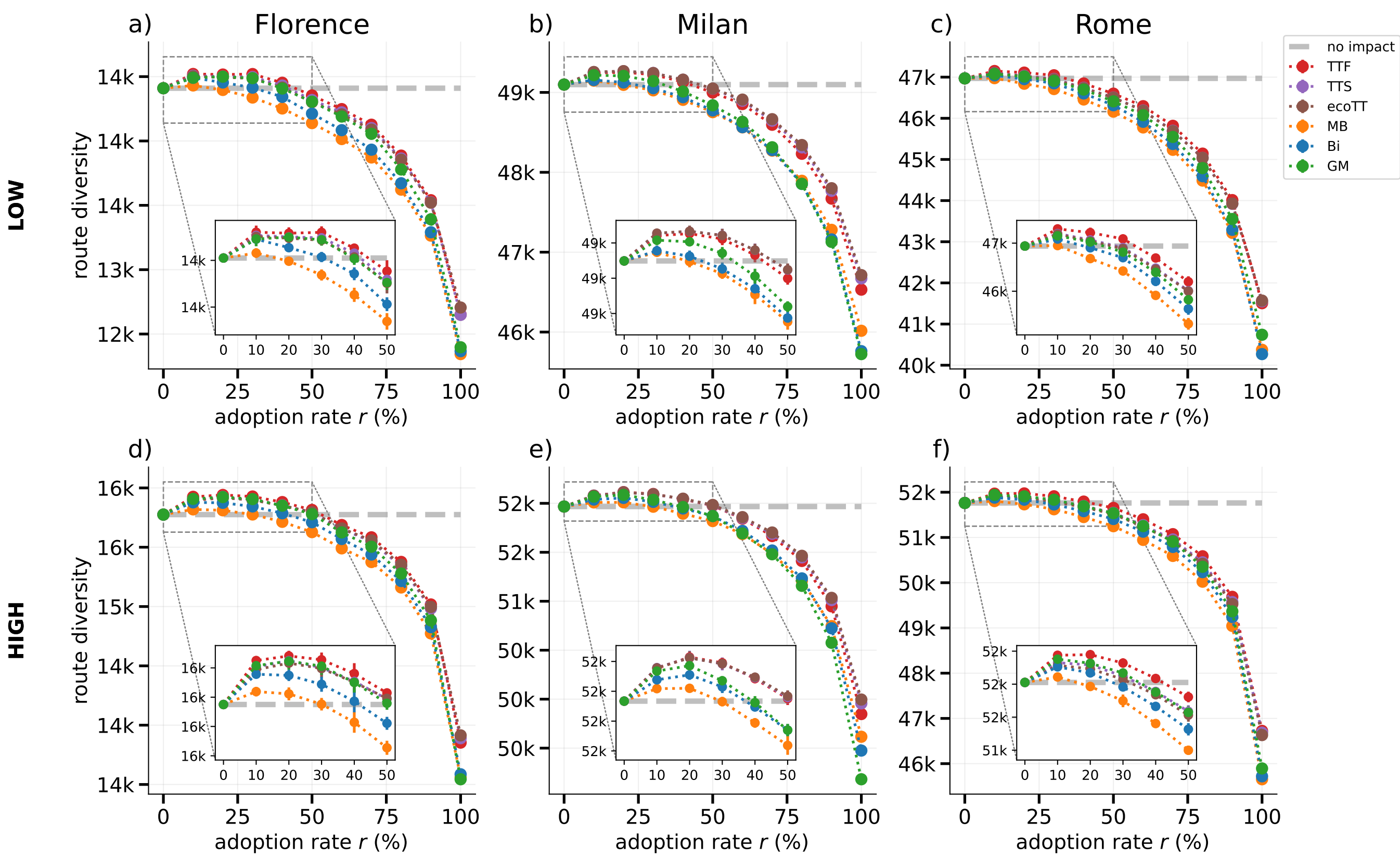

Figure S2: Service adoption rate versus route diversity at low (a-c) and high (d-f) traffic loads in Florence, Milan, and Rome. The dashed line represents the no-adoption scenario ($r = 0\%$). In the error bars, points indicate the average route diversity over ten simulations with different choices of s-routed vehicles chosen uniformly at random. Vertical bars indicate the standard deviation. The inset plots zoom on the range $r = 0\%, \ldots, 50\%$, where route diversity slightly increases. The consistency of this universal pattern is evident across different cities and varying traffic conditions.

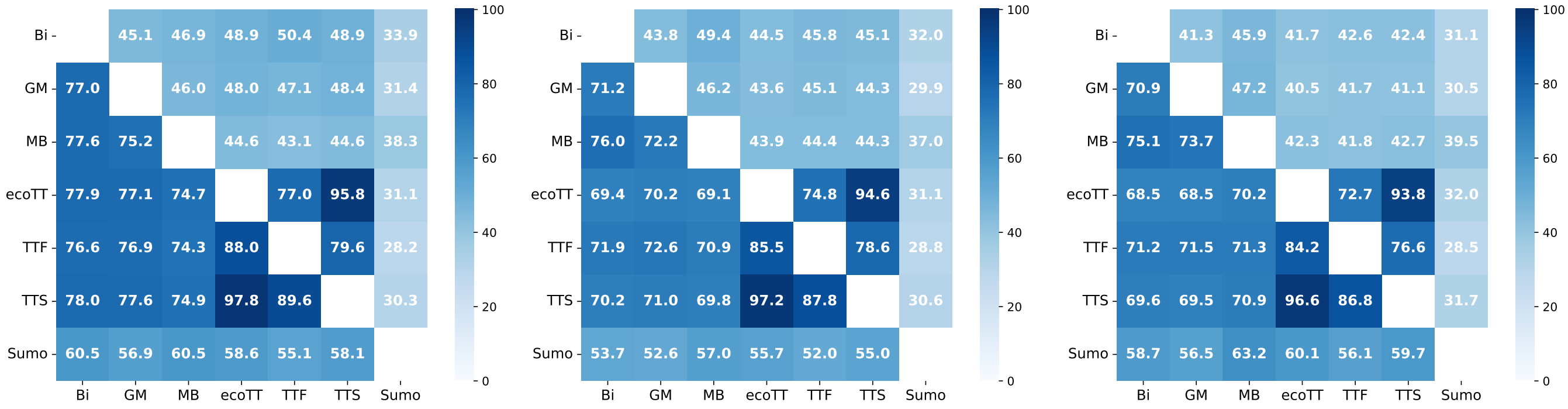

(a) Florence (b) Milan (c) Rome

Figure S3: Average overlap (below the diagonal) and percentage of exact matches (above the diagonal) of routes suggested by different navigation services for the same trips in three cities: (a) Florence, (b) Milan, and (c) Rome. For each cell below the diagonal, the value represents the average overlap computed for each route pair between two services. In addition to commercial navigation services (Bi, GM, MB, ecoTT, TTF, and TTS), we include the SUMO fastest path. Colour intensity represents the value, with darker shades indicating higher overlaps or percentages of exact matches.

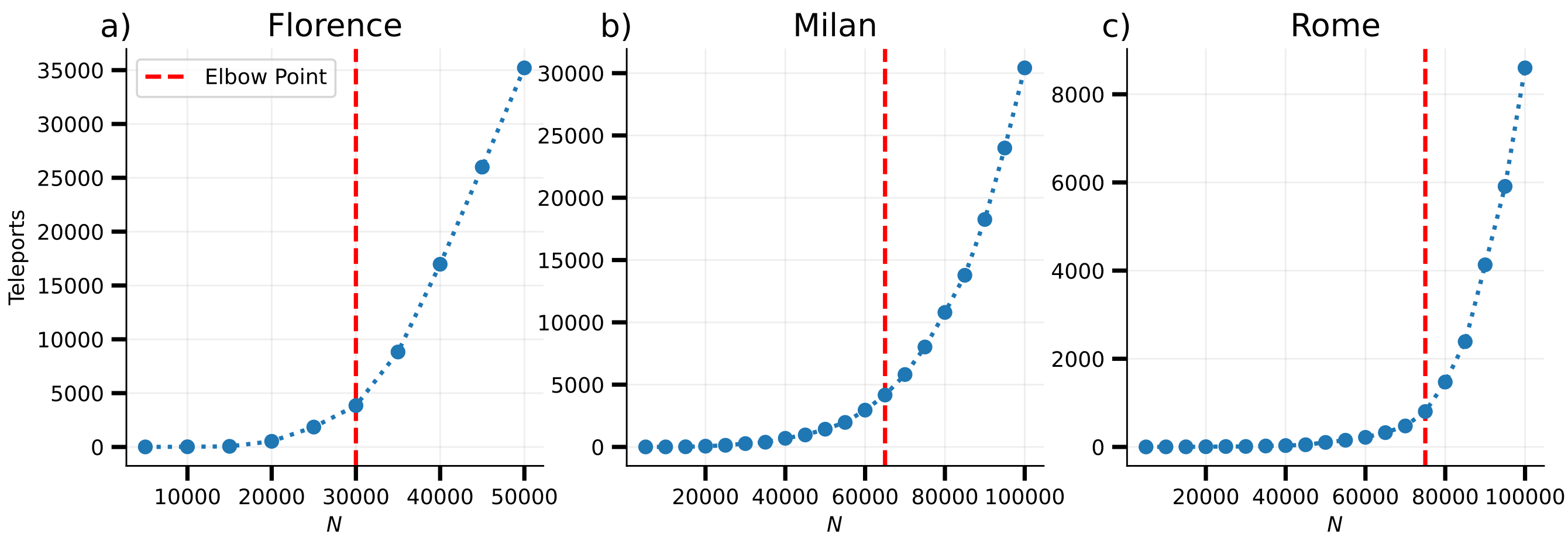

Figure S4: Number of vehicles in the simulation versus the number of SUMO teleports for Florence, Milan, and Rome. Simulations are conducted based on the mobility demand of the city and using the fastest route. The red dashed line denotes the elbow point of the curve.

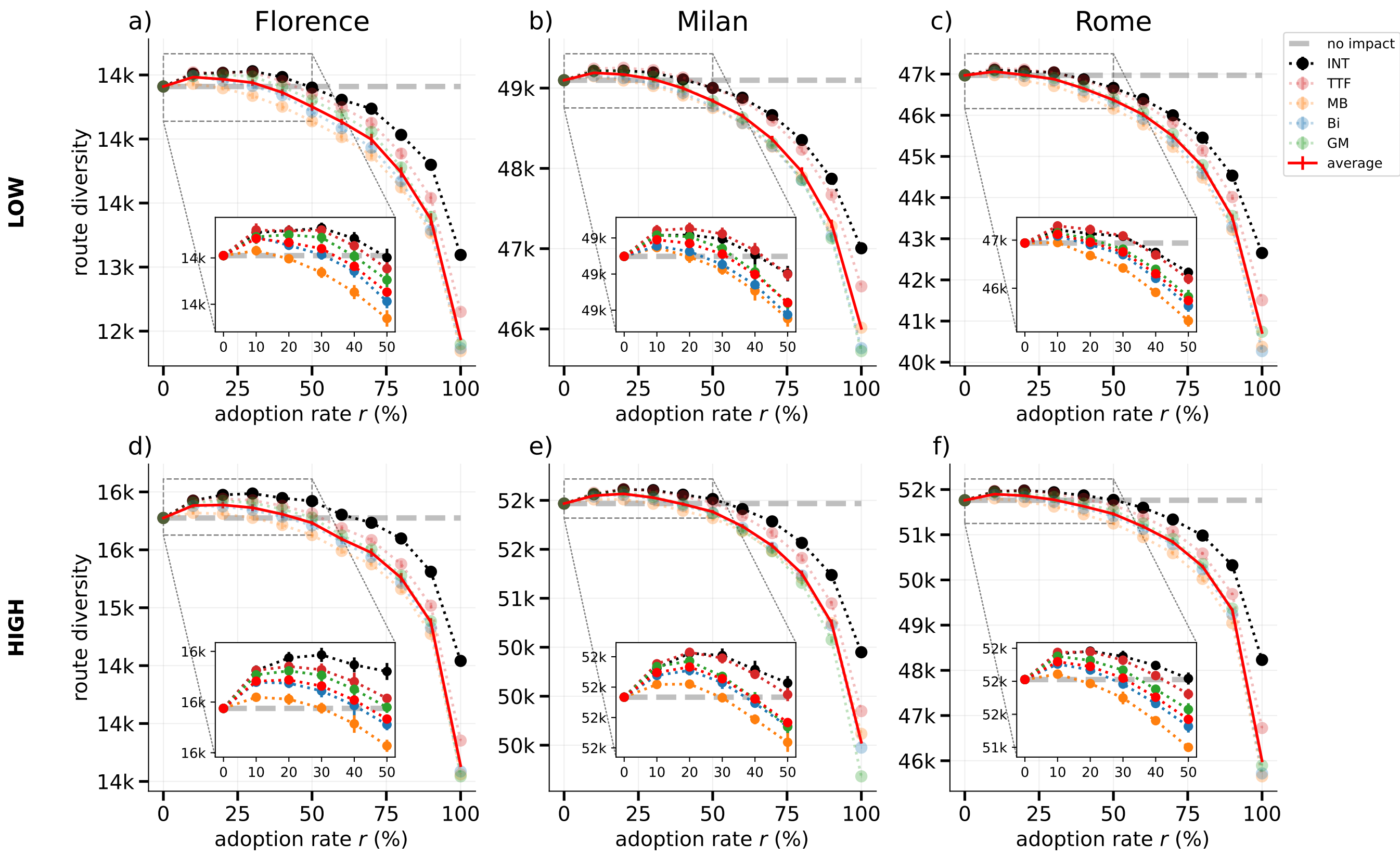

Figure S5: Service adoption rate versus route diversity at low (a-c) and high (d-f) traffic loads in Florence, Milan, and Rome. The dashed line represents the no-adoption scenario ($r = 0\%$). The black curve represents the combination scenario, while the red curve denotes the average of the curves for individual services (i.e., GM, Bi, MB, TTF). In the error bars, points indicate the average route diversity over ten simulations with different choices of s-routed vehicles chosen uniformly at random. Vertical bars indicate the standard deviation. The inset plots zoom on the range $r = 0\%, \ldots, 50\%$, where route diversity slightly increases.

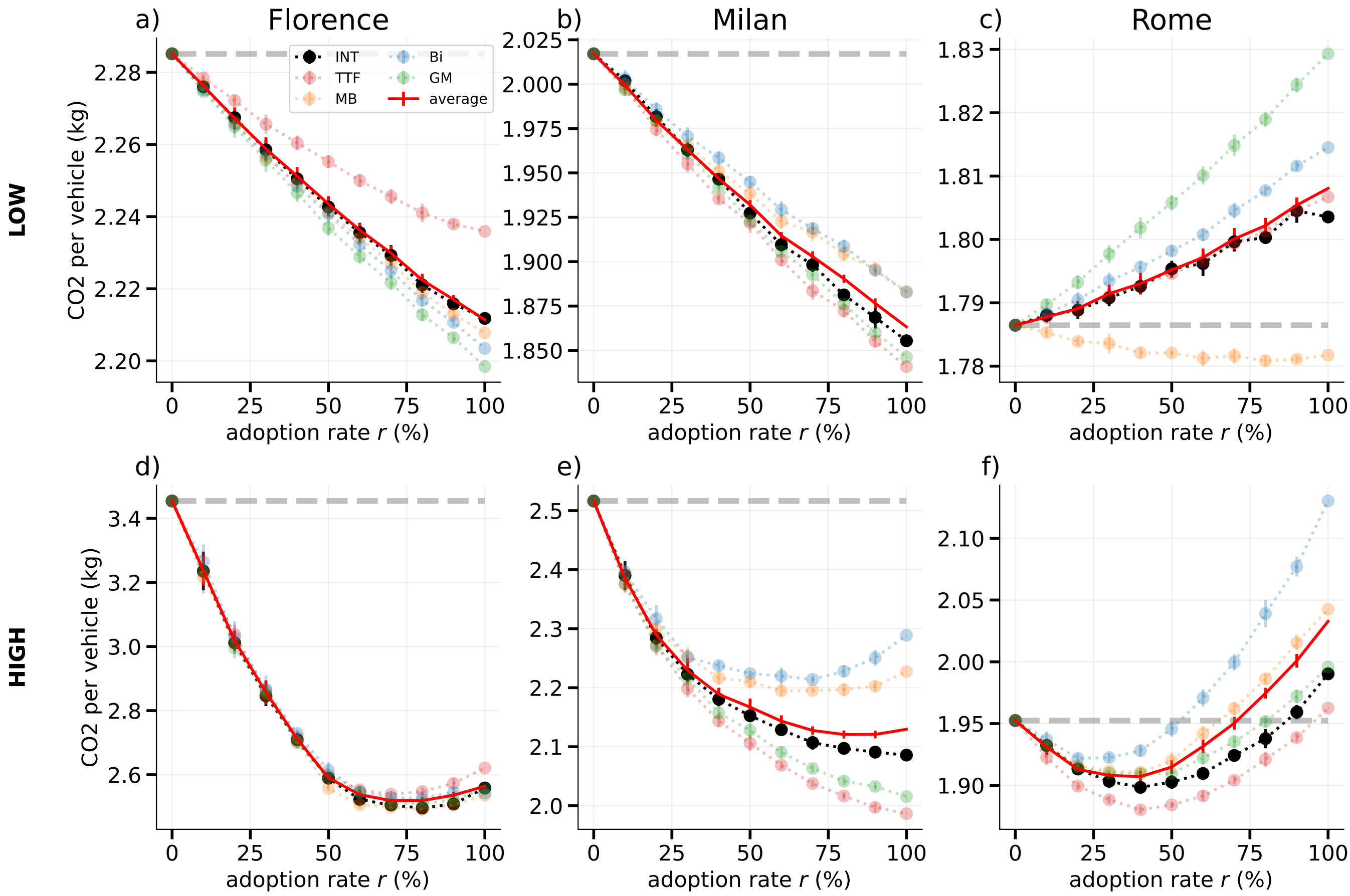

Figure S6: Service adoption rate versus average CO_2 emissions per vehicle at low (a-c) and high (d-f) traffic loads in Florence, Milan, and Rome. The dashed line represents the no-adoption scenario ($r = 0\%$). The black curve represents the combination scenario, while the red curve denotes the average of the curves for individual services (i.e., GM, Bi, MB, TTF). In the error bars, points indicate the average CO_2 emissions per vehicle over ten simulations with different choices of s-routed vehicles chosen uniformly at random. Vertical bars indicate the standard deviation.

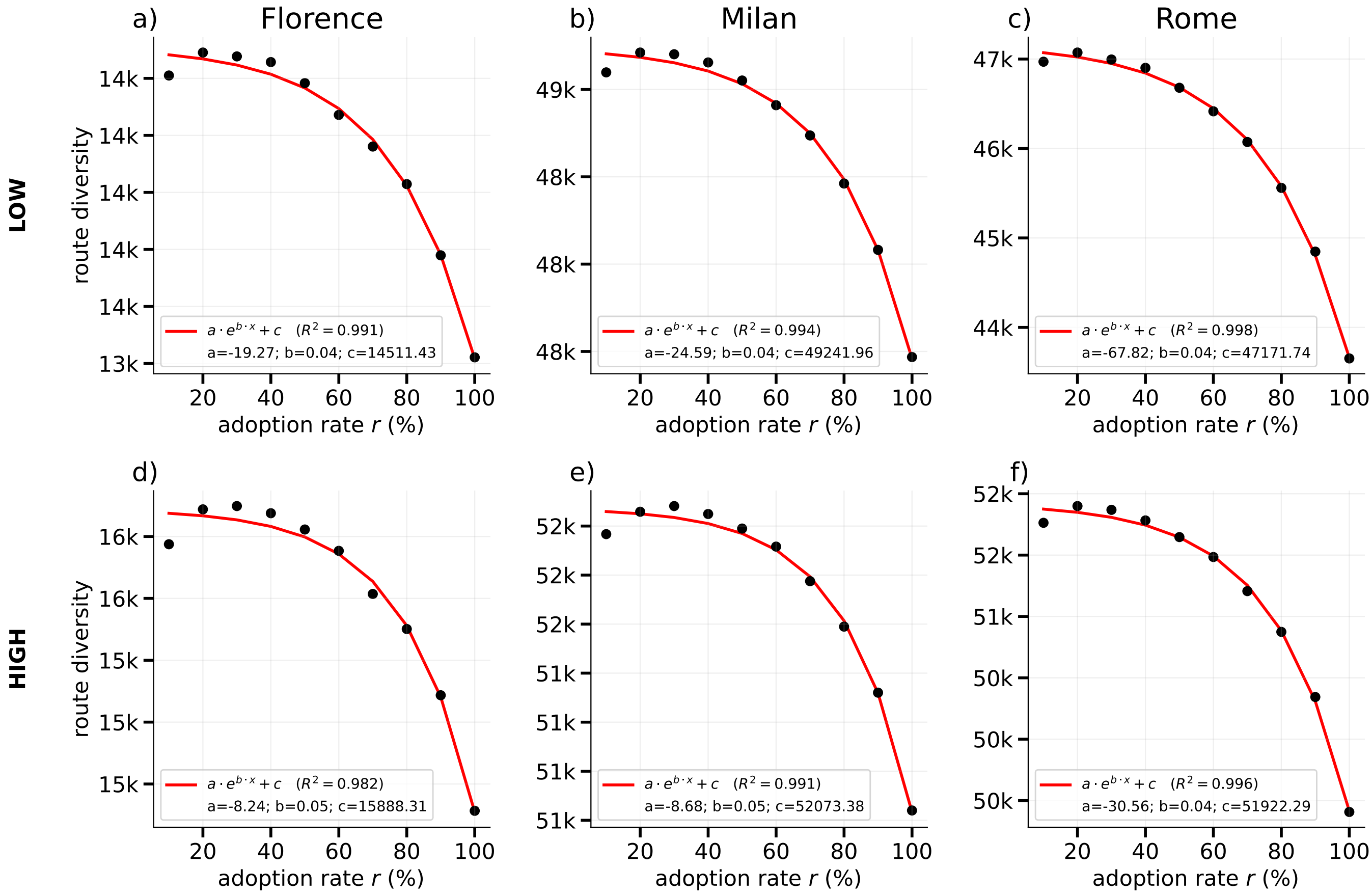

Figure S7: Service adoption rate versus route diversity at low (a-c) and high (d-f) traffic loads in Florence, Milan, and Rome. The data points represent the average route diversity across different navigation systems at varying adoption rates. The red curves illustrate the best-fitting exponential function $y = a \cdot e^{b \cdot x} + c$, with the goodness of fit (R^2) and the parameter values (a, b, c). The exponential model consistently provides the best fit across all scenarios.

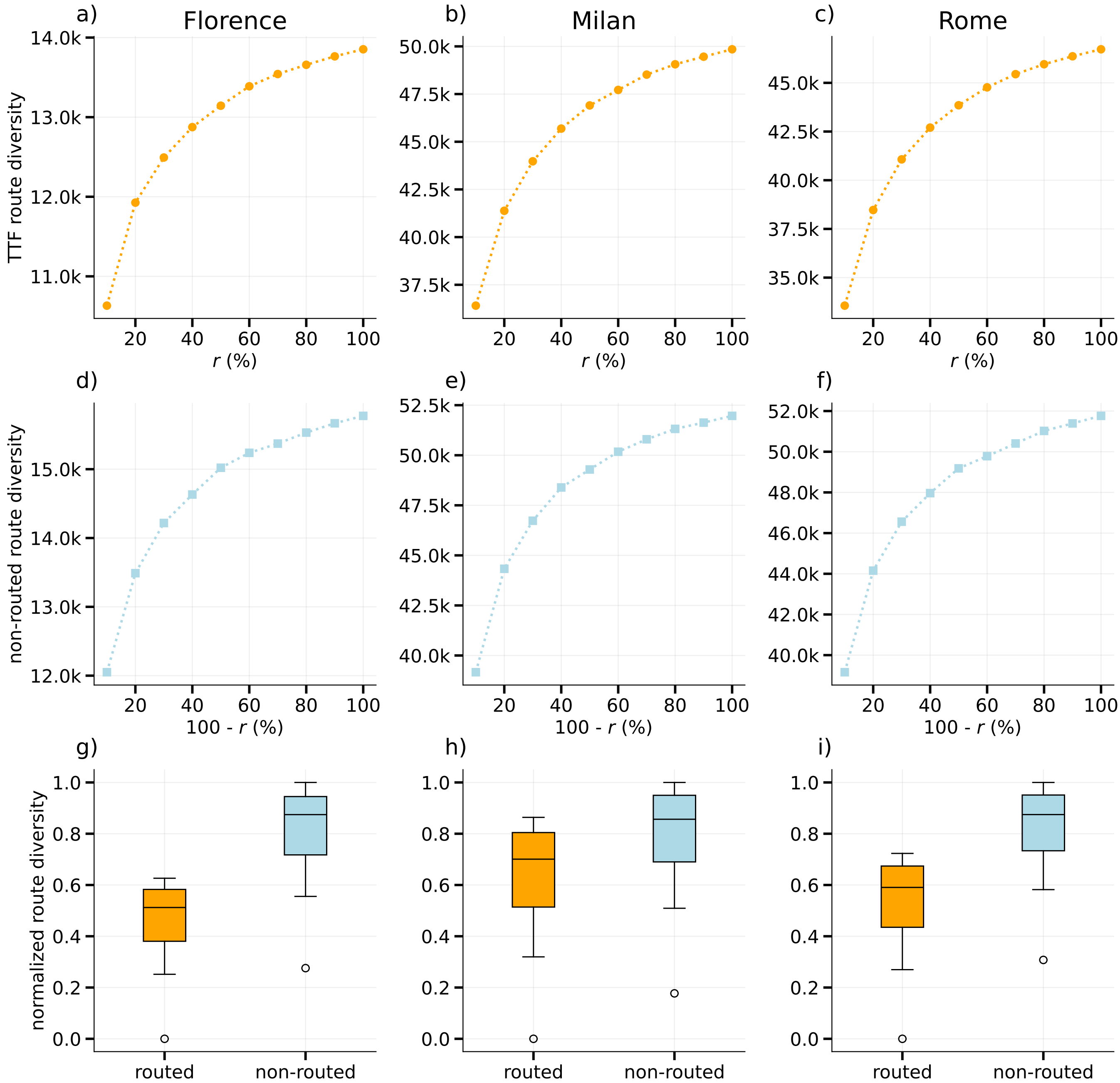

Figure S8: The relationship between service adoption rate and route diversity for TTF-routed vehicles in Florence, Milan, and Rome (a-c). The corresponding trends for non-routed vehicles (d-f). Route diversity increases for both routed and non-routed groups as the number of vehicles in each group increases with their respective adoption rates. The distribution of normalised route diversity for routed (orange) and non-routed (blue) vehicle groups across the three cities (g-i). Routed groups exhibit lower route diversity than non-routed groups, indicating a higher degree of route conformity among vehicles using navigation services.

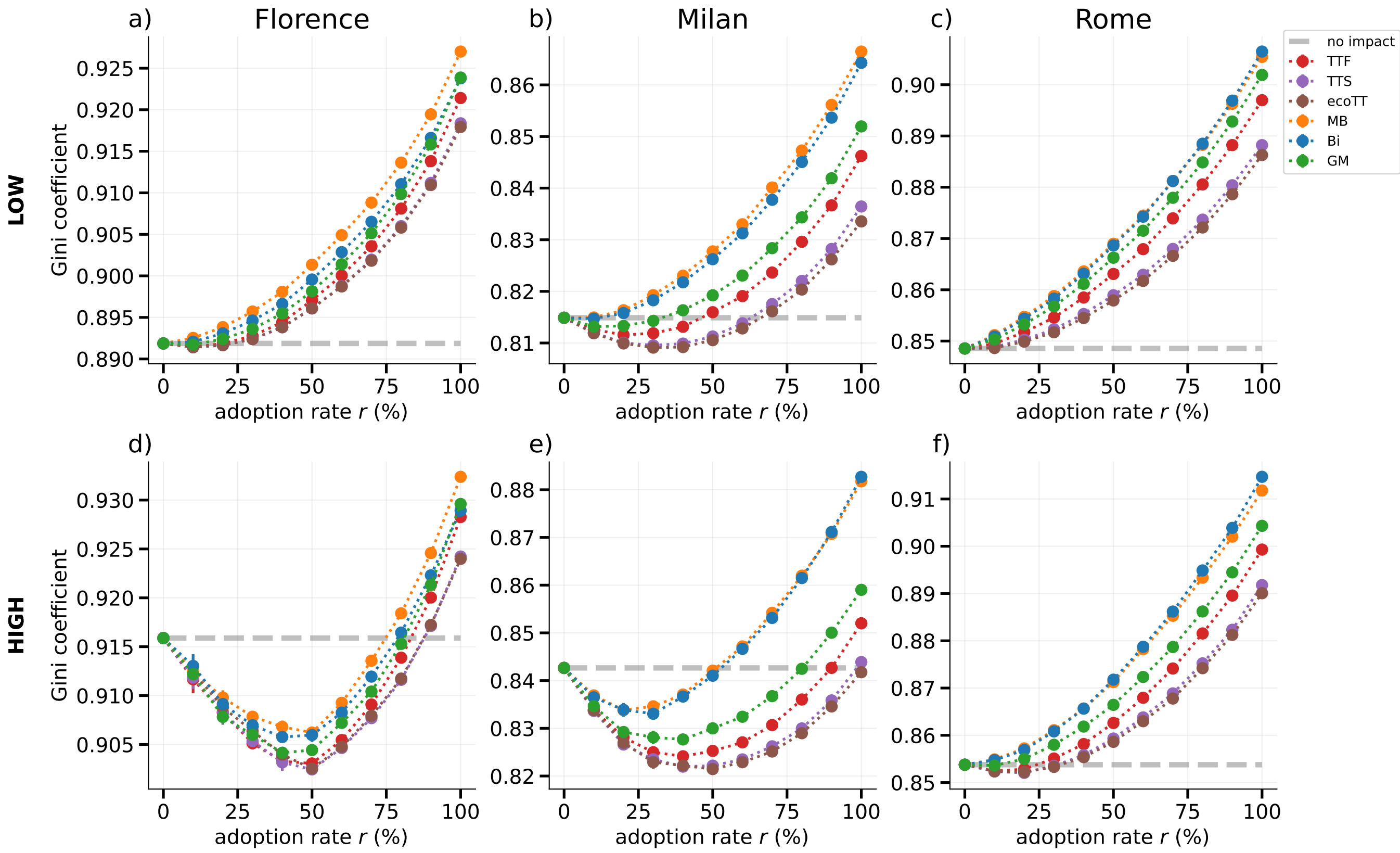

Figure S9: Service adoption rate versus the Gini coefficient of CO_2 Emissions on roads at low (upper row) and high traffic loads (lower row) in Florence, Milan, and Rome. The dashed line represents the no-adoption scenario ($r = 0\%$). In the error bars, points indicate the average Gini over ten simulations with different choices of s-routed vehicles chosen uniformly at random. Vertical bars indicate the standard deviation.

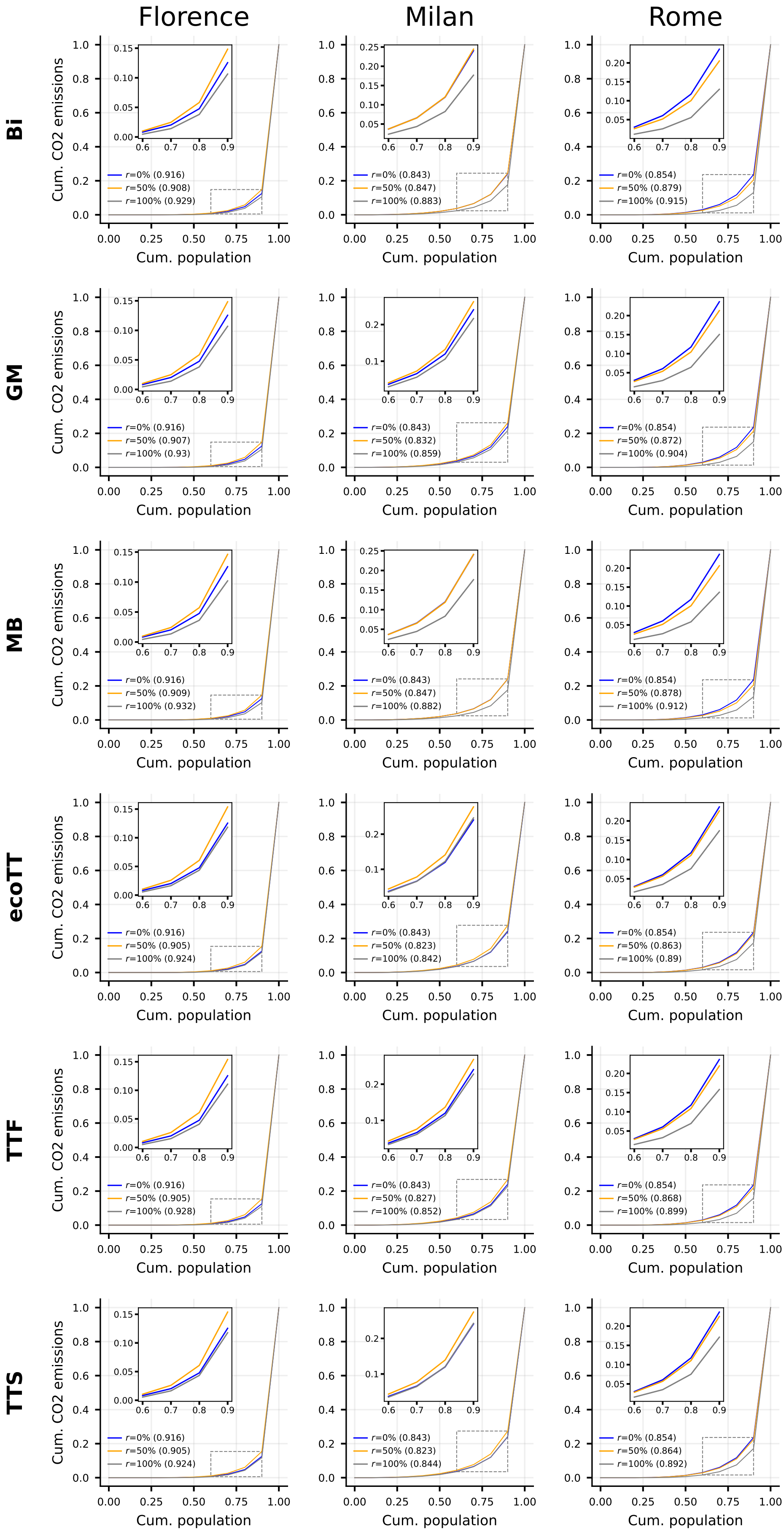

Figure S10: Lorenz curves of the cumulative share of CO_2 emissions as a function of the cumulative share of the road network, ordered from least to most polluting road segments. Each row corresponds to a different navigation service, and columns show results for Florence, Milan, and Rome. Curves are reported for three adoption rates (r = 0% [blue], 50% [orange], and 100% [grey]); the corresponding Gini coefficients are shown in parentheses.

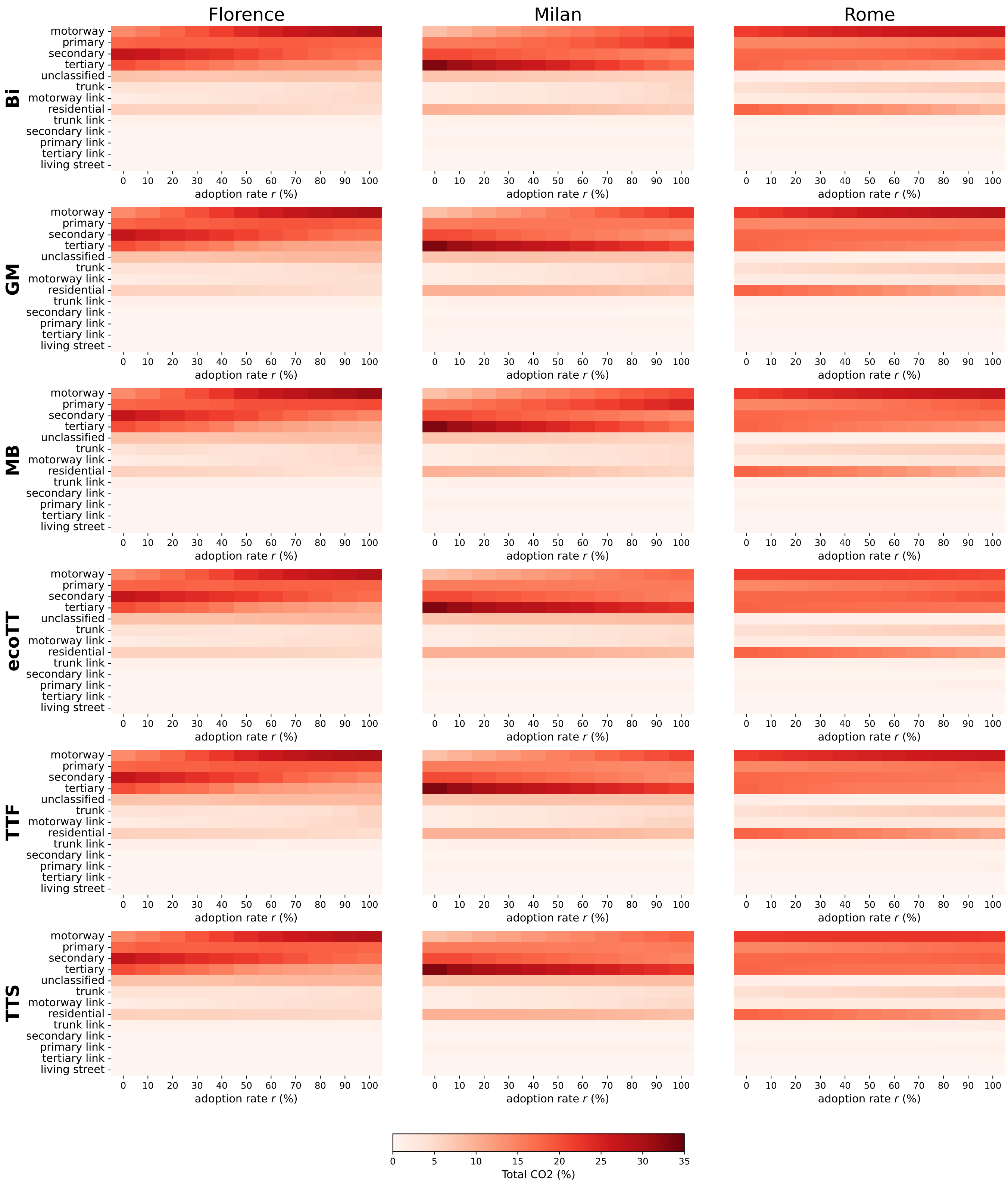

Figure S11: Share of total CO_2 emissions by road type as a function of navigation service adoption. Each row corresponds to a navigation service, and columns show results for Florence, Milan, and Rome. Colours indicate the percentage of total CO_2 emissions attributed to each OpenStreetMap road category (e.g., `motorway`, `primary`, `residential`) at different adoption rates. Values are averaged over 10 simulation runs. Darker red indicates a higher share of total CO_2 emissions.

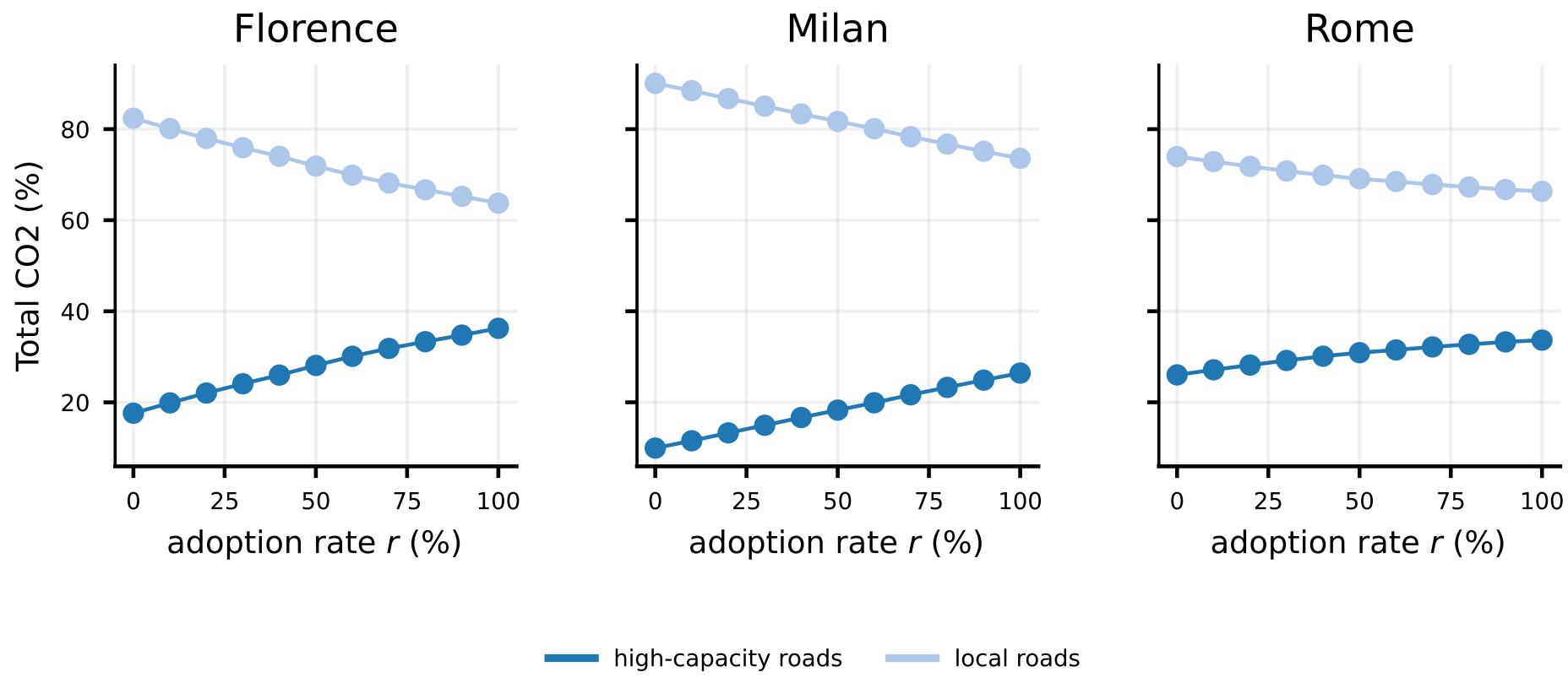

Figure S12: Share of total CO_2 emissions produced on high-capacity roads (e.g., highways and arterial corridors) and local roads as adoption increases.

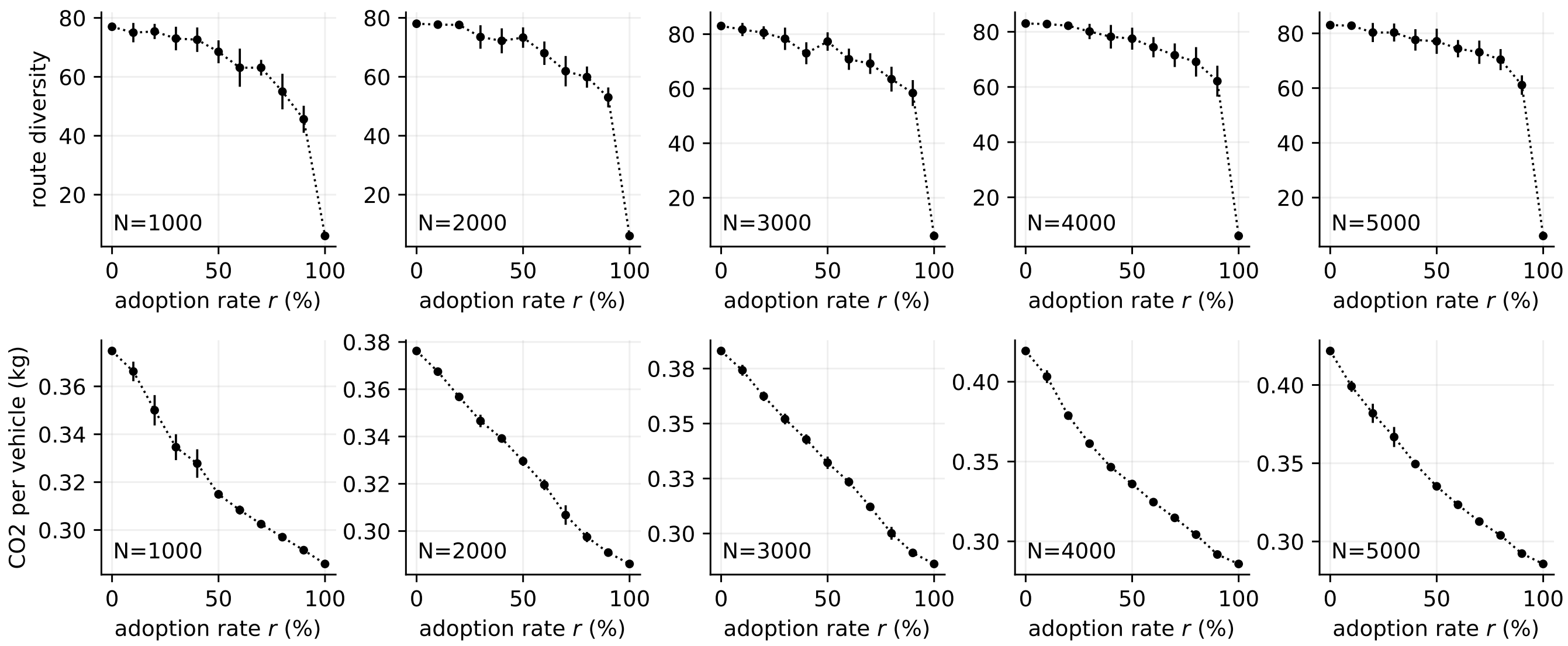

Figure S13: Route diversity (top) and average CO_2 emissions per vehicle (bottom) as a function of adoption rate r in a 10×10 grid with a single OD flow. Results are shown for different values of N (number of vehicles). Vertical bars indicate the standard deviation.

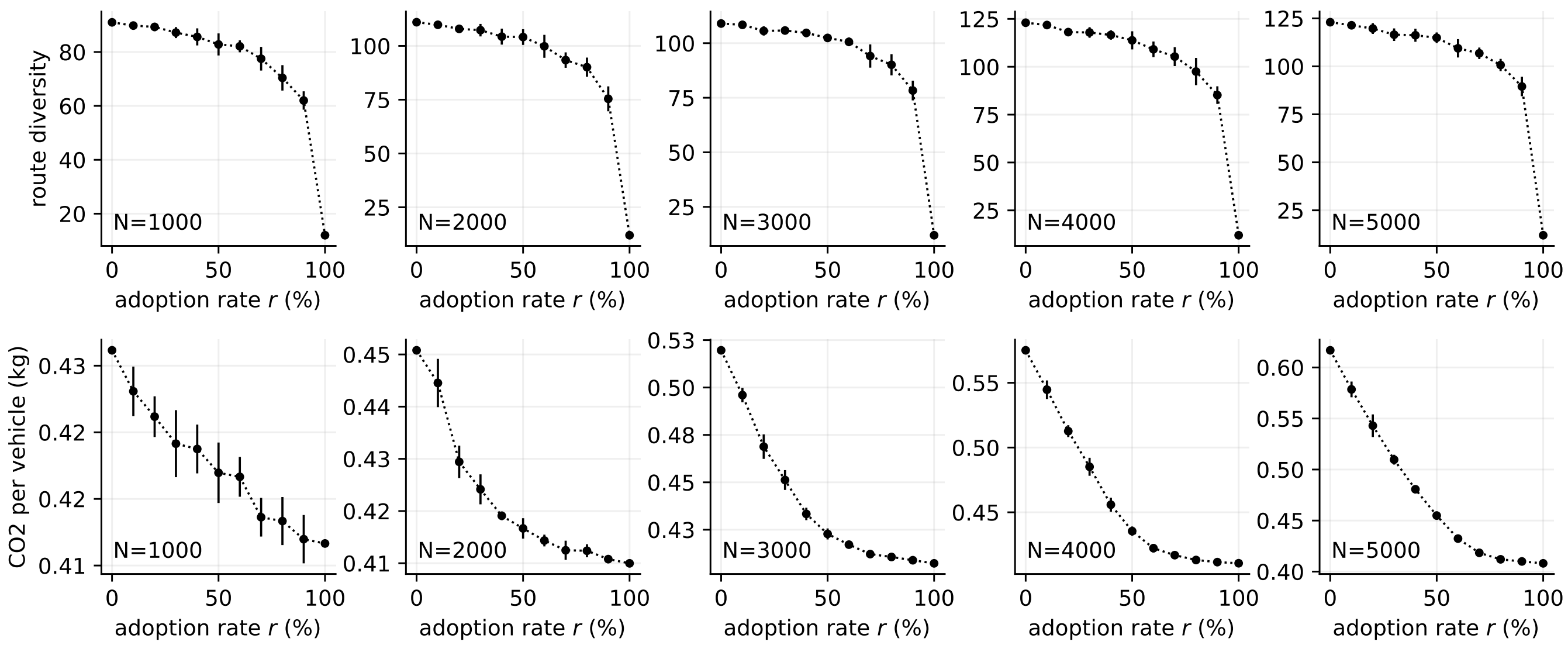

Figure S14: Route diversity (top) and average CO_2 emissions per vehicle (bottom) as a function of adoption rate r in a 10×10 grid with two intersecting OD flows. Results are shown for different values of N (number of vehicles). Vertical bars indicate the standard deviation.

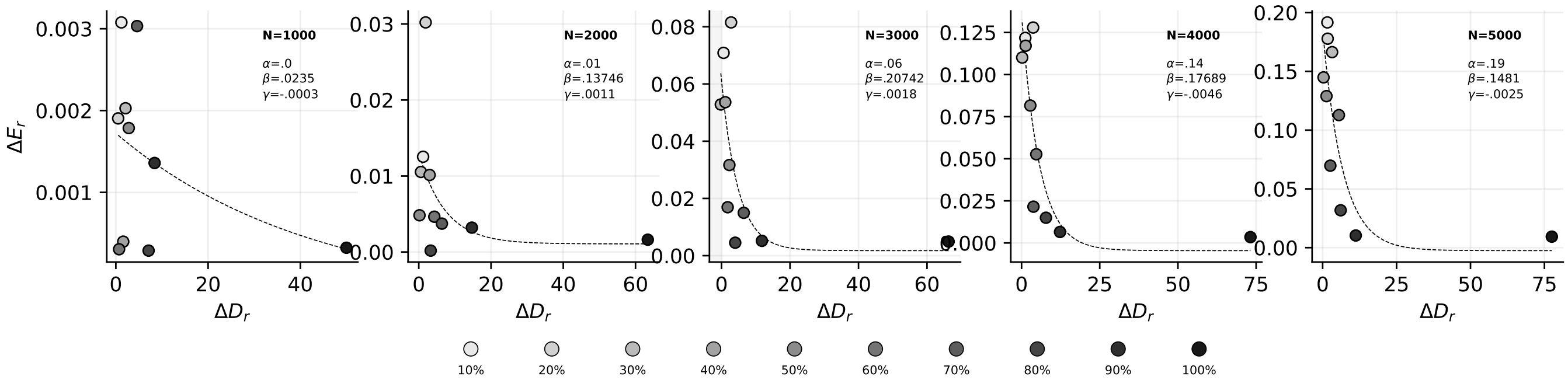

Figure S15: Marginal change in route diversity (ΔD_r) versus marginal change in CO_2 emissions (ΔE_r) as a function of adoption rate r in a 10×10 grid with two intersecting origin-destination flows. Results are shown for different values of N (number of vehicles). Vertical bars indicate the standard deviation.

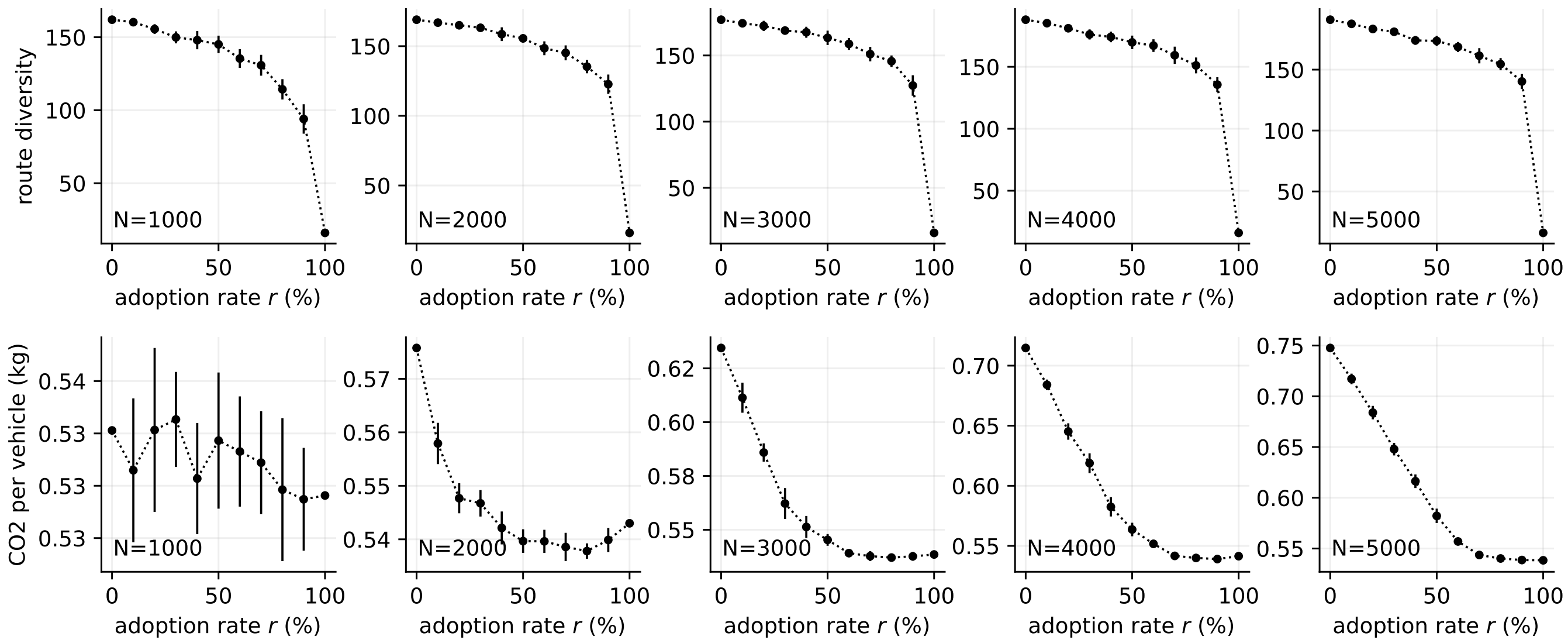

Figure S16: Route diversity (top) and average CO_2 emissions per vehicle (bottom) as a function of adoption rate r in a 14×14 grid with two intersecting OD flows. Results are shown for different values of N (number of vehicles). Vertical bars indicate the standard deviation.

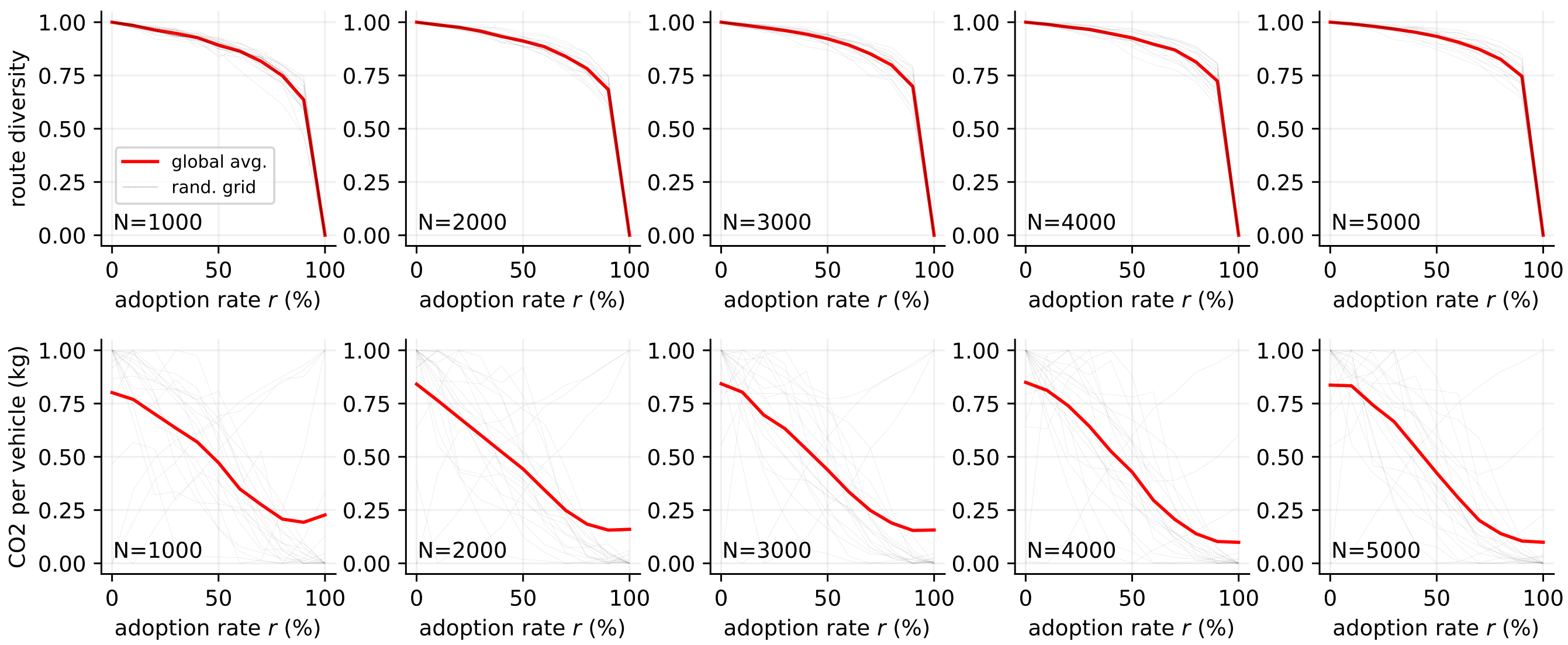

Figure S17: Normalised route diversity (top) and normalised total CO_2 emissions (bottom) as a function of adoption rate r in small randomised grid networks. Results are shown for different values of N (number of vehicles). Opaque lines represent individual randomised grid instances, and the red line indicates their average.

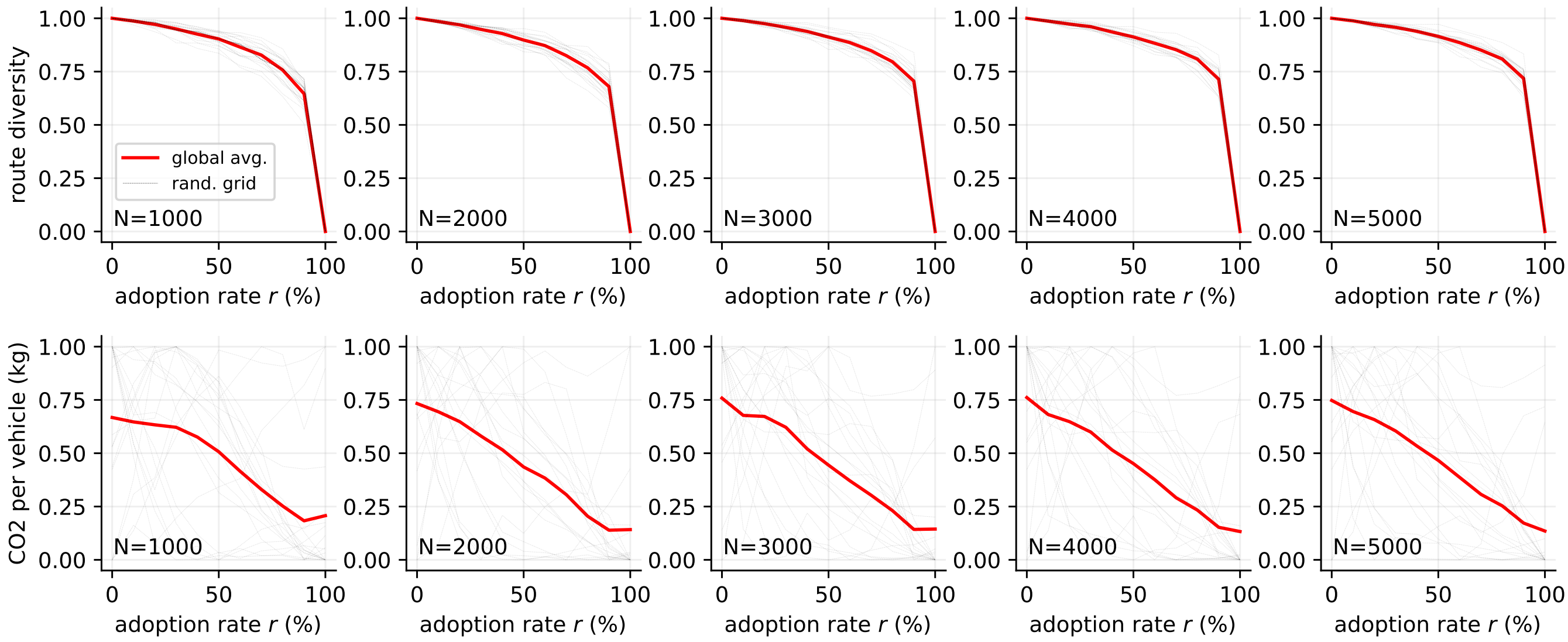

Figure S18: Normalised route diversity (top) and normalised total CO_2 emissions (bottom) as a function of adoption rate r in large randomised grid networks. Results are shown for different values of N (number of vehicles). Opaque lines represent individual randomised grid instances, and the red line indicates their average.

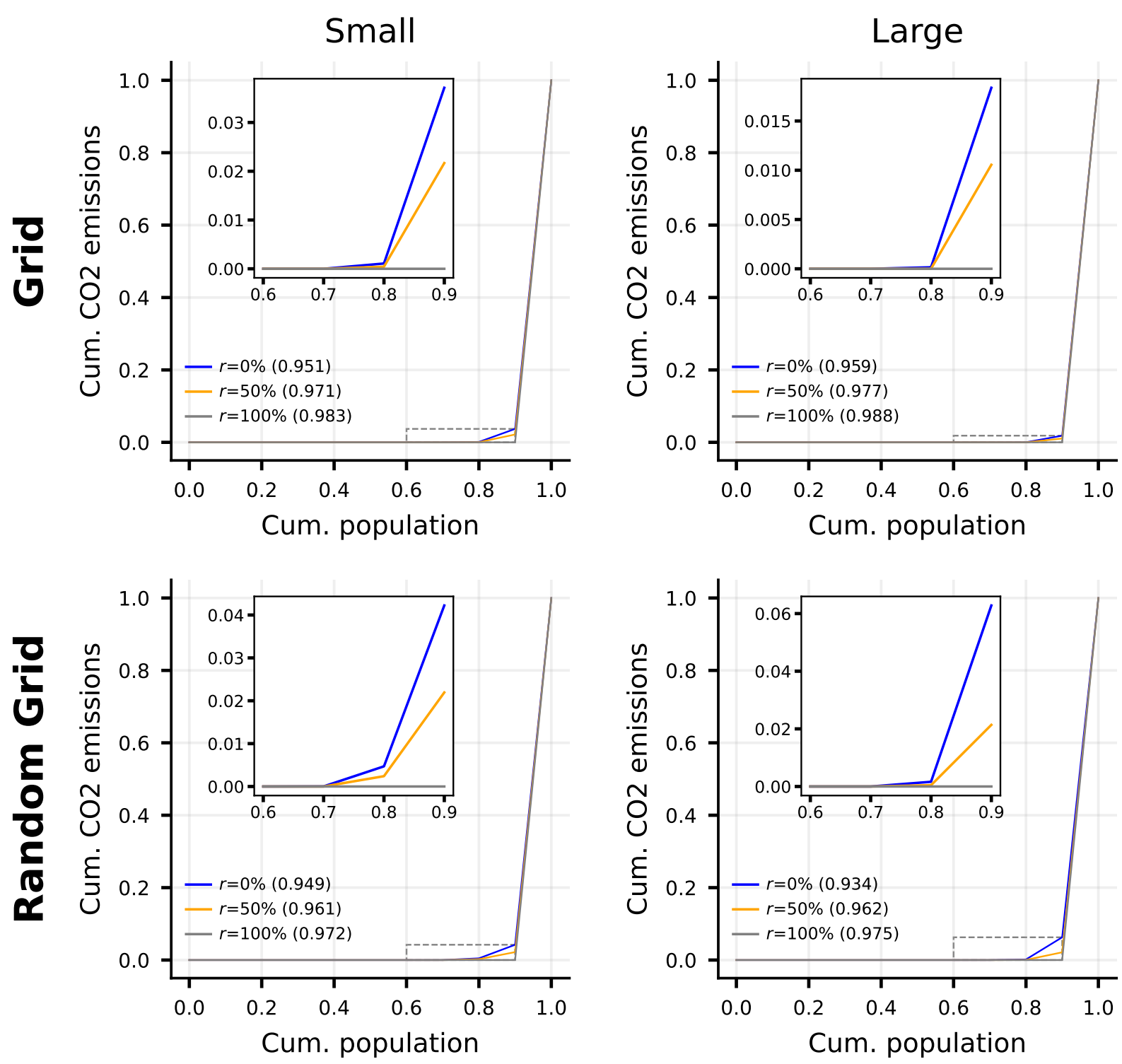

Figure S19: Lorenz curves of the cumulative share of CO_2 emissions as a function of the cumulative share of the road network, ordered from least to most polluting road segments. Columns correspond to small (left) and large (right) networks, while rows distinguish regular grid (top) and randomised grid (bottom) configurations. Results are shown for three adoption rates (r = 0% [blue], 50% [orange], and 100% [grey]). The corresponding Gini coefficients are reported in parentheses.

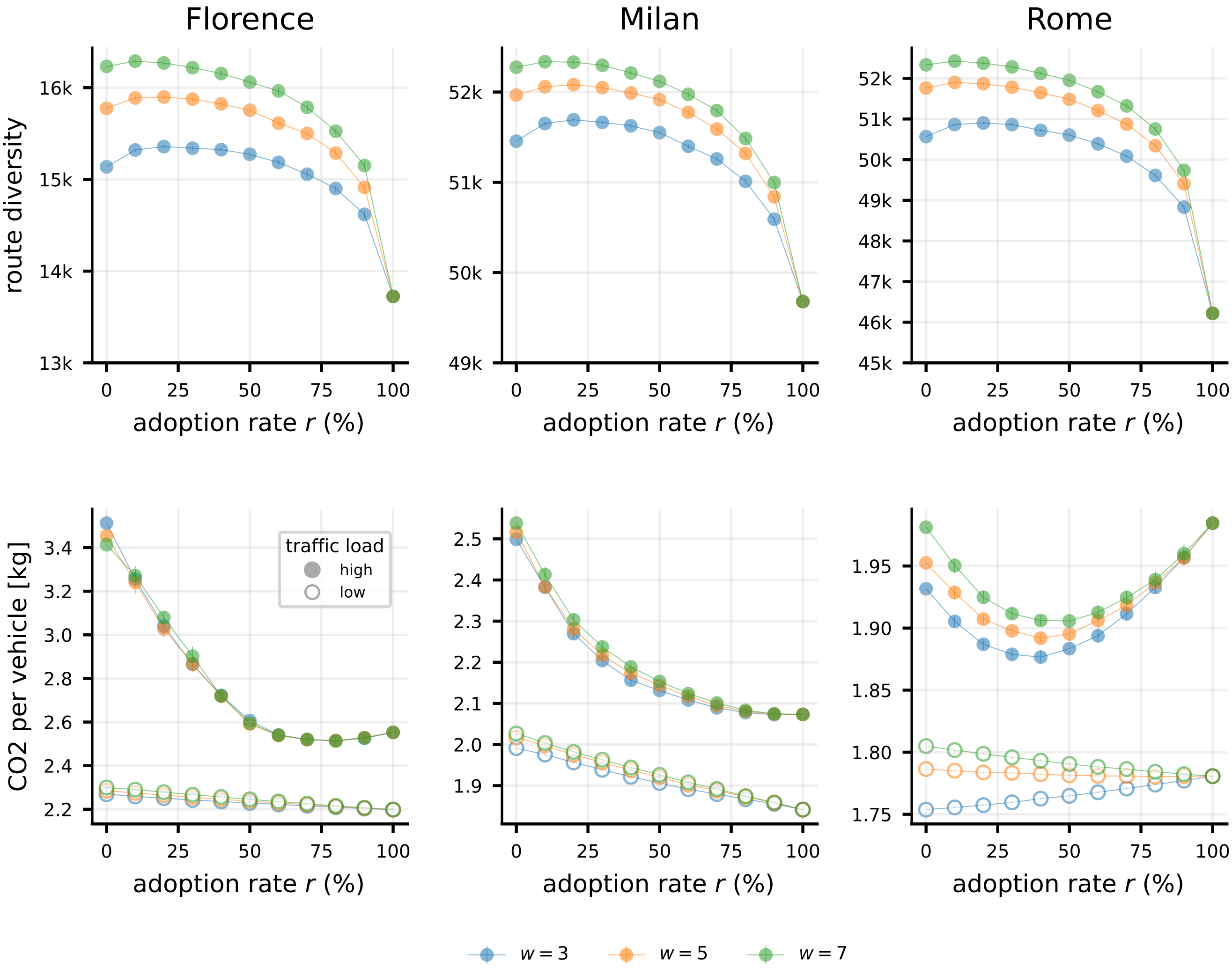

Figure S20: Service adoption rate r versus route diversity (top row) and average CO_2 emissions per vehicle (bottom row) for $w = 3, 5, 7$ in Florence, Milan, and Rome. Results are averaged across all navigation services. Vertical bars indicate the standard deviation. In the bottom row, filled markers represent high traffic conditions, and white-filled markers indicate low traffic conditions. Despite slight shifts in absolute values, the shape and trend of the curves remain consistent across all settings, confirming the robustness of the main findings with respect to the choice of w.

2 Supplementary Tables

City	$\lvert V\rvert$	$\lvert E\rvert$	$\frac{\lvert E\rvert}{\lvert V\rvert}$	Total Street Length (km)	Avg. Street Length (m)	Total Edge Length (km)	Avg. Edge Length (m)	Avg. Circuity
Florence	12,303	24,719	2.009	2157.73	125.27	3434.05	138.92	1.104
Milan	31,354	62,738	2.001	4584.81	98.57	6511.90	103.80	1.053
Rome	37,483	76,280	2.035	5852.46	110.78	8794.08	115.29	1.069

Table S1: Structural properties of the road networks in Florence, Milan, and Rome. We report node and edge counts, connectivity, length-based infrastructure measures, and average circuity.

		low traffic		high traffic	
		**best**	**100%**	**best**	**100%**
Florence	Bi	-	3.57%	26.83% (r=80%)	26.40%
	MB	-	3.38%	27.87% (r=80%)	26.56%
	ecoTT	-	5.00%	27.40% (r=80%)	26.76%
	TTF	-	2.15%	26.49% (r=70%)	24.11%
	TTS	-	4.87%	27.55% (r=80%)	26.75%
	GM	-	3.79%	27.33% (r=70%)	26.01%
Milan	Bi	-	6.66%	12.01% (r=70%)	9.05%
	MB	-	6.64%	12.78% (r=60%)	11.48%
	ecoTT	-	11.13%	-	22.13%
	TTF	-	8.73%	-	21.05%
	TTS	-	10.51%	-	22.04%
	GM	-	8.47%	-	19.91%
Rome	Bi	∅	-1.57%	1.57% (r=20%)	-9.10%
	MB	0.31% (r=80%)	0.26%	2.14% (r=40%)	-4.61%
	ecoTT	-	3.69%	5.20% (r=70%)	3.69%
	TTF	∅	-1.13%	3.70% (r=40%)	-0.52%
	TTS	-	3.09%	4.91% (r=60%)	2.92%
	GM	∅	-2.40%	2.21% (r=40%)	-2.22%

Table S2: Reduction in total CO_2 emissions for various navigation services in Florence, Milan, and Rome compared to the baseline scenario (no vehicle routed). Positive values indicate a reduction, while negative values indicate an increase. The table includes data for low and high traffic conditions. Values are reported at 100% and at the point of maximum reduction (best), including the percentage of best reduction. In the "best" column, a "-" indicates the maximum reduction is achieved at 100%, and a ∅ means the navigation service is never beneficial.

3 Supplementary Notes

Supplementary Note 1: GPS data and Mobility Demand

An urban mobility demand D is a time-ordered collection of trips characterised by their origin, destination, and departure time. To estimate the mobility demand, we rely on the vehicular GPS trajectory dataset provided by Octo, a company that offers a data collection service for insurance companies. This dataset describes the trajectories of thousands of vehicles for various Italian localities over the span of an entire year. While the market penetration of the dataset varies, it generally represents between 2% and 5% of the total registered vehicles.

Our analysis focuses on Florence, Milan, and Rome, chosen for the availability of GPS traces suitable for computing Origin-Destination (OD) matrices and for their diverse sizes and road network structures (see Table S1 and Figure S1). The raw dataset includes 7,102,351 GPS points from 24,640 vehicles in Florence, 143,698,720 GPS points from 106,456 vehicles in Milan, and 26,801,872 GPS points from 30,763 vehicles in Rome.

We first performed a pre-processing step to extract segmented trajectories representing semantic journeys from the raw GPS dataset. Next, to compute the OD matrix reflecting a city's traffic patterns, we discretise the city into 1 km^2 squared tiles using scikit-mobility [1]. We then filter and aggregate the segmented trips into flows to derive OD matrices that reflect the cities' traffic patterns.

Pre-processing steps. We pre-process the raw GPS dataset to obtain a collection of segmented trajectories (i.e., trips) by performing the following operations:

- We consider only the GPS points with the maximum sampling quality to ensure the highest accuracy and reliability of the data, reducing the risk of errors due to poor signal quality or inaccurate location measurement;
- We eliminate noise by filtering out GPS points with speeds exceeding 250 km/h [2];
- We use a stop detection algorithm [2] to segment each trajectory into sub-trajectories based on identified stops. A stop is identified when a vehicle remains within a distance of 0.2 km from a trajectory point for at least 20 minutes;
- We considered only trips that both start and end within the predefined area of interest (i.e., the city's bounding box). For trips that start or end outside this area but traverse it, we used the first entry point within the area as the origin and the last exit point as the destination. This method preserves commuter trips originating or ending outside the city.

Flow filtering. To compute the flows from the collection of trips extracted from the raw GPS data, we filter the trips as follows:

- We consider only trips with a duration between 5 and 60 minutes to focus on typical urban commutes. Short trips (less than 5 minutes) often involve anomalies such as GPS errors or very brief stops, which do not provide meaningful data on urban traffic patterns. Conversely, trips longer than 60 minutes may include long-distance travel or other atypical journeys.
- We discard outlier days by analysing their Pearson correlation with aggregate mobility patterns. Aggregate mobility refers to the sum of all flows within a specific time period. Specifically, we compare the mobility flow of each day to the aggregate mobility flow of all other days that fall on the same day of the week (e.g., comparing all Mondays). A day is classified as an outlier if its correlation with the aggregate mobility flow is below a certain threshold, defined as the average correlation minus the standard deviation. By excluding these outlier days, we effectively remove anomalies and inconsistent data, ensuring a more accurate representation of typical mobility patterns.
- We consider only trips that depart on Wednesdays. Our analysis shows that aggregate mobility during the morning peak hours of weekdays is highly correlated (average Pearson correlation of 0.780 in Florence, 0.880 in Milan, and 0.772 in Rome), indicating that traffic patterns are consistent across working days. In contrast, there is a low correlation between weekend and weekday traffic patterns. We chose Wednesday because it is a mid-week working day.
- We filter out infrequent flows, retaining only those that occur regularly. We use a data-driven approach to determine the frequency threshold (i.e., the minimum number of trips to define a flow as frequent). We examine the number of non-zero flows across various frequency thresholds, and we identify the elbow point of this function, where the number of flows decreases considerably. This elbow point represents the optimal frequency threshold, allowing us to remove noise flows while preserving meaningful data.
- Finally, we focus exclusively on trips that depart during the morning peak, a period characterised by intense traffic congestion. This time frame offers a robust scenario for analysing vehicular movement patterns and understanding the impact of navigation services on emissions within typical urban traffic conditions. Based on

this procedure, the morning peak hours are identified as 8–9 AM for Florence, 7–8 AM for Milan, and 8–9 AM for Rome.

After the flow filtering, the datasets used to generate an OD-matrix M for each city include:

- Florence: 5,477 trips from 1,184 vehicles, resulting in 616 distinct flows;
- Milan: 113,323 trips from 15,997 vehicles, resulting in 11,967 distinct flows.
- Rome: 13,647 trips from 1,965 vehicles, resulting in 1,757 distinct flows.

Mobility demand generation. To generate a mobility demand D consisting of N trips from the OD matrix M, we follow these steps to ensure that D accurately represents the spatial distribution of trips within the city based on the observed data.

- **Random Trip Selection**: For each vehicle v, we randomly select a trip $T_v = (e_o, e_d)$. The origin-destination pair (o, d) is chosen based on the probabilities $p_{o,d} \propto m_{o,d}$, where $m_{o,d}$ represents the elements of the OD matrix M. This ensures that trips are selected in proportion to their frequency in the OD matrix;
- **Edge Selection within Tiles**: Once an origin-destination pair (o, d) is selected, we uniformly choose two edges e_o and e_d within the respective tiles o and d from the road network G. This step ensures that the trips are mapped to specific routes within the city's road network, reflecting realistic travel paths.

Supplementary Note 2: Navigation Services

We consider several navigation services with public APIs, including Google Maps (GM), Mapbox (MB), Bing Maps (Bi), TomTom fastest route (TTF), TomTom shortest route (TTS), and TomTom Eco-routing (EcoTT). These services suggest a route between an origin o and a destination d at a time t, considering various factors such as the typical traffic conditions at the time t when the trip (o, d, t) departs.

The APIs provide recommendations based on real-time, real-world traffic conditions, which can be influenced by unpredictable events like accidents or construction work. To ensure consistency with our simulated traffic, we request routes for future departure times t relative to the time of the request, forcing the navigation services APIs to use historical traffic information instead of real-time traffic conditions.

The route recommendations from the APIs are typically provided as sequences of GPS points. We employed a map-matching procedure to integrate these routes into the SUMO simulator. Specifically, we applied the state-of-the-art Longest Common Subsequence (LCSS) algorithm [3] to convert the sequence of GPS points into a sequence of connected edges in the SUMO road network, accurately representing the suggested routes.

Including multiple navigation services allows us to consider different road network representations, routing criteria, and traffic data. These selected services cover a variety of routing criteria, from eco-friendly to the fastest and shortest routes, offering a comprehensive comparison. We evaluate the similarity among navigation services by calculating the average pairwise overlap among their recommended routes using the Jaccard Index. Figure S3 shows the average overlap (below the diagonal) and the percentage of exact matches (above the diagonal) of routes suggested by different navigation services for the same trips. Across the three cities, the average overlap between navigation strategies typically ranges from approximately 68% to 97%. Notably, the highest overlaps are observed among TomTom-based services, which rely on the same underlying road network representation and traffic data and differ primarily in their routing objectives. The exact matches between navigation services from different providers do not exceed 50%. We also include the SUMO fastest-path routing as an additional baseline for comparison. The overlap between SUMO-generated routes and those suggested by commercial navigation services is markedly lower than the similarity observed among navigation services themselves, ranging between approximately 52% and 63%. The exact matches between SUMO and navigation range from 28% to 39%. This indicates that SUMO's fastest-path routing differs systematically from routes suggested by commercial navigation services, reflecting differences in network representation, cost functions, and routing heuristics. A particularly high overlap is observed across all three cities between TomTom's eco routing (ecoTT) and shortest route (TTS) criteria, defined as trade-offs between travel time and fuel consumption for ecoTT, and travel time and travel distance for TTS, suggesting that these criteria lead to similar route recommendations. This indicates that routes optimised for fuel efficiency often align closely with those optimised for minimising travel distance, especially within the same road network and traffic data context. Overall, these observations underscore the variability and complexity in route recommendations provided by different navigation services despite some high overlaps among services from the same provider (e.g., TomTom).

Simulation of the combination scenario. Our primary study examined the influence of individual navigation services on urban traffic in isolation. However, as vehicles in real cities use a mix of these services, we extended our analysis to include a scenario where vehicles could choose among multiple services. We simulated scenarios in which each navigation service (GM, Bi, MB, TTF) had an equal market share, i.e., 25%, due to the lack of specific market share data for the services in the selected cities. Given an adoption rate of r, we randomly assigned $\frac{r}{4}$ of the vehicles

to each navigation service. This approach allowed us to create a more realistic model of how navigation services might interact in guiding traffic within a city.

Our findings from these extended simulations showed that the aggregate impact of using multiple navigation services was similar to the results observed when using single services in isolation. Specifically, the overall route diversity presents the same decreasing trend. However, the drop-off in route diversity at 100% adoption was less severe than single navigation services (see Figure S5). This is because the combination of multiple suggestions from various navigation services covered a larger extent of edges due to the difference in route service recommendations. The CO_2 emissions trends were consistent with the single-service scenario. In particular, the trend observed in the interplay of multiple navigation services resembled an average of the curves for individual services (see Figure S6).

Supplementary Note 3: Traffic Conditions

We employed a data-driven approach for each city to determine the number of vehicles N corresponding to high and low traffic conditions. Initially, we analysed the relationship between the number of teleports and the number of vehicles N using the fastest path (Figure S4). In this context, a "teleport" refers to a mechanism in SUMO that instantaneously relocates a vehicle stuck in traffic to maintain a smooth simulation flow and prevent deadlocks. It moves vehicles to a point where they can continue their trip. The number of teleports serves as a proxy for traffic congestion. Specifically, the number of teleports represents the average teleports observed in the simulation at a specific N for adoption rates ranging from 0% to 100%, incremented by 10%.

We identified the elbow point of this function, which indicates the N value at which the rate of increase in the number of teleports begins to rise sharply, signalling a significant change in congestion levels. This point serves as an ideal indicator for high traffic conditions. To define low traffic conditions, we selected an N value significantly lower than the elbow point. The specific N values for each city and traffic condition are defined as follows:

- **Florence**: N=10,000 for low traffic and N=30,000 for high traffic.
- **Milan**: N=30,000 for low traffic and N=65,000 for high traffic.
- **Rome**: N=25,000 for low traffic and N=75,000 for high traffic.

Supplementary Note 4: Route Diversity

In our study, we use route diversity to capture how routes are distributed across the road network. Route diversity is defined as the number of distinct road edges traversed by at least one vehicle. Formally, let R be a set of routes and e a road edge in these routes. Route diversity is defined as: $RD(R) = |\bigcup_{r \in R}\{e \in r\}|$. Higher values indicate a broader exploration of the network, whereas lower values reflect convergence onto a limited subset of edges.

Route diversity curve fitting. We investigate the relationship between the adoption rate and route diversity. We aim to identify the most appropriate curve model to represent these relationships. We consider several models: linear ($y = ax + b$), quadratic ($y = ax^2 + bx + c$), cubic ($y = ax^3 + bx^2 + cx + d$), and exponential ($y = a \cdot e^{b \cdot x} + c$). For each city, we calculate the average route diversity across different navigation systems at varying adoption rates and fit the data using these models. This analysis is performed under both low and high-traffic conditions. The goodness of fit for each model is evaluated using the R^2 metric. Our findings indicate that the exponential function consistently provides the best fit across all cities and traffic scenarios. Figure S7 illustrates the average route diversity alongside the best-fitting function.

Route diversity in treatment and control groups. To better understand the mechanism underlying this aggregate pattern, we analyse route diversity separately for treatment (s-routed) and control (non-s-routed) groups. For each city, we vary the adoption rate r from 0% to 100% in increments of 10% and compute route diversity separately for TTF-routed and non-routed vehicles as the number of vehicles in the treatment and control groups increases (Figure S8 a-f). In both groups, route diversity increases with the number of vehicles. However, this increase is consistently stronger for non-routed vehicles. The resulting differences are summarised in the box plots shown in Figure S8 g-i, which compare the distributions of normalised route diversity between routed and non-routed vehicles. In all cities, routed vehicles exhibit lower route diversity than non-routed vehicles. This indicates that vehicles following navigation service recommendations converge on a smaller subset of routes, resulting in increased route conformity.

Supplementary Note 5: Distribution of CO_2 Emissions Across Roads

Under certain conditions, navigation services can reduce or stabilise total CO_2 emissions as their adoption rate increases. However, as adoption continues to grow, emissions become increasingly concentrated on a smaller subset of roads due to stronger route conformity. This process amplifies inequalities in how emissions are distributed across the road network, even when aggregate emissions do not increase.

Inequality in the spatial distribution of CO_2 emissions. To quantify how navigation services affect the spatial concentration of CO_2 emissions, we examine the Gini coefficient of emissions across road segments as a function of the adoption rate. The Gini coefficient is a standard measure of inequality, ranging from 0, corresponding to perfect equality, to 1, indicating maximum inequality. Higher values reflect a more uneven distribution of emissions across the road network. Across Florence, Milan, and Rome, and under both low and high traffic conditions, the Gini coefficient increases monotonically with the adoption rate for all navigation services (Figure S9). This trend shows that navigation-induced route convergence systematically amplifies inequality in the spatial distribution of CO_2 emissions. To further illustrate this effect, we report Lorenz curves for each navigation service in Figure S10. These curves show the cumulative share of CO_2 emissions as a function of the cumulative share of road segments, ordered from least to most polluting. As adoption increases, the curves consistently deviate further from the line of equality, confirming that emissions become increasingly concentrated on a small fraction of the road network.

Distribution of emissions by road type. To investigate how navigation services redistribute traffic and emissions across urban road networks, we analyse the share of CO_2 emissions generated on each road type, as defined by OpenStreetMap classifications (e.g., motorway, primary, residential, etc.). For each navigation service, we compute the percentage of CO_2 emissions attributed to each road type at different adoption rates. This analysis is conducted separately for Florence, Milan, and Rome, with each value representing the average of 10 simulation runs per adoption rate. Figure S11 shows that, despite differences in their routing strategies, all navigation services and cities exhibit consistent trends in how emissions are redistributed across road types as adoption increases. These road-type-specific dynamics can be grouped into three general patterns:

- **Increasing Emission Share with Adoption: Motorway, Trunk, Primary**: Motorways exhibit the most pronounced and consistent increase in CO_2 emission share across all cities and navigation services, with particularly strong effects in Milan and Rome. Trunk roads show a milder and more variable increase, while primary roads generally display moderate growth or remain stable. These road types are preferentially selected by routing algorithms aiming to minimise travel time and avoid congestion, leading to a growing concentration of emissions on high-capacity, time-efficient corridors as adoption increases.
- **Decreasing Emission Share with Adoption: Residential, Tertiary, and Living Streets**: Residential and tertiary roads show a declining share of CO_2 emissions across all cities and navigation services as adoption increases, reflecting the tendency of routing algorithms to divert traffic away from lower-capacity local roads. Living streets and other low-priority road types contribute minimally overall, with emission shares remaining stable or decreasing further. Together, these patterns indicate that navigation services reduce traffic and emissions on neighbourhood streets under typical traffic conditions.
- **Stable or Mixed Behaviour: Secondary Roads and Link Classes**: Secondary roads display mixed patterns across cities and services. In some cases, such as Rome with GM or Bi, their emission share remains stable. In others, like Florence with MB, there is a slight decline. This variability likely reflects city-specific network structures and the nuanced differences in how each service prioritises mid-tier roads. Link roads (e.g., motorway_link, primary_link) generally show stable or mildly increasing emission shares, consistent with their role as connectors feeding traffic into higher-capacity roads.

In addition, we classify roads into two categories: high-capacity roads, defined as motorways and trunk roads that act as major corridors designed to efficiently accommodate large traffic volumes; and local roads, which comprise all remaining road types and primarily support local and distributive mobility. Figure S12 shows that the share of CO_2 emissions occurring on high-capacity roads increases steadily with adoption, indicating that navigation services progressively concentrate emissions, and thus environmental impact, onto a small subset of the road network. Although high-capacity roads account for only approximately 6% of the network, they absorb a disproportionate and growing share of total emissions. As adoption rises from 0% to 100%, the emission share on these infrastructures increases from 17.61% to 36.25% in Florence, from 9.94% to 26.42% in Milan, and from 26.02% to 33.66% in Rome.

Supplementary Note 6: Abstract Setting Experiments

We design an abstract setting to assess whether the key effects observed in our study (i.e., route conformity and diminishing returns in CO_2 reduction) can emerge independently of the structural complexity of real-world cities. Using abstract road networks with a uniform structure and synthetic mobility demand, we construct controlled environments that isolate the interplay between routing decisions and vehicle interactions. This approach allows us to determine whether navigation-induced traffic patterns are a general outcome of vehicle interaction and reduced route diversity rather than a byproduct of specific features of real cities.

a. Network Setup. We model the road network as a uniform 10×10 grid consisting of bidirectional, single-lane road segments, each 150 meters long and with a speed limit of 50 km/h. All intersections are four-way junctions, and U-turns are not permitted. This simplified topology eliminates variations in geometry, hierarchy, and connectivity found in real-world cities, allowing us to study the effects of navigation service adoption in a controlled environment.

This configuration ensures that all routes are comparable in structure and that differences in traffic dynamics emerge solely from routing strategies and vehicle interactions rather than topological asymmetries.

b. Routing Strategies. Vehicles are split into two groups: routed and non-routed.

- **Routed** vehicles follow the fastest path. We use the fastest path as a proxy for navigation services as it captures two key features of commercial systems: it selects routes based on an individually optimised criterion, specifically travel time, and it is deterministic, providing the same suggestion to all drivers, making the same request at the same time.
- **Non-routed** vehicles follow perturbed versions of the fastest path, introducing variability to reflect the imperfections of human route choices. These paths are computed using duarouter with a randomisation parameter $w \in \{3, 5, 7\}$, which scales the randomness applied to edge travel times.

c. Simulation Parameters. Each simulation is repeated ten times with different random seeds. In each run, we vary: The total number of vehicles N, ranging from 1,000 to 5,000 in steps of 1,000. The adoption rate r, defined as the share of routed vehicles, varies from 0% to 100% in increments of 10%. For each configuration, we measure route diversity and total CO_2 emissions.

d. Origin-Destination Configurations. We design two OD configurations to explore how vehicle interactions shape the impact of routing strategies.

- **Single OD flow**: In the baseline scenario, all vehicles travel from the western to the eastern edge of the grid. Origins and destinations are defined using relative coordinates $(0.2, 0.5)$ and $(0.8, 0.5)$, respectively. These positions are specified with respect to the network's bounding box and mapped to the closest nodes in the road graph to ensure feasible trip endpoints and to avoid boundary effects.
- **Two OD flows**: In this scenario, vehicles travel from two orthogonal flows. The first is the same east-to-west flow described above. The second is a north-to-south flow, defined by relative positions $(0.5, 0.8) \rightarrow (0.5, 0.2)$, also mapped to the closest network nodes. These flows intersect in the centre of the grid, creating a minimal condition in which vehicles share segments of the road network and can interact dynamically. As in the single-flow case, OD points are offset from the borders to mitigate boundary effects. By contrasting these two configurations, we assess whether route conformity and diminishing CO_2 reduction are structural outcomes of vehicle interaction or artefacts of complex urban environments.

e. Single OD Flow: Route Conformity Without Emission Inefficiencies. We start by simulating the single OD flow, where all vehicles travel in the same direction across the grid. As expected, route diversity declines markedly with increasing adoption: routed vehicles increasingly converge on the single fastest path (see Figure S13, top row). In this controlled setup, however, such route convergence does not translate into negative side effects. Since vehicle paths do not intersect and congestion is absent, the fastest route remains consistently optimal. As a result, CO_2 emissions decrease approximately linearly with adoption, reflecting the superior efficiency of routed paths compared to their perturbed alternatives (see Figure S13, bottom row). This trend holds across all traffic volumes (N), indicating that route conformity alone is not sufficient to generate inefficiencies. Instead, it is the interaction between competing vehicle flows that triggers congestion-driven effects and diminishing returns in CO_2 reduction, as observed in real cities.

f. Two Interacting OD Flows: Reproducing Emergent Patterns. To introduce interaction among vehicles, we simulate a configuration with two intersecting OD flows that share segments of the road network. In this configuration, we observe the emergence of all key patterns identified in real-city simulations. As the adoption rate increases, route diversity declines sharply as routed vehicles increasingly converge on overlapping paths, reducing the overall utilisation of the network (Figure S14, top row). Unlike in the single-flow scenario, this route alignment has system-wide consequences. Initially, total CO_2 emissions decrease due to more efficient routing. However, beyond a certain adoption rate, emissions plateau, particularly for higher vehicle volumes ($N \geq 3000$), as congestion on shared segments offsets the benefits of individual optimisation (Figure S14, bottom row).

We also examine the marginal relationship between changes in route diversity and changes in CO_2 emissions across adoption levels (Figure S15). The results reveal a nonlinear pattern. Early losses in route diversity are associated with substantial emission reductions. As adoption increases further, the marginal benefits diminish and eventually reverse.

In addition to reproducing route convergence and emission plateaus, the simplified grid also exhibits a marked increase in the inequality of CO_2 emissions as adoption rises. Lorenz curves and Gini coefficients confirm that emissions become increasingly concentrated on a smaller subset of road segments (see Figure S19).

These findings confirm that interaction among vehicle flows is a necessary condition for the emergence of navigation-induced inefficiencies. The simplified scenario with intersecting flows is sufficient to reproduce the full range of effects observed in more complex urban systems.

g. Generalisation to Larger and Randomised Networks. To assess the robustness of the observed dynamics beyond the 10×10 grid, we extend our experiments to larger and structurally irregular networks. We first replicate the two-flow configuration on a 14×14 uniform grid. The results remain consistent: increasing adoption leads to reduced route diversity and a plateau in total CO_2 emissions (Figure S16). These trends hold across all traffic volumes and match those observed in real-world urban simulations. This confirms that the mechanism identified in the simplified model scales with network size and is not an artefact of small spatial domains.

Next, we test the generality of the effects using randomised grid-like networks. We generate 20 small and 20 large randomised networks using SUMO's Netedit tool. Each network is constructed to have an edge count approximately equal to its regular grid counterpart to ensure comparable structural complexity. To enable comparisons across different network topologies, we normalise both route diversity and total CO_2 emissions. The results for small (Figure S17) and large networks (Figure S18) confirm that the key effects persist in the vast majority of randomised networks: route diversity consistently declines with adoption, and CO_2 emissions plateau at higher adoption levels. The red line shows the average across instances, while the opaque lines represent individual randomised grids. Only a few networks (typically 3 or 4 out of 20) deviate from this pattern, often due to structural constraints such as fragmented subregions or narrow bottlenecks that reduce route choice flexibility.

We do not report the marginal diversity-emission trends for the randomised grids, as these results are aggregated across multiple heterogeneous network instances. Computing ΔD_r and ΔE_r across such structurally diverse topologies would require per-instance marginal analysis, which is not directly comparable due to differences in edge configurations and congestion patterns. Instead, we rely on normalised aggregate trends in route diversity and total CO_2 emissions, which clearly reproduce the key effects.

This increase in emission inequality also persists in randomised grid-like networks. As shown in Figure S19, Gini coefficients rise consistently with adoption, indicating that navigation-induced concentration of emissions is robust to topological irregularity.

Overall, these experiments demonstrate that the observed dynamics are not artefacts of specific network designs, city features, or simulation parameters. Instead, they emerge from fundamental coordination effects and are likely to generalise broadly across a wide range of mobility systems.

Supplementary Note 7: Sensitivity analysis on the duarouter parameter w

In our study, we set $w = 5$, introducing a moderate level of randomness into the computation of the fastest path using duarouter. To evaluate the robustness of our findings with respect to the routing model for non-routed vehicles, we performed a sensitivity analysis on the duarouter randomisation parameter w. In addition to our default setting of $w = 5$, we conducted simulations using $w = 3$ and $w = 7$, which represent lower and higher levels of route variability, respectively. These values were selected to span a plausible range of randomness while preserving realistic trip behaviour for non-routed vehicles. The results, presented in Figure S20, show that the key patterns are consistently preserved across all tested values of w: the curves for both route diversity and CO_2 emissions maintain the same overall shapes and trends across cities and adoption rates of navigation services. While the absolute values of the metrics shift slightly with w, the qualitative behaviour remains stable. As expected, larger values of w lead to increased route diversity, as higher randomisation naturally results in greater dispersion in route selection. Conversely, higher w values are generally associated with slightly elevated CO_2 emissions per vehicle due to longer and less efficient routes introduced by more aggressive edge weight perturbation. These findings confirm that our findings remain robust to moderate changes in the routing variability modelled by duarouter.

References

[1] Pappalardo, L., Simini, F., Barlacchi, G. & Pellungrini, R. scikit-mobility: A python library for the analysis, generation, and risk assessment of mobility data. *Journal of Statistical Software* **103**, 1–38 (2022).

[2] Zheng, Y. Trajectory data mining: An overview. *ACM Trans. Intell. Syst. Technol.* **6** (2015).

[3] Zhu, L., Holden, J. R. & Gonder, J. D. Trajectory segmentation map-matching approach for large-scale, high-resolution gps data. *Transportation Research Record* **2645**, 67–75 (2017).
