# Supplementary material for: The traffic concentration effects of urban navigation services
Source: Nat Commun. 2026 Jul 11;17:8548. doi: 10.1038/s41467-026-75254-8 (PMC13482866; doi:10.1038/s41467-026-75254-8)
Supplement: Supplementary file 2 [file 41467_2026_75254_MOESM2_ESM.pdf]

nature portfolio

Peer Review File

The traffic concentration effects of urban navigation services

Corresponding Author: Dr Luca Pappalardo

This file contains all reviewer reports in order by version, followed by all author rebuttals in order by version.

Version 0:

Reviewer comments:

Reviewer #1

(Remarks to the Author)
The paper describes results from an assessment of routing services provided by several commercial providers. It found, that the route diversity decreases for a large share of route users, thereby concentrating traffic on few edges of the network. While not totally surprising, the quantification done in this paper is useful, therefore I think this paper is publishable. However, I have a lot of concerns about the paper which could be found below, which marks it as major revision.

General concern: the route recommendations of the various providers may depend on r; what is even worse, in congested cases I have seen them oscillating between several possible best routes; so, the assumption that they all use the same route(s) for r \to 1 is very questionable. Please discuss.

And: some of the material covered here is well-known to transportation engineers, so I am not sure what to do with this. I have indicated this in my review below.

What also annoys me are the too many cross-references to the supplementary material. Try to reduce this considerably, in my view 11 SM and 31 Figures in the SM is way too much. I know, there are length-limitations, but at some point the text is no longer self-referential, since it needs the SMs. Which is no longer "supplementary".

"The collective impact of GPS navigation services is unclear: while beneficial to the user"
>> (i) Please use GNSS instead of GPS. GPS is only the USA GNSS. (ii) In uncongested networks, the beneficial is true. In congested networks, not so. Please formulate more carefully. Later on, this is in fact discussed, so a "they may be beneficial..." might be the better formulation.

"increased concentration of traffic and emissions on fewer roads, thus exacerbating an unequal distribution of negative externalities on selected neighbourhoods."
>> Again, more careful formulation, please. It may also happen, that the services recommend to use major roads and to actually relieve neighbourhoods with small roads, since it is not efficient to drive there. And this is what you actually observe.

I am unhappy with the first paragraph of the introduction: while this is all true, it has not much connection to your topic; I do not see that navigation services are a human-AI ecosystem. Human, yes, but AI? Nope, Dijkstra's algorithm is from the 1950ies, there is little AI here. Consider a re-formulation, or even removing.
And please: this kind of interaction is also long known in the transport planning community, starting (but not ending) with the work of Wardrop (see below); more details can be found in Sheffi's book, which is a little outdated, but still a kind of reference in this area: https://sheffi.mit.edu/sites/sheffi.mit.edu/files/sheffi_urban_trans_networks_0.pdf

Also, please state that the Leonia and Brenner stories are in the form told here, are anecdotically. I am more than sure, that there are many more occurances like these, most likely with smaller effects so that they do not make it into the headlines. Nevertheless, it is truly difficult to actually fix and quantify this in real life. Furthermore, one has to weigh it against the positive features when those systems direct people to the fastest path which would otherwise run much longer pathes. And this happens during all non-peak times, which are typically more common than the peak times (but carry less traffic, sure).

--> So far, your paper is a bit on the alarming side, which I do not like and which borders on journalistic. Consider a more neutral wording. And more of your wording is a bit too advertising ("unprecedented view" -- wow, "foundational step" for what is being done in the traffic engineers communities for ages without thinking too much about it since it is fairly trivial, see the

remark about Sheffi above, and Wardrop below), please tone down a bit and stick to a neutral, scientific language.

Once the penetration rate exceeds a service-specific threshold, these benefits diminish, disappear, or even reverse.
>> This depends on the type of service actually offered, and I think for current navigation services it is true enough. However, and we have discussed this with colleagues from TomTom already 15 years ago, if the service does not simply offer a fastest path but in addition before doing so computes a (dynamic, stochastic) user equilibrium (see Sheffi, sorry for re-iterating), then such a service will lead to an optimal utilization of the network. Clearly, doing this is way too expensive for current navigation service providers, but in theoretical work as the one here, it should at least not go unmentioned.

For your OD matrix: I think it is critically important that it is time-dependent, i.e. m_{o,d}(t) or m_{o,d,t}. Have you done this? (Later on I have seen that you have not done it; but traffic does not depend only of peak hours, but of a continuum of demand between very small and very large.)

Have you checked the overlap of SUMO's routes with the ones of the various providers?

"We assume that vehicles in the control group select their routes independently of one another"
>> But this is exactly what SUMO's duarouter is for. Which is essentially Wardrop's first principle. (https://en.wikipedia.org/wiki/John_Glen_Wardrop#Wardrop_equilibria) And of course, Sheffi.

"route diversity varies considerably with r following an exponential function"
>> I have not checked SM 7, but 2a-c do not look exponential. Maybe you confused this with the \Delta E vs \Delta D? And: the change in route diversity is roughly a 10% effect, which I think is not that dramatic.

"Across all cities, we observe a consistent shift of routes, and consequently CO2 emissions, toward high-capacity infrastructures such as highways and major arterial corridors (attractors)."
>> As already mentioned: I cannot see anything bad here, since these roads are made for exactly this purpose. Should they route all this traffic through residential roads? What is your idea here? Furthermore, as is done in transport planing, one usually tries to estimate the immissions, und with this, target also how many people are actually affected. For this, however, one must have additional information about the population, their vulnerability to the various immissions (CO2 and traffic load just one of them), which makes good transportation planing so complicate.

"Milan has the highest coefficient value (beta = 0.0068), indicating that changes in route diversity have a more significant impact on CO2 reduction compared to Florence (beta = 0.00313) and Rome (beta = 0.00226)"
>> Do you have error measures and statistical significances for these parameters? Are the three beta different (I would assume this, but better safe than sorry)?

I am not exactly sure, why Fig. 4 is needed. It has nothing new over Fig. 3. Please discuss.

Minor:
======

It might be picky, but I would prefer the correct writing of chemical formulas like CO2 and NOx with indexed numbers.

Fig. 3 caption: it says 3d-f are the Gini coefficients, but the y-axis description does not reflect this. Please adopt.

Please fix Refs 26, 41, 49, and maybe others which I overlooked. At least an URL or DOI would be very welcomed.

(Remarks on code availability)

Reviewer #2

(Remarks to the Author)
Indeed, the order of presentation in the manuscript is problematic and undermines its coherence and scientific communication quality. In high-impact journals the structure of a paper is not merely a stylistic matter but a reflection of logical flow, reproducibility, and argumentative strength.
1. Placement of the “Methods” section
Observation: The Methods section appears near the end of the manuscript, after the results and discussion. Although this format is acceptable in some journals, where methodological details are placed after the main text to emphasize narrative flow, it is not appropriate for technically oriented research, especially in computational simulation studies. Readers need to understand the experimental setup before interpreting the results.
Suggestion:

1. Abstract
2. Introduction
3. Methods
4. Results and Discussion
5. Conclusions
6. References

This order preserves a natural progression from conceptual background to empirical validation and discussion.

2. Absence of conclusions: The manuscript lacks a conclusion section, which represents a significant structural flaw. Without it, the paper feels unfinished and lacks a synthesis of its theoretical, methodological, and practical contributions. Moreover, most Scopus- or WoS-indexed journals explicitly require a “Conclusions” section for indexing and thematic classification.
3. Placement of figures and tables: All figures and tables appear at the end of the manuscript. While this layout is acceptable for preprint submissions, it interrupts the visual continuity between text and evidence. Figures such as 1 (simulation flow) and 5 (concentration patterns) are key for understanding the methodology and should be embedded where first discussed. Integrate figures and tables within the main text or include reduced versions (thumbnails) in-line, referring to the full versions in the appendix. This will improve reader comprehension and facilitate peer review.

Title
The title of the manuscript, “Algorithmic navigation reduces route diversity and amplifies emissions inequality in cities,” is appropriate in terms of scientific precision and conceptual clarity, as it accurately summarizes the study’s main finding: the negative impact of algorithmic navigation on route diversity and the unequal distribution of urban emissions. However, from an editorial perspective, the title could be strengthened by explicitly referencing the methodological approach employed (simulation based on real-world data) and the type of emissions analyzed (CO_2), which would broaden its scope and enhance the identification of its application field. Suggestions such as “Modeling the Urban Impact of Algorithmic Navigation: Reduced Route Diversity and Unequal CO_2 Emissions” or “Algorithmic Navigation Concentrates Urban Traffic and CO_2 Emissions, Reducing Route Diversity in Cities” would add greater specificity and appeal for interdisciplinary audiences in mobility, artificial intelligence, and urban sustainability, without losing the scientific rigor of the original title.

Abstract
The abstract of the article presents a coherent and academically solid structure, introducing the study’s context, methodology, results, and implications. However, several aspects could be improved to meet the scientific writing standards expected in journals, particularly regarding methodological precision, quantification of results, and connection with practical and regulatory implications. Below are the specific observations and suggestions organized by content blocks.
Lines 1–5:
Observation: The opening effectively presents the study’s context, but the introductory sentence is too general and does not immediately define the central scientific problem.
Suggestion: Rephrase the first couple of sentences to establish from the beginning the relationship between algorithmic navigation and its environmental impact, for example: “Algorithmic navigation systems, widely used in urban mobility, can unintentionally reshape traffic flows and amplify emission inequalities.”
Lines 6–12:
Observation: The abstract mentions that the study employs a simulation-based framework using real-world data, but it does not specify the nature or origin of the data.
Suggestion: Include a brief reference to the source or type of data used (e.g., GPS data, urban traffic records, or mobility datasets), which would enhance transparency and reproducibility.
Lines 13–18:
Observation: The results are presented generally, indicating that navigation services reduce emissions at low adoption rates but increase them at higher ones. However, no quantitative effects are provided.
Suggestion: Add approximate values or variation ranges (e.g., percentage reduction or increase in CO_2) to strengthen the empirical rigor and impact of the summary.
Lines 19–24:
Observation: The description of the nonlinear function relating route diversity and emissions is interesting but introduced abruptly, without a clear connection to the rest of the text.
Suggestion: Include a short transition phrase to contextualize its relevance, for example: “To mathematically capture this dynamic, we describe the relationship between route diversity and CO_2 emissions through an exponential function.”
Lines 25–30:
Observation: The closing paragraph mentions the applicability of the proposed framework and the regulatory relevance of the study but does not elaborate on its broader implications or specific policies it may inform.
Suggestion: Add a sentence highlighting the implications for urban planning or digital platform regulation, for example: “These findings may inform urban policy and digital platform governance, especially under frameworks such as the EU Digital Services Act.”
Lines 31–33:
Observation: The abstract ends abruptly, lacking a final sentence that reinforces the scientific contribution or added value of the work.
Suggestion: Conclude with a statement emphasizing the novelty and interdisciplinary relevance of the study, for example: “This work offers a novel perspective on how AI-driven mobility systems can shape environmental inequalities in cities.”

Introduction
The introduction of the article progressively presents the context of human–AI ecosystems and their side effects, establishing a parallel between different types of algorithms—such as recommendation systems or digital navigation—and their social impacts. It then focuses on commercial navigation services, providing real-world examples of urban congestion caused by the widespread use of these platforms. The authors subsequently identify the existing scientific gap regarding a comprehensive analysis of their influence on traffic and emissions and describe the methodological framework proposed to address this issue. Finally, the introduction anticipates the main results and the regulatory relevance of the study, linking them to policies such as the European Union’s Digital Services Act.
Lines 19–28:

Observation: The transition between the examples of human–AI interaction and the topic of vehicular navigation is not entirely clear.
Suggestion: Add a linking sentence that explicitly connects the effects of recommendation systems with those of algorithmic navigation, for example: “A similar pattern may emerge in algorithmic systems shaping urban mobility.”
Lines 29–40:
Observation: The narrative tone of the Leonia and Italian region cases is more journalistic than academic.
Suggestion: Rephrase the descriptions in a scientific style, avoiding colloquial expressions, for example: “residents experienced severe accessibility restrictions due to algorithm-induced congestion.”
Lines 41–57:
Observation: The research question is expressed rhetorically, and the paragraph contains overly long sentences.
Suggestion: Convert the question into a declarative statement and split long sentences for better readability, for example: “This study aims to quantify the impact of navigation service adoption rates on urban traffic and CO_2 emissions.”
Lines 58–65:
Observation: There is a lack of contextual information regarding the cities or datasets used in the simulation framework.
Suggestion: Include a brief mention of the data source or characteristics (scale, region, mobility pattern) to enhance reproducibility.
Lines 66–84:
Observation: This section mixes results and analysis that should be reserved for later sections.
Suggestion: Summarize the main ideas and move the detailed discussion of non-linear behavior and the exponential function to the results section.
Lines 85–91:
Observation: The conclusion limits the study’s applicability to the European context.
Suggestion: Add a brief statement on the potential applicability of the methodological framework to other regions to broaden the study’s global scope.
Simulation Framework
The “Simulation Framework” section provides a detailed methodological structure used to model the impact of navigation services on urban traffic and emissions. The text follows a logical sequence: it begins by defining the simulation environment (SUMO), explains the representation of the road network and the generation of mobility demand, details the integration with commercial navigation service APIs, and concludes with the experimental design, evaluated metrics, and methodological limitations.

Lines 93–98:
Observation: The introduction of the SUMO tool and the justification for its use are clear, but there is no brief reference to its validation or accuracy in previous studies.
Suggestion: Add a sentence supporting the choice of SUMO by citing recent literature on its reliability as a traffic simulation tool, for example: “SUMO has been validated in several large-scale traffic studies for its accuracy in replicating real-world urban conditions (Behrisch et al., 2019).”

Lines 99–115:
Observation: The explanation of the construction of the road graph and OD matrix is precise but somewhat lengthy and too technical for a main section.
Suggestion: Condense the mathematical description of the process and refer readers to the Methods or Supplementary Material for details. Additionally, briefly explain why “square tiles” were chosen instead of other spatial divisions (e.g., census zones or districts) to justify spatial representativeness.

Lines 116–126:
Observation: The description of API-based navigation services is thorough, but the justification for forcing the use of historical information (instead of real-time traffic data) could be more explicit regarding experimental validity.
Suggestion: Add a brief methodological rationale explaining how this choice avoids bias or ensures comparability, for example: “Using historical traffic conditions ensures experimental consistency and eliminates noise from exogenous real-time events.”

Lines 127–135:
Observation: The navigation services used are listed, but the selection criteria are not explained, nor is there clarification on how differences among algorithms (e.g., optimization heuristics) are handled.
Suggestion: Include a sentence justifying the selection of these services (due to commercial relevance or API accessibility) and acknowledge the “black-box” limitation of the algorithms, emphasizing how this may influence result variability.

Lines 136–149:
Observation: The experimental design with treatment and control groups is well described, but the modification of the driving model (to simulate human imperfections) is too briefly discussed.
Suggestion: Expand slightly on the explanation of the “fastest route” modification and its basis in driver behavior literature, or move these details to the Methods section for greater clarity.

Lines 150–157:
Observation: The choice of metrics (route diversity and CO_2 emissions) is appropriate but lacks justification for excluding other relevant variables such as energy consumption or average travel time.
Suggestion: Add a brief justification for focusing on these metrics, emphasizing their environmental relevance and connection to the study’s central hypothesis.

Lines 158–167:
Observation: The limitations section correctly addresses the issue of group interference, but the reference to the Stable Unit Treatment Value Assumption (SUTVA) may be too technical for non-specialist readers.
Suggestion: Complement this with a more accessible explanation of the practical implications, for example: “Because vehicles influence each other’s movements, the separation between treatment and control groups is imperfect, potentially diluting observable effects.” It would also be useful to include a note on the expected magnitude of this bias or any statistical controls used.

Impact of Navigation Services
This section presents the experimental results and analyzes how the progressive adoption of algorithmic navigation services affects route diversity and CO_2 emissions in three Italian cities. The structure is clear and logically sequenced: it begins with the selection of cities, then discusses results on route diversity, total emissions, the spatial distribution of emissions, optimal adoption patterns, and finally the quantitative relationship between route diversity and emissions.

Lines 169–175:
Observation: The selection of cities is well justified by their heterogeneity, but the explanation is more descriptive than analytical.
Suggestion: Add a brief scientific justification explaining how morphological and structural differences among the cities help validate the generalizability of the results, for example: “These cities represent diverse topologies that enable assessing the robustness of algorithmic navigation effects under different urban morphologies.”

Lines 176–190:
Observation: The description of results regarding route diversity is clear, but the text includes too many numerical values that disrupt the flow of reading.
Suggestion: Retain only the most representative figures and move secondary percentages to the supplementary material. Also, summarize the overall behavior in an interpretive sentence, for example: “Route diversity initially increases at low adoption levels but sharply declines once the adoption threshold is surpassed.”

Lines 191–197:
Observation: The interpretation of traffic concentration is correctly framed but somewhat repetitive compared with the methodology section.
Suggestion: Restructure the paragraph to emphasize the causal mechanism (algorithmic route homogenization) and its link to the OD matrix structure, avoiding repetition of previously discussed explanations.

Lines 198–204:
Observation: The emissions results are presented precisely, but the narrative lacks comparative insight across cities and navigation services.
Suggestion: Introduce a short comparative analysis summarizing key differences, for example: “The emission-reduction patterns are more pronounced in smaller cities like Florence and less consistent in larger urban areas such as Rome.”

Lines 205–221:
Observation: The discussion on emission inequality is compelling, but it overuses visual descriptions (figure references) without interpreting their urban or environmental implications.
Suggestion: Complement the analysis with a short interpretation of the environmental and social meaning of this concentration, for example: “This spatial concentration of pollutants may exacerbate localized air quality degradation and social exposure disparities.”

Lines 222–232:
Observation: The analysis of “attractors” is relevant, but the term may be ambiguous for readers from other disciplines.
Suggestion: Explicitly define what is meant by “attractors” (e.g., “high-capacity road segments that algorithmically attract flows”) and add a reflection on the conflict between algorithmic efficiency and environmental sustainability.

Lines 233–243:
Observation: The text includes numerous numerical values and adoption thresholds but lacks a synthesis of general implications.
Suggestion: Add a closing statement summarizing the key finding, for example: “These results indicate that there exists an optimal adoption range—typically between 60% and 80%—beyond which the environmental benefits of navigation services decline.”

Lines 244–247:
Observation: The argument regarding the limitations of eco-routing is conceptually strong but phrased too simplistically.
Suggestion: Reinforce the theoretical idea by stating that sustainability depends not on individual routes but on the collective distribution of algorithmic decisions, which broadens the relevance of the finding.

Lines 248–269:
Observation: The description of the correlation between route diversity (ΔDr) and emissions (ΔEr) is solid but overly technical and dense with formulas.
Suggestion: Simplify the mathematical explanation by emphasizing the conceptual interpretation: “A strong negative

correlation between route diversity and CO_2 emissions indicates that as traffic becomes more concentrated, marginal emission reductions diminish." Also, move the formal equation to the Methods section or Supplementary Material.
Lines 270–278:
Observation: The section opens with a clear rationale, emphasizing the need to isolate the fundamental mechanisms of traffic concentration. However, the explanation of the experimental simplification is overly brief.
Suggestion: Expand on why a grid-based network was chosen as a simplification, for example: "The grid structure allows controlling network topology while preserving key flow interaction properties." This would strengthen the methodological justification.

Lines 279–284:
Observation: The description of the first experiment (single OD flow) is precise but lacks interpretive depth. The authors mention that route diversity decreases while emissions also decline but do not analyze why.
Suggestion: Include a short interpretation linking this to the absence of vehicle interactions or congestion, such as: "This suggests that in the absence of network interactions, concentration effects remain benign and do not generate emission inefficiencies."

Lines 285–292:
Observation: The second experiment (two intersecting OD flows) effectively reproduces real-world congestion effects, yet the text relies heavily on figure references instead of analytical commentary.
Suggestion: Add a more conceptual discussion highlighting how the intersection of flows introduces systemic interdependence, transforming a purely geometrical setup into a dynamic analogue of urban congestion.

Lines 293–296:
Observation: The robustness verification through larger and randomized networks is appropriate but superficially discussed.
Suggestion: Briefly mention how network regularity and randomness affect the persistence of the observed effects to show that the findings are not limited to grid symmetry. For instance: "This confirms that the emergence of concentration patterns is a structural property of algorithmic routing, not a by-product of network regularity."

Lines 297–299:
Observation: The concluding statement effectively generalizes the findings but could be more assertive regarding their implications.
Suggestion: Reinforce the conclusion by linking it to the broader debate on algorithmic urbanism, e.g., "These results indicate that algorithm-driven concentration is a universal phenomenon likely to manifest across diverse urban morphologies, highlighting the need for regulatory attention."
Discussion
This section effectively connects the empirical findings with their theoretical, social, and policy implications. The narrative is well-structured, moving from the summary of results to reflections on urban inequality, the extension of the model to other mobility platforms, and finally to the human–AI interaction and regulatory implications. However, while the content is solid, the writing could benefit from stronger conceptual integration and deeper argumentation on the normative and ethical implications of algorithmic mobility systems.
Lines 301–305:
Observation: The opening paragraph summarizes the main finding (traffic and emissions concentration) clearly but does not explain how this concentration emerges as a systemic phenomenon beyond the observed empirical patterns.
Suggestion: Add a sentence that frames the phenomenon as an emergent property of socio-technical systems, for example: "This pattern can be interpreted as an emergent property of algorithmic coordination within decentralized mobility systems."
Lines 307–315:
Observation: The discussion on urban inequality is pertinent, but it focuses mainly on material consequences (congestion, pollution, property values) without addressing deeper notions of environmental or urban justice.
Suggestion: Strengthen this argument by connecting it with the theoretical framework of environmental justice (Harvey, 1996; Schlosberg, 2007), emphasizing that traffic concentration not only produces economic dysfunction but also environmental exclusion and unequal exposure to risk.
Lines 316–322:
Observation: Extending the model to shared mobility platforms is a conceptual strength, but the text does not clearly distinguish between algorithmic recommendation mechanisms and those of matching or dispatching (as in ride-hailing).
Suggestion: Clarify how the framework can adapt to different algorithmic decision models (e.g., dynamic matching vs. deterministic routing) and how these distinctions influence the simulation of environmental impacts.
Lines 323–330:
Observation: The discussion on human–AI coevolution is theoretically relevant but remains somewhat superficial: the "feedback loop" is mentioned without exploring its temporal dimension or implications for adaptive algorithmic learning.
Suggestion: Reinforce this point by referencing theories of algorithmic feedback loops (Rahwan et al., 2019; Epstein et al., 2020) or adaptive socio-technical systems, explaining how algorithmic recommendations create behavioral dependencies that perpetuate congestion patterns.
Lines 331–342:
Observation: The closing discussion on replicability and public policy is strong but written in a mostly descriptive tone. It lacks a critical reflection on how existing legislation (Digital Services Act and Digital Markets Act) could materially affect the transparency and governance of navigation algorithms.
Suggestion: Add a statement discussing the limitations of current regulation, for example: "However, transparency mandates alone may not suffice without enforceable accountability mechanisms to ensure that algorithmic behavior aligns with public policy objectives."

Methods
Lines 344–354: Road Networks
Observation: The description of the road network modeled as a directed weighted multigraph is accurate, but the mathematical formalism may be excessive for readers of interdisciplinary journals. Additionally, the validation using Google Maps and StreetView is mentioned, but the extent of the corrections is not quantified.
Suggestion: Simplify the formal notation, relegating mathematical details to the supplementary materials. Including an estimate of the percentage of manually corrected edges (e.g., "approximately 3–5% of edges required manual adjustments") would enhance methodological transparency.

Lines 355–367: Traffic Simulator (SUMO)
Observation: The description of SUMO and its use of the HBEFA3 emission model is precise, but the emission equation lacks context regarding units and coefficient interpretation.
Suggestion: Add a brief note explaining how the parameters c0,…,c5c_0, \ldots, c_5c0,…,c5 correspond to different vehicle types (e.g., diesel vs. gasoline) and how these distinctions are integrated into the model. This would strengthen the connection between simulation output and real-world emission behavior.

Lines 368–384: Navigation Services APIs
Observation: The selection of navigation services is well justified in terms of availability and diversity, but the comparison methodology among them could be more clearly explained.
Suggestion: Specify whether route requests were made under identical temporal and geographic conditions for all services and how discrepancies in output formats were handled. It would also be advisable to briefly discuss the potential accessibility bias introduced by using only public APIs (since services such as Waze or Apple Maps were excluded).

Lines 385–401: Modified Fastest Route (Control Group)
Observation: The introduction of the randomness parameter www in the duarouter algorithm is an interesting methodological contribution, but the explanation might be difficult for non-specialist readers.
Suggestion: Include a short practical interpretation, such as: "This parameter simulates the stochastic nature of human decision-making in routing, such as preferences for familiar paths or avoidance of complex intersections." Also clarify whether the chosen value w=5w = 5w=5 was based on empirical calibration or adopted from previous studies.

Lines 402–438: GPS Data and OD Matrix Construction
Observation: The description of the GPS dataset is comprehensive and one of the study's major strengths. However, this section could benefit from a more critical discussion about representativeness limitations (only 2% market penetration).
Suggestion: Add a methodological reflection on sampling bias and explain how it was mitigated (e.g., through OD matrix weighting or validation against traffic counts). It would also be valuable to justify the choice of Wednesday as a reference day, supported by a methodological citation from transportation research.

(Remarks on code availability)
The code is reproducible and provides a valuable resource for the research community. The repository includes a well-documented README file with clear installation instructions, example commands, and configuration details, making it easy to reproduce the simulations. The code is well structured, modular, and allows replication of the main workflow from data preprocessing to SUMO-based simulation. Although proprietary datasets are not included, the authors provide public alternatives that ensure functional reproducibility. Overall, the code meets high standards of transparency, usability, and methodological rigor.

Version 1:

Reviewer comments:

Reviewer #1

(Remarks to the Author)
This is the revised review: the authors have paid heed to all of my points, therefore the paper is fine with me and can be published.

(Remarks on code availability)
None

Reviewer #2

(Remarks to the Author)
I have carefully reviewed the revised version of the manuscript as well as the detailed responses provided to the reviewers. I appreciate the authors' efforts in addressing the comments and improving the clarity and structure of the paper.
Overall, the authors have satisfactorily implemented the majority of the suggested revisions. The methodological explanations, the clarification of the emission model, the justification of the navigation services used, the discussion of the GPS data representativeness, and the restructuring of the discussion section significantly improve the scientific clarity and

transparency of the manuscript. The revisions have strengthened the methodological description and the interpretation of the results.
However, one aspect still requires further improvement. While the authors have attempted to address the comment regarding the comparison between cities, the discussion remains relatively limited and largely descriptive. Given that the study includes multiple cities with potentially different urban morphologies and traffic characteristics, a slightly deeper comparative discussion would strengthen the contribution of the paper. Expanding this section to better highlight how and why the observed effects differ (or remain consistent) across the cities analyzed would improve the generalizability and interpretation of the results.
I therefore recommend that the authors further refine and expand the inter-city comparison in the results or discussion section. Aside from this point, the manuscript has satisfactorily addressed the reviewer's previous comments.

(Remarks on code availability)
Yes. The code provided by the authors is adequately documented and appears to be a usable resource for the community. The repository includes a README file with clear instructions for installation and execution. Based on the information provided, the workflow and methodology are sufficiently described to allow the reproduction of the main results reported in the manuscript. Overall, the code contributes positively to the transparency and reproducibility of the research.

Open Access This Peer Review File is licensed under a Creative Commons Attribution 4.0 International License, which permits use, sharing, adaptation, distribution and reproduction in any medium or format, as long as you give appropriate credit to the original author(s) and the source, provide a link to the Creative Commons license, and indicate if changes were made.
In cases where reviewers are anonymous, credit should be given to 'Anonymous Referee' and the source.
The images or other third party material in this Peer Review File are included in the article's Creative Commons license, unless indicated otherwise in a credit line to the material. If material is not included in the article's Creative Commons license and your intended use is not permitted by statutory regulation or exceeds the permitted use, you will need to obtain permission directly from the copyright holder.
To view a copy of this license, visit https://creativecommons.org/licenses/by/4.0/

Reviewer #1

We thank the Reviewer for the careful reading of our manuscript and for the valuable suggestions provided. We believe that these comments have substantially improved the quality and clarity of the paper.

Point 1.1

General concern: *the route recommendations of the various providers may depend on r; what is even worse, in congested cases I have seen them oscillating between several possible best routes; so, the assumption that they all use the same route(s) for r \to 1 is very questionable. Please discuss.*

Answer 1.1

We agree with the Reviewer that the routes suggested by navigation systems may vary depending on the level of congestion in the city. In this study, we focus on a high-congestion scenario corresponding to a morning peak hour. The choice of the morning peak period is data-driven: using GPS data, we identify the one-hour morning interval with the highest traffic demand, defined as the largest number of trip departures. Based on this procedure, the morning peak hours are 8-9 for Florence, 7-8 for Milan, and 8-9 for Rome.

We clarify this in Section "Impact of Navigation Services":

> "We simulate traffic in each city during the morning peak, defined from vehicular GPS data as the one-hour morning slot with the highest traffic intensity."

and add the discussion above to Supplementary Note 1.

Moreover, in the Discussion we acknowledge that commercial navigation services may suggest more than one routing option for a given query. However, this does not eliminate the concentration effect: at any given time, services typically offer only a limited set of routes, which leads to a persistent reduction in route diversity through the algorithmic funneling of traffic toward a small number of corridors. This interpretation is supported by our experiments, which show that the effect persists even when drivers can choose among multiple routes offered by different services rather than following a single recommendation (**see Supplementary Note 2**).

Point 1.2

Some of the material covered here is well-known to transportation engineers, so I am not sure what to do with this. I have indicated this in my review below.

Answer 1.2

Thank you for this comment, we have addressed all these concerns in the answers to the points below.

Point 1.3

What also annoys me are the too many cross-references to the supplementary material. Try to reduce this considerably, in my view 11 SM and 31 Figures in the SM is way too much. I know, there are length-limitations, but at some point the text is no longer self-referential, since it needs the SMs. Which is no longer "supplementary".

Answer 1.3

We thank the Reviewer for this comment, which prompted us to substantially streamline the Supplementary Material and reduce reliance on cross-references. Following the revisions described below, the Supplementary Material has been significantly condensed and reorganized. The revised version now includes seven Supplementary Notes and 20 Supplementary Figures, compared to 11 Supplementary Notes and 31 figures in the previous version.

We moved core methodological elements (including impact measures and essential simulation details) from the Supplementary Material into the main text, removed redundant or overly technical material, and consolidated closely related Supplementary Notes. We also reduced and merged several Supplementary Figures by aggregating related visualizations and eliminating redundancies.

Point 1.4

"The collective impact of GPS navigation services is unclear: while beneficial to the user" >>

1. *(i) Please use GNSS instead of GPS. GPS is only the USA GNSS.*
2. *(ii) In uncongested networks, the beneficial is true. In congested networks, not so. Please formulate more carefully. Later on, this is in fact discussed, so a "they may be beneficial..." might be the better formulation.*

Answer 1.4

We thank the Reviewer for these clarifications. We agree that GNSS is the technically correct term, as GPS refers specifically to the U.S. system. To avoid ambiguity and ensure terminological accuracy, we now use the more general expression "navigation services" throughout the manuscript.

We also agree that the term "beneficial" in the original abstract could be misleading. Our intention was to convey that such services are useful at the individual level, rather than universally beneficial. To remove any ambiguity, we rephrase the first sentence of the abstract as follows:

> "The collective impact of navigation services is unclear: while often beneficial to individual drivers, they can also unintentionally reshape traffic patterns in urban spaces."

Point 1.5

"increased concentration of traffic and emissions on fewer roads, thus exacerbating an unequal distribution of negative externalities on selected neighbourhoods." >>
Again, more careful formulation, please. It may also happen, that the services recommend to use major roads and to actually relieve neighbourhoods with small roads, since it is not efficient to drive there. And this is what you actually observe.

Answer 1.5

Thank you. To avoid misunderstanding and given that the abstract does not require a high level of technical detail, we simplified the sentence as follows:

> "The results demonstrate an emergent pattern of concentration: increasing adoption rates of navigation services cause a reduction in the diversity of routes and a convergence of traffic and emissions on fewer roads."

Point 1.6

*I am unhappy with the first paragraph of the introduction: while this is all true, it has not much connection to your topic; I do not see that navigation services are a human-AI ecosystem. Human, yes, but AI? Nope, Dijkstra's algorithm is from the 1950ies, there is little AI here. Consider a **re-formulation**, or even removing. And please: this kind of interaction is also long known in the transport planning community, starting (but not ending) with the work of Wardrop (see below); more details can be found in Sheffi's book, which is a little outdated, but still a kind of reference in this area:*
https://sheffi.mit.edu/sites/sheffi.mit.edu/files/sheffi_urban_trans_networks_0.pdf

Also, please state that the Leonia and Brenner stories are in the form told here, are anecdotically. I am more than sure, that there are many more occurances like these, most likely with smaller effects so that they do not make it into the headlines. Nevertheless, it is truly difficult to actually fix and quantify this in real life. Furthermore, one has to weigh it against the positive features when those systems direct people to the fastest path which would otherwise run much longer pathes. And this happens during all non-peak times, which are typically more common than the peak times (but carry less traffic, sure).

Answer 1.6

We agree that the original opening paragraph was not sufficiently connected to the paper's main focus.

Following the Reviewer's suggestion, we removed Leonia and Brenner anecdotes and then revise the incipit of the paper as follows:

> "Navigation services (e.g., Google Maps and TomTom) recommend routes based on traffic conditions, guiding the movements of millions of drivers worldwide. While beneficial to individual drivers, algorithmic navigation has been linked to numerous documented cases of localised congestion. Yet, the trade-off between

> their benefits and detrimental effects on traffic dynamics remains poorly understood”.

Moreover, we thank the Reviewer for mentioning the interesting works of Sheffi. We now cite Sheffi’s book in the revised manuscript in the section “Simulation framework”.

Point 1.7

So far, your paper is a bit on the alarming side, which I do not like and which borders on journalistic. Consider a more neutral wording. And more of your wording is a bit too advertising ("unprecedented view" -- wow, "foundational step" for what is being done in the traffic engineers communities for ages without thinking too much about it since it is fairly trivial, see the remark about Sheffi above, and Wardrop below), please tone down a bit and stick to a neutral, scientific language.

Answer 1.7

We thank the Reviewer for this comment. In response, we have revised the manuscript throughout to adopt a more neutral and strictly scientific tone. We believe that, thanks to the Reviewer’s comment, the paper is now clearer and more rigorous.

Point 1.8

Once the penetration rate exceeds a service-specific threshold, these benefits diminish, disappear, or even reverse. >>

*This depends on the type of service actually offered, and I think for current navigation services it is true enough. However, and we have discussed this with colleagues from TomTom already 15 years ago, if the service does not simply offer a fastest path but in addition before doing so computes a (dynamic, stochastic) user equilibrium (see Sheffi, sorry for re-iterating), then such a service will lead to an **optimal utilization** of the network. Clearly, doing this is way too expensive for current navigation service providers, but in theoretical work as the one here, it should at least not go unmentioned.*

Answer 1.8

We thank the Reviewer for raising this important point. We discuss this point in the section “Simulation framework” when we discuss the algorithms behind navigation services. The revised paragraph is now as follows:

> “Although the algorithms underlying navigation services are opaque to us, we assume that they provide route recommendations optimised for individual requests rather than favouring system-level coordination. This is justified by their inability to enforce or forecast driver compliance, which makes system-optimal solutions based on Wardrop’s second principle unreachable.”

Point 1.9

For your OD matrix: I think it is critically important that it is time-dependent, i.e. m_{o,d}(t) or m_{o,d,t}. Have you done this? (Later on I have seen that you have not done it; but traffic does not depend only of peak hours, but of a continuum of demand between very small and very large.)

Answer 1.9

We thank the Reviewer for this comment, which allows us to clarify an important modelling choice. As the Reviewer correctly notes, we compute the OD matrix only for the selected morning peak hour, rather than as a fully time-dependent matrix.

We focus on the peak hour because it corresponds to the period of highest traffic load in the city and therefore to the conditions under which navigation services are most likely to generate non-trivial collective effects. When traffic demand is low – such as during off-peak hours or nighttime – traffic flows are sparse, congestion effects are limited, and recommending shortest or fastest routes to drivers is expected to be close to optimal and unlikely to produce adverse system-level outcomes.

By contrast, during peak hours, the road network operates near capacity, making routing interactions, congestion amplification, and route concentration effects particularly relevant. For this reason, concentrating on the peak hour provides a conservative and informative setting to study the potential collective impact of navigation services.

We explicitly acknowledge this choice at the end of the first paragraph of section "Impact of navigation services".

Point 1.10

Have you checked the overlap of SUMO's routes with the ones of the various providers?

Answer 1.10

Thank you for this comment. We had indeed computed this overlap but initially chose not to include it in the manuscript. We appreciate the Reviewer's suggestion and have now added this analysis.

The average Jaccard similarity between the routes suggested by navigation services and those computed by SUMO lies in the 52–63% range, while exact route matches account for 28–39%. This similarity is substantially lower than that observed among the navigation services themselves, whose mutual similarity ranges between 68% and 97%. This difference indicates that SUMO relies on different routing criteria and cost functions. We now report and briefly discuss these results in the section "Simulation Framework".

Point 1.11

"We assume that vehicles in the control group select their routes independently of one another" >>
But this is exactly what SUMO's duarouter is for. Which is essentially Wardrop's first principle. (https://en.wikipedia.org/wiki/John_Glen_Wardrop#Wardrop_equilibria)
And of course, Sheffi.

Answer 1.11

The Reviewer is right. To avoid confusion, we removed this sentence.

Point 1.12

"route diversity varies considerably with r following an exponential function" >>
I have not checked SM 7, *but 2a-c do not look exponential. Maybe you confused this with the \Delta E vs \Delta D? And: the change in route diversity is roughly a 10% effect, which I think is not that dramatic.*

Answer 1.12

We thank the Reviewer for raising this point and for prompting us to clarify both the functional form and the interpretation of the effect size. We confirm that the curves shown in Fig. 2a-c are best described by a function of the form $y = a e^{b x} + c$, which belongs to the exponential function family. While the visual appearance may not immediately suggest a steep exponential decay, formal model comparison (reported in Supplementary Note 5) shows that this functional form consistently provides the best fit across cities and traffic conditions when compared with linear, quadratic, and cubic alternatives.

Regarding changes in route diversity, although a 10% reduction may appear modest, its impact on traffic dynamics can be substantial. During peak hours, road networks operate close to capacity and exhibit strongly nonlinear behavior, so even small losses of routing diversity concentrate traffic on a limited set of corridors. At high adoption rates, these small reductions in route diversity have non-negligible effects on CO_2 emissions, as efficiency decreases and additional losses of diversity yield progressively smaller or even vanishing emission reduction benefits, as shown in Figure 4.

Point 1.13

"*Across all cities, we observe a consistent shift of routes, and consequently* CO_2 *emissions, toward high-capacity infrastructures such as highways and major arterial corridors (attractors).*" >>
As already mentioned: I cannot see anything bad here, since these roads are made for exactly this purpose. Should they route all this traffic through residential roads? What is your idea here? Furthermore, as is done in transport planning, one usually tries to estimate the immissions, and with this, target also how many people are actually affected.

For this, however, one must have additional information about the population, their vulnerability to the various immissions (CO2 and traffic load just one of them), which makes good transportation planning so complicated.

Answer 1.13

We agree with the Reviewer that directing traffic toward high-capacity corridors is not inherently negative and is consistent with their intended function in urban transport systems. In our analysis, we do not interpret this phenomenon as either beneficial or detrimental per se; rather, our aim is to characterize how navigation services reshape traffic dynamics and the spatial distribution of emissions at the system level. To avoid potential misunderstandings or misinterpretations of our results, we have clarified this point explicitly in the section “Impact of navigation services”.

Point 1.14

"Milan has the highest coefficient value (beta = 0.0068), indicating that changes in route diversity have a more significant impact on CO2 reduction compared to Florence (beta = 0.00313) and Rome (beta = 0.00226)" >> Do you have error measures and statistical significances for these parameters? Are the three beta different (I would assume this, but better safe than sorry)?

Answer 1.14

We thank the Reviewer for raising this point. We addressed it by testing statistical significance across the three values of beta using a joint nonlinear exponential model with city-specific beta parameters and using Wald. We find that the beta coefficient in Milan is significantly larger than in Rome ($p = 0.04$), whereas the coefficient estimated for Florence is not statistically distinguishable from either Milan or Rome. Moreover, a global likelihood-ratio test does not provide evidence for differences across all three cities simultaneously ($p = 0.22$).
In light of these results, we have removed the interpretation of beta as a comparative indicator across cities and eliminated all claims based on differences in its numerical value. The exponential fit is now used solely as a descriptive summary of the nonlinear, diminishing-returns relationship between marginal changes in route diversity and marginal changes in CO2 emissions.

Point 1.15

I am not exactly sure, why Fig. 4 is needed. It has nothing new over Fig. 3. Please discuss.

Answer 1.15

While Figures 2 and 3 separately show how navigation services affect route diversity and CO2 emissions as adoption increases, Figure 4 directly links changes in adoption rate to the joint variation of route diversity and CO2 emissions. This figure compactly summarizes how the relationship between route diversity and CO2 emissions depends

on the adoption level: at low adoption rates, small changes in route diversity are associated with substantial CO2 reductions, whereas at higher adoption rates, comparable reductions in route diversity yield progressively smaller emission benefits, revealing a clear diminishing-returns effect. We believe that Figure 4 captures this key mechanism in a single visualization and therefore merits retention.

Point 1.16

It might be picky, but I would prefer the correct writing of chemical formulas like CO2 *and* NOx *with indexed numbers.*

Answer 1.16

We have written correctly CO2 and NOx with indexed numbers as requested by the Reviewer.

Point 1.17

Fig. 3 caption: it says 3d-f are the Gini coefficients, but the y-axis description does not reflect this. Please adopt.

Answer 1.17

We thank the Reviewer for pointing this out. In revising the manuscript, Figure 3 has been reorganized, and the panels previously showing Lorenz curves and Gini coefficients have been moved to the Supplementary Material, where they are now reported consistently with the corresponding axis labels and captions. The caption and references have been updated accordingly to avoid ambiguity.

Point 1.18

Please fix Refs 26, 41, 49, *and maybe others which I overlooked. At least an* URL *or* DOI *would be very welcomed.*

Answer 1.18

Thank you for notifying us of this. We have corrected all references and added DOIs.

Reviewer #2

We thank the Reviewer for the helpful suggestions that improved the readability of the manuscript, and especially for the detailed comments that guided us in addressing the identified concerns.

Point 2.1

Indeed, the order of presentation in the manuscript is problematic and undermines its coherence and scientific communication quality. In high-impact journals the structure of a paper is not merely a stylistic matter but a reflection of logical flow, reproducibility, and argumentative strength.

Answer 2.1

We thank the Reviewer for pointing this out. We have revised the structure of the paper following the Reviewer's suggestions (see responses to the points below).

Point 2.2

Placement of the "Methods" section. Observation: The Methods section appears near the end of the manuscript, after the results and discussion. Although this format is acceptable in some journals, where methodological details are placed after the main text to emphasize narrative flow, it is not appropriate for technically oriented research, especially in computational simulation studies. Readers need to understand the experimental setup before interpreting the results. Suggestion:

1. *Abstract*
2. *Introduction*
3. *Methods*
4. *Results and Discussion*
5. *Conclusions*
6. *References*

This order preserves a natural progression from conceptual background to empirical validation and discussion.

Answer 2.2

We thank the Reviewer for this useful suggestion. The placement of the Methods section follows the editorial format of Nature Communications, which positions it at the end of the manuscript by default, and therefore cannot be altered. However, we have restructured the rest of the paper in line with the Reviewer's suggestions, and we agree that this has improved the clarity and presentation of our main messages.

Point 2.3

Absence of conclusions: *The manuscript lacks a conclusion section, which represents a significant structural flaw. Without it, the paper feels unfinished and lacks a synthesis of*

its theoretical, methodological, and practical contributions. Moreover, most Scopus- or WoS-indexed journals explicitly require a "Conclusions" section for indexing and thematic classification.

Answer 2.3

We have added a Conclusions section in which we discuss research directions suggested by our work. In particular, we explain why simulations are the only feasible approach for conducting traffic experiments, outline how our framework can be extended to investigate additional aspects, such as traffic safety and neighborhood-level segregation, as well as other mobility services, and connect our findings to the ongoing debate on AI co-evolution and human-AI feedback loops, with a specific focus on the impact of navigation systems in this context.

Point 2.4

Placement of figures and tables: *All figures and tables appear at the end of the manuscript. While this layout is acceptable for preprint submissions, it interrupts the visual continuity between text and evidence. Figures such as 1 (simulation flow) and 5 (concentration patterns) are key for understanding the methodology and should be embedded where first discussed. Integrate figures and tables within the main text or include reduced versions (thumbnails) in-line, referring to the full versions in the appendix. This will improve reader comprehension and facilitate peer review.*

Answer 2.4

Thank you for this suggestion. We have now positioned the figures at the points where they are first discussed, in order to facilitate the reading of the manuscript.

Point 2.5

The title of the manuscript, "Algorithmic navigation reduces route diversity and amplifies emissions inequality in cities," is appropriate in terms of scientific precision and conceptual clarity, as it accurately summarizes the study's main finding: the negative impact of algorithmic navigation on route diversity and the unequal distribution of urban emissions. However, from an editorial perspective, the title could be strengthened by explicitly referencing the methodological approach employed (simulation based on real-world data) and the type of emissions analyzed (CO_2), which would broaden its scope and enhance the identification of its application field. Suggestions such as "Modeling the Urban Impact of Algorithmic Navigation: Reduced Route Diversity and Unequal CO_2 Emissions" or "Algorithmic Navigation Concentrates Urban Traffic and CO_2 Emissions, Reducing Route Diversity in Cities" would add greater specificity and appeal for interdisciplinary audiences in mobility, artificial intelligence, and urban sustainability, without losing the scientific rigor of the original title.

Answer 2.5

Thank you for this useful suggestion. We found it challenging to identify a title that was both concise and accurately conveyed the message of the paper. Following the Reviewer's suggestion, and after a discussion and other colleagues, we propose the following revised title, which combines the second title suggested by the Reviewer with the title used in the previous version of the manuscript: "Navigation services concentrate traffic and emissions in urban spaces"

Point 2.6

Abstract. *The abstract of the article presents a coherent and academically solid structure, introducing the study's context, methodology, results, and implications. However, several aspects could be improved to meet the scientific writing standards expected in journals, particularly regarding methodological precision, quantification of results, and connection with practical and regulatory implications. Below are the specific observations and suggestions organized by content blocks.*

Point 2.6.1 ***Lines 1–5***: *The opening effectively presents the study's context, but the introductory sentence is too general and does not immediately define the central scientific problem.*

Suggestion: *Rephrase the first couple of sentences to establish from the beginning the relationship between algorithmic navigation and its environmental impact, for example: "Algorithmic navigation systems, widely used in urban mobility, can unintentionally reshape traffic flows and amplify emission inequalities."*

Point 2.6.2 ***Lines 6–12***: *The abstract mentions that the study employs a simulation-based framework using real-world data, but it does not specify the nature or origin of the data.*

Suggestion: *Include a brief reference to the source or type of data used (e.g., GPS data, urban traffic records, or mobility datasets), which would enhance transparency and reproducibility.*

Point 2.6.3 ***Lines 13–18***: *The results are presented generally, indicating that navigation services reduce emissions at low adoption rates but increase them at higher ones. However, no quantitative effects are provided.*

Suggestion: *Add approximate values or variation ranges (e.g., percentage reduction or increase in* CO_2*) to strengthen the empirical rigor and impact of the summary.*

Point 2.6.4 ***Lines 19–24***: *The description of the nonlinear function relating route diversity and emissions is interesting but introduced abruptly, without a clear connection to the rest of the text.*

Suggestion: *Include a short transition phrase to contextualize its relevance, for example: "To mathematically capture this dynamic, we describe the relationship between route diversity and* CO_2 *emissions through an exponential function."*

Point 2.6.5 ***Lines 25–30:*** *The closing paragraph mentions the applicability of the proposed framework and the regulatory relevance of the study but does not elaborate on its broader implications or specific policies it may inform.*

__Suggestion:__ Add a sentence highlighting the implications for urban planning or digital platform regulation, for example: "These findings may inform urban policy and digital platform governance, especially under frameworks such as the EU Digital Services Act."

Point 2.6.6 ***Lines 31–33:*** *The abstract ends abruptly, lacking a final sentence that reinforces the scientific contribution or added value of the work.*

__Suggestion:__ Conclude with a statement emphasizing the novelty and interdisciplinary relevance of the study, for example: "This work offers a novel perspective on how AI-driven mobility systems can shape environmental inequalities in cities."

Answer 2.6

We thank the Reviewer for helping us improve the abstract. We have improved the manuscript as follows:

Answer 2.6.1 Also considering Point 1.4 by Reviewer one, we rephrased the incipit of the abstract as follows:

- "The collective impact of navigation services is unclear: while often beneficial to individual drivers, they can also unintentionally reshape traffic patterns in urban spaces".

Answer 2.6.2 We modified the second sentence of the abstract to clarify the nature and origin of data used for the simulations:

- "Our study employs a simulation-based framework for evaluating the impact of navigation services across three cities, integrating GPS data, road networks, and route recommendations offered by leading providers."

Answer 2.6.3 We revised the part in the abstract regarding the results as follows:

- "The results demonstrate an emergent pattern of concentration: increasing adoption rates of navigation services cause a reduction in the diversity of routes and a convergence of traffic and emissions on fewer roads. At full adoption of the service, the diversity of routes is reduced across all cities, with reductions up to 14% compared to a baseline scenario where route recommendations are not followed at all."

Answer 2.6.4 We modified the sentence regarding the non-linear function as suggested by the Reviewer:

- "We show that the relationship between route diversity and CO_2 emissions is mathematically described by an exponential function."

Answer 2.6.5 and Answer 2.6.6 We have revised the closing paragraph as follows:

- “Our study is relevant for urban planning and digital platform governance, shedding light on how AI-driven mobility systems can reshape traffic and emissions in urban spaces.”

Point 2.7

Introduction. *The introduction of the article progressively presents the context of human–AI ecosystems and their side effects, establishing a parallel between different types of algorithms—such as recommendation systems or digital navigation—and their social impacts. It then focuses on commercial navigation services, providing real-world examples of urban congestion caused by the widespread use of these platforms. The authors subsequently identify the existing scientific gap regarding a comprehensive analysis of their influence on traffic and emissions and describe the methodological framework proposed to address this issue. Finally, the introduction anticipates the main results and the regulatory relevance of the study, linking them to policies such as the European Union's Digital Services Act.*

Point 2.7.1 ***Lines 19–28****: The transition between the examples of human–AI interaction and the topic of vehicular navigation is not entirely clear.*

Suggestion*: Add a linking sentence that explicitly connects the effects of recommendation systems with those of algorithmic navigation, for example: “A similar pattern may emerge in algorithmic systems shaping urban mobility.”*

Lines 29–40*: The narrative tone of the Leonia and Italian region cases is more journalistic than academic.*

Suggestion*: Rephrase the descriptions in a scientific style, avoiding colloquial expressions, for example: “residents experienced severe accessibility restrictions due to algorithm-induced congestion.”*

Point 2.7.2 ***Lines 41–57****: The research question is expressed rhetorically, and the paragraph contains overly long sentences.*

Suggestion*: Convert the question into a declarative statement and split long sentences for better readability, for example: “This study aims to quantify the impact of navigation service adoption rates on urban traffic and* CO_2 *emissions.”*

Point 2.7.3 ***Lines 58–65:*** *There is a lack of contextual information regarding the cities or datasets used in the simulation framework.*

Suggestion*: Include a brief mention of the data source or characteristics (scale, region, mobility pattern) to enhance reproducibility.*

Point 2.7.4 ***Lines 66–84:*** *This section mixes results and analysis that should be reserved for later sections.*

Suggestion*: Summarize the main ideas and move the detailed discussion of non-linear behavior and the exponential function to the results section.*

Point 2.7.5 ***Lines 85–91****: The conclusion limits the study's applicability to the European context.*

Suggestion*: Add a brief statement on the potential applicability of the methodological framework to other regions to broaden the study's global scope.*

Answer 2.7

Thanks for these suggestions, which helped us improve the introduction of the paper. We have improved it as follows:

- Answer 2.7.1 Also considering Point 1.6 raised by Reviewer 1, we have removed the initial discussion on human–AI ecosystems and refocused the introduction to start directly with navigation services. Moreover, we removed the explicit mentions to anecdotes and embedded them into a broader discussion about documented cases of localized congestion. The revised opening paragraph of the manuscript is now as follows:
  - "Navigation services (e.g., Google Maps and TomTom) recommend routes based on traffic conditions, guiding the movements of millions of drivers worldwide. While beneficial to individual drivers, algorithmic navigation has been linked to numerous documented cases of localised congestion. Yet, the trade-off between their benefits and detrimental effects on traffic dynamics remains poorly understood."

- Answer 2.7.2 We have changed the end of the second paragraph in the introduction as follows:
  - "This study fills this gap by quantifying the impact of various navigation services on road network usage and CO2 emissions in three cities of different sizes."

- Answer 2.7.3 We have added the requested information to the paragraph, which now reads as follows:
  - "We design a simulation framework that reproduces urban traffic dynamics by integrating mobility demand information from GPS data, road networks from OpenStreetMap, and route recommendations from several popular navigation services: Bing Maps, Google Maps, Mapbox, and TomTom. We use this framework to assess the impact of these navigation services in Florence, Milan, and Rome (Italy) by simulating different levels of service adoption. For each adoption rate, vehicles are randomly assigned to treatment and control groups, with the treatment group following algorithmic route recommendations. This setup enables a causal assessment of how adoption rates of navigation services determine changes in traffic dynamics.."

- Answer 2.7.4 Our intention was to summarize the main findings of the paper in the Introduction, in order to provide readers with a concise overview of what they would encounter in the subsequent sections. We agree, however, that this summary can be streamlined and that some analytical aspects are better

deferred to the Results section. We have therefore revised the paragraph accordingly, as follows:

- "We find that route recommendations substantially reshape road network usage and CO_2 emissions. Across all cities and navigation services, higher adoption rates cause a reduction in route diversity, i.e., recommended routes increasingly concentrate on a smaller set of roads. Under light traffic loads, navigation services reduce CO_2 emissions. Under high traffic loads, navigation services reduce emissions at low adoption levels; however, these benefits weaken, vanish, or reverse as adoption increases. To assess the generalisability of these findings, we replicate our experiments using an abstract representation of road networks and traffic dynamics, finding solid agreement with the patterns observed in real-world cities."

Answer 2.7.5 Thank you for this suggestion. We have modified the final paragraph of the Introduction as follows:

- "Our study sheds light on the multifaceted impact of algorithmic navigation across multiple cities and services, and it is replicable worldwide, given the widespread availability of mobility demand and road network data. Ultimately, our framework supports urban decision-makers in assessing the impacts of navigation services and designing targeted policy interventions. "

Point 2.8

Simulation Framework. *The "Simulation Framework" section provides a detailed methodological structure used to model the impact of navigation services on urban traffic and emissions. The text follows a logical sequence: it begins by defining the simulation environment (SUMO), explains the representation of the road network and the generation of mobility demand, details the integration with commercial navigation service APIs, and concludes with the experimental design, evaluated metrics, and methodological limitations.*

Point 2.8.1 ***Lines 93–98***: *The introduction of the SUMO tool and the justification for its use are clear, but there is no brief reference to its validation or accuracy in previous studies.*

Suggestion: *Add a sentence supporting the choice of SUMO by citing recent literature on its reliability as a traffic simulation tool, for example: "SUMO has been validated in several large-scale traffic studies for its accuracy in replicating real-world urban conditions (Behrisch et al., 2019)."*

Point 2.8.2 ***Lines 99–115***: *The explanation of the construction of the road graph and OD matrix is precise but somewhat lengthy and too technical for a main section.*

Suggestion: *Condense the mathematical description of the process and refer readers to the Methods or Supplementary Material for details. Additionally, briefly*

explain why "square tiles" were chosen instead of other spatial divisions (e.g., census zones or districts) to justify spatial representativeness.

Point 2.8.3 ***Lines 116–126***: *The description of API-based navigation services is thorough, but the justification for forcing the use of historical information (instead of real-time traffic data) could be more explicit regarding experimental validity.*

Suggestion: *Add a brief methodological rationale explaining how this choice avoids bias or ensures comparability, for example: "Using historical traffic conditions ensures experimental consistency and eliminates noise from exogenous real-time events."*

Point 2.8.4 ***Lines 127–135***: *The navigation services used are listed, but the selection criteria are not explained, nor is there clarification on how differences among algorithms (e.g., optimization heuristics) are handled.*

Suggestion: *Include a sentence justifying the selection of these services (due to commercial relevance or API accessibility) and acknowledge the "black-box" limitation of the algorithms, emphasizing how this may influence result variability.*

Point 2.8.5 ***Lines 136–149***: *The experimental design with treatment and control groups is well described, but the modification of the driving model (to simulate human imperfections) is too briefly discussed.*

Suggestion: *Expand slightly on the explanation of the "fastest route" modification and its basis in driver behavior literature, or move these details to the Methods section for greater clarity.*

Point 2.8.6 ***Lines 150–157***: *The choice of metrics (route diversity and CO_2 emissions) is appropriate but lacks justification for excluding other relevant variables such as energy consumption or average travel time.*

Suggestion: *Add a brief justification for focusing on these metrics, emphasizing their environmental relevance and connection to the study's central hypothesis.*

Point 2.8.7 ***Lines 158–167***: *The limitations section correctly addresses the issue of group interference, but the reference to the Stable Unit Treatment Value Assumption (SUTVA) may be too technical for non-specialist readers.*

Suggestion: *Complement this with a more accessible explanation of the practical implications, for example: "Because vehicles influence each other's movements, the separation between treatment and control groups is imperfect, potentially diluting observable effects." It would also be useful to include a note on the expected magnitude of this bias or any statistical controls used.*

Answer 2.8

Answer 2.8.1 We agree that the Introduction should briefly justify SUMO also in terms of empirical reliability. We have added the sentence suggested by the Reviewer noting that SUMO has been validated against real-world urban measurements in multiple large-scale city scenarios. We also cite the standard SUMO overview paper for background on the tool and its established use in the traffic-simulation community. The paragraph is now as follows:

- "Our simulation framework leverages SUMO, an open-source, agent-based traffic simulator developed by the German Aerospace Center and used in several large-scale studies for its realism in replicating traffic dynamics."

Answer 2.8.2 We have condensed the explanation of the road network and OD-matrix construction, removing technical and mathematical specifics and referring readers to the Methods and Supplementary Material for full procedural details. In addition, we now explicitly justify the choice of equally sized square tiles as the spatial discretization scheme. We clarify that square tiles provide a uniform and administration-independent spatial representation, avoiding the heterogeneity and potential biases associated with census zones or administrative districts, which vary widely in size and functional meaning. This choice ensures consistent spatial resolution and facilitates comparability across cities. The paragraph is now as follows:

- "Square tiles provide a spatially uniform discretisation, avoiding biases introduced by administrative boundaries (e.g., census zones or districts), which may vary in size, shape, and functional meaning. This choice ensures consistent spatial resolution across the city and facilitates comparability across urban areas."

Answer 2.8.3 We have revised the paragraph to clearly explain that forcing the use of historical (typical) traffic information ensures experimental validity, consistency, and comparability across simulated scenarios. In particular, this choice eliminates noise and potential biases introduced by unpredictable exogenous real-time events (e.g., accidents, strikes, or road works) that are not explicitly modeled in the simulation framework. As a result, the generated routes are coherent with the simulated traffic dynamics and reflect the navigation service's recommendations under representative urban conditions. The paragraph is now as follows:

- "We query routes for future dates relative to the request time, thereby forcing the APIs to rely on typical traffic information rather than real-time conditions. This choice avoids incorporating signals shaped by exogenous and unpredictable events - such as accidents, strikes, or roadworks - that cannot be modelled within the simulation. "

Answer 2.8.4 We have revised the manuscript to explicitly justify the selection of the navigation services considered in section "Simulation framework" as follows:

- "We consider a set of navigation services that provide public APIs: Bing Maps, Google Maps, Mapbox, TomTom Fastest, TomTom Shortest, and TomTom Eco-routing. At the time of the experiments, Apple Maps, Waze, and Google Maps eco-routing could not be included due to the unavailability of public APIs. These services were selected because of their

commercial relevance and widespread adoption, which makes them suitable for large-scale, reproducible experiments"

Answer 2.8.5 We have slightly expanded the explanation in the main text to clarify its behavioral basis, while moving the technical details to the Methods section. This revision makes explicit that the modification is intended to capture well-documented human imperfections in route choice, rather than to introduce an alternative optimization strategy.

Answer 2.8.6 We focus on route diversity and CO2 emissions because they directly capture the core mechanism and hypothesis of our study: that navigation services can induce traffic concentration by reducing route diversity, with downstream environmental consequences. Route diversity quantifies how traffic is spatially distributed across the network, while CO2 emissions provide an integrated, policy-relevant measure of environmental impact that reflects congestion, stop-and-go dynamics, and vehicle interactions. We have clarified this rationale in the revised manuscript.

Answer 2.8.7 We have complemented the discussion with a more intuitive explanation of the practical implications of vehicle interactions, clarifying why the separation between treatment and control groups is imperfect as follows:

- "A limitation of our experimental setup arises from interaction effects: since vehicles move on a shared road network, those assigned to the control group inevitably interact with vehicles in the treatment group. As a result, the outcomes of individual vehicles are not fully independent and observable differences between groups are expected to be underestimated. This is a common situation when performing controlled experiments in complex social systems. Our results should therefore be interpreted as conservative estimates of the impact induced by navigation services."

Point 2.9

Impact of Navigation Services. *This section presents the experimental results and analyzes how the progressive adoption of algorithmic navigation services affects route diversity and* CO_2 *emissions in three Italian cities. The structure is clear and logically sequenced: it begins with the selection of cities, then discusses results on route diversity, total emissions, the spatial distribution of emissions, optimal adoption patterns, and finally the quantitative relationship between route diversity and emissions.*

Point 2.9.1 ***Lines 169–175****: The selection of cities is well justified by their heterogeneity, but the explanation is more descriptive than analytical.*

Suggestion*: Add a brief scientific justification explaining how morphological and structural differences among the cities help validate the generalizability of the*

results, for example: "These cities represent diverse topologies that enable assessing the robustness of algorithmic navigation effects under different urban morphologies."

Point 2.9.2 ***Lines 176–190***: *The description of results regarding route diversity is clear, but the text includes too many numerical values that disrupt the flow of reading.*

Suggestion: *Retain only the most representative figures and move secondary percentages to the supplementary material. Also, summarize the overall behavior in an interpretive sentence, for example: "Route diversity initially increases at low adoption levels but sharply declines once the adoption threshold is surpassed."*

Point 2.9.3 ***Lines 191–197:*** *The interpretation of traffic concentration is correctly framed but somewhat repetitive compared with the methodology section.*

Suggestion: *Restructure the paragraph to emphasize the causal mechanism (algorithmic route homogenization) and its link to the OD matrix structure, avoiding repetition of previously discussed explanations.*

Point 2.9.4 ***Lines 198–204***: *The emissions results are presented precisely, but the narrative lacks comparative insight across cities and navigation services. Suggestion: Introduce a short comparative analysis summarizing key differences, for example: "The emission-reduction patterns are more pronounced in smaller cities like Florence and less consistent in larger urban areas such as Rome."*

Point 2.9.5 ***Lines 205–221***: *The discussion on emission inequality is compelling, but it overuses visual descriptions (figure references) without interpreting their urban or environmental implications.*

Suggestion: *Complement the analysis with a short interpretation of the environmental and social meaning of this concentration, for example: "This spatial concentration of pollutants may exacerbate localized air quality degradation and social exposure disparities."*

Point 2.9.6 ***Lines 222–232***: *The analysis of "attractors" is relevant, but the term may be ambiguous for readers from other disciplines.*

Suggestion: *Explicitly define what is meant by "attractors" (e.g., "high-capacity road segments that algorithmically attract flows") and add a reflection on the conflict between algorithmic efficiency and environmental sustainability.*

Point 2.9.7 ***Lines 233–243***: *The text includes numerous numerical values and adoption thresholds but lacks a synthesis of general implications.*

Suggestion: *Add a closing statement summarizing the key finding, for example: "These results indicate that there exists an optimal adoption range—typically between 60% and 80%—beyond which the environmental benefits of navigation services decline."*

Point 2.9.8 ***Lines 244–247***: *The argument regarding the limitations of eco-routing is conceptually strong but phrased too simplistically.*

Suggestion: *Reinforce the theoretical idea by stating that sustainability depends not on individual routes but on the collective distribution of algorithmic decisions, which broadens the relevance of the finding.*

Point 2.9.9 ***Lines 248–269***: *The description of the correlation between route diversity (ΔDr) and emissions (ΔEr) is solid but overly technical and dense with formulas.*

Suggestion: *Simplify the mathematical explanation by emphasizing the conceptual interpretation: "A strong negative correlation between route diversity and CO_2 emissions indicates that as traffic becomes more concentrated, marginal emission reductions diminish." Also, move the formal equation to the Methods section or Supplementary Material.*

Point 2.9.10 ***Lines 270–278***: *The section opens with a clear rationale, emphasizing the need to isolate the fundamental mechanisms of traffic concentration. However, the explanation of the experimental simplification is overly brief.*

Suggestion: *Expand on why a grid-based network was chosen as a simplification, for example: "The grid structure allows controlling network topology while preserving key flow interaction properties." This would strengthen the methodological justification.*

Point 2.9.11 ***Lines 279–284***: *The description of the first experiment (single OD flow) is precise but lacks interpretive depth. The authors mention that route diversity decreases while emissions also decline but do not analyze why.*

Suggestion: *Include a short interpretation linking this to the absence of vehicle interactions or congestion, such as: "This suggests that in the absence of network interactions, concentration effects remain benign and do not generate emission inefficiencies."*

Point 2.9.12 ***Lines 285–292***: *The second experiment (two intersecting OD flows) effectively reproduces real-world congestion effects, yet the text relies heavily on figure references instead of analytical commentary.*

Suggestion: *Add a more conceptual discussion highlighting how the intersection of flows introduces systemic interdependence, transforming a purely geometrical setup into a dynamic analogue of urban congestion.*

Point 2.9.13 ***Lines 293–296***: *The robustness verification through larger and randomized networks is appropriate but superficially discussed.*

Suggestion: *Briefly mention how network regularity and randomness affect the persistence of the observed effects to show that the findings are not limited to grid symmetry. For instance: "This confirms that the emergence of concentration patterns is a structural property of algorithmic routing, not a by-product of network regularity."*

Point 2.9.14 ***Lines 297–299***: *The concluding statement effectively generalizes the findings but could be more assertive regarding their implications.*

Suggestion: *Reinforce the conclusion by linking it to the broader debate on algorithmic urbanism, e.g., "These results indicate that algorithm-driven concentration is a universal phenomenon likely to manifest across diverse urban morphologies, highlighting the need for regulatory attention."*

Answer 2.9

Answer 2.9.1 We have revised the paragraph as follows:

- "We conduct experiments in three cities in Italy: Florence, Milan, and Rome. These cities were chosen due to their heterogeneity in size, population, and road network structure. [...] The difference among cities enables assessing the robustness of algorithmic navigation effects across different urban morphologies."

Answer 2.9.2 While Reviewer's suggestion is reasonable, moving all numbers to the Supplementary Material is in contrast with the suggestion of Reviewer #1 to limit as much as possible cross-references to Supplementary Material as well as the number of Supplementary Notes. Moreover, we think that providing numerical detail may be useful to assess the impact of navigation services. Therefore, we decided to retain this information in the paper.

Answer 2.9.3 We agree that this interpretation was partially repetitive. We therefore moved the interpretative discussion from the Results to the "Discussion" section avoiding overlap with the methodology part.

Answer 2.9.4 Thank you for this suggestion. We investigated whether emission-reduction patterns systematically varied with city size but did not observe a consistent trend. Although Rome (the largest city) shows the lowest reduction, Milan (the second-largest city) exhibits higher reductions than Florence (the smallest city). Given this lack of a clear size effect, we opted to present and compare the percentage reductions for each city individually and revised the paragraph accordingly to improve cross-city comparison. We also refer to Table S2 for a more detailed numerical comparison across cities and navigation services.

Answer 2.9.5 Thank you for this suggestion. To comply with your general comment to keep the Results section lighter, we relocated this interpretative discussion to the Discussion section, where we now explicitly address the environmental and social implications of emission concentration, including potential exacerbation of localized air quality degradation and social exposure inequalities.

Answer 2.9.6 We agree that the term "attractors" may be ambiguous for readers from different disciplines. We have removed this term and revised the paragraph accordingly. We refer to this type of road infrastructure as "high-capacity roads".

Answer 2.9.7 Thank you for the suggestion. We added a paragraph summarizing the key finding.

Answer 2.9.8 We have revised the paragraph to emphasize that environmental sustainability emerges from the collective distribution of algorithmic routing decisions rather than from the optimality of individual routes. This broader framing highlights why eco-routing alone may be insufficient to control system-level impacts. The paragraph has been revised accordingly.

Answer 2.9.9 We have removed the explicit formula and now refer the reader to Figure 4a–c for details. The paragraph has been revised accordingly to reduce technicality and improve conceptual clarity.

Answer 2.9.10 Thank you, we agree. We added the following sentence:

- "This grid structure enables precise control of network topology while preserving essential flow interaction properties".

Answer 2.9.11 Thank you for the suggestion. We rephrased it as:

- "This suggests that, in the absence of vehicle interactions at intersections, reduced route diversity alone does not necessarily lead to a diminishing return effect."

Answer 2.9.12 We have added the following sentence:

- "In this configuration, the fastest routes intersect at the centre of the grid, becoming interdependent and producing collective concentration effects."

Answer 2.9.13 We have incorporated this sentence in the manuscript:

- "This confirms that the observed concentration patterns are not artefacts of network layouts."

Answer 2.9.14 We thank the Reviewer for this suggestion. We agree that the implications of our findings for the broader debate on algorithmic urbanism deserve explicit emphasis. We therefore chose to address this point in the Discussion section, where we added a paragraph situating our results within the context of algorithm-driven urban concentration and its relevance for regulation and policy.

Point 2.10

Discussion. This section effectively connects the empirical findings with their theoretical, social, and policy implications. The narrative is well-structured, moving from the summary of results to reflections on urban inequality, the extension of the model to other mobility platforms, and finally to the human–AI interaction and regulatory implications. However, while the content is solid, the writing could benefit

from stronger conceptual integration and deeper argumentation on the normative and ethical implications of algorithmic mobility systems.

Point 2.10.1 **Lines 301–305**: The opening paragraph summarizes the main finding (traffic and emissions concentration) clearly but does not explain how this concentration emerges as a systemic phenomenon beyond the observed empirical patterns.

Suggestion: Add a sentence that frames the phenomenon as an emergent property of socio-technical systems, for example: "This pattern can be interpreted as an emergent property of algorithmic coordination within decentralized mobility systems."

Point 2.10.2 **Lines 307–315**: The discussion on urban inequality is pertinent, but it focuses mainly on material consequences (congestion, pollution, property values) without addressing deeper notions of environmental or urban justice.

Suggestion: Strengthen this argument by connecting it with the theoretical framework of environmental justice (Harvey, 1996; Schlosberg, 2007), emphasizing that traffic concentration not only produces economic dysfunction but also environmental exclusion and unequal exposure to risk.

Point 2.10.3 **Lines 316–322**: Extending the model to shared mobility platforms is a conceptual strength, but the text does not clearly distinguish between algorithmic recommendation mechanisms and those of matching or dispatching (as in ride-hailing).

Suggestion: Clarify how the framework can adapt to different algorithmic decision models (e.g., dynamic matching vs. deterministic routing) and how these distinctions influence the simulation of environmental impacts.

Point 2.10.4 **Lines 323–330**: The discussion on human–AI coevolution is theoretically relevant but remains somewhat superficial: the "feedback loop" is mentioned without exploring its temporal dimension or implications for adaptive algorithmic learning.

Suggestion: Reinforce this point by referencing theories of algorithmic feedback loops (Rahwan et al., 2019; Epstein et al., 2020) or adaptive socio-technical systems, explaining how algorithmic recommendations create behavioral dependencies that perpetuate congestion patterns.

Point 2.10.5 **Lines 331–342**: The closing discussion on replicability and public policy is strong but written in a mostly descriptive tone. It lacks a critical reflection on how existing legislation (Digital Services Act and Digital Markets Act) could materially affect the transparency and governance of navigation algorithms.

Suggestion: Add a statement discussing the limitations of current regulation, for example: "However, transparency mandates alone may not suffice without enforceable accountability mechanisms to ensure that algorithmic behavior aligns with public policy objectives."

Answer 2.10

Answer 2.10.1 Thank you, we added this sentence at the end of the first paragraph:

- “The spatial concentration observed in our study emerges as a collective property of algorithmic route recommendations. Based on our results, we argue that this effect stems from the tendency of navigation services to suggest similar routes in response to similar requests. Optimal routes are typically funnelled through the same efficient roads; as a consequence, many optimal routes overlap on a limited set of road edges, producing traffic concentration on such edges.”

Answer 2.10.2 We have developed two paragraphs, one discussing urban inequality and the other focusing on environmental sustainability.

Answer 2.10.3 Thank you, we have explained what are the matching and dispatching algorithms behind ride-hailing or car-sharing services, and we have added a more detailed description on how our framework can be extended to simulate the impact of such algorithms. We added this in the discussion section.

Answer 2.10.4 Thanks for the suggestion. We have expanded the discussion of feedback loops in the conclusion section, by introducing an example from a closely related context (location-based recommender systems).

Answer 2.10.5 Thank you, we have further developed the discussion about how existing legislation could affect governance of navigation services.

Point 2.11

Methods.

Point 2.11.1 **Lines 344–354**: Road Networks. The description of the road network modeled as a directed weighted multigraph is accurate, but the mathematical formalism may be excessive for readers of interdisciplinary journals. Additionally, the validation using Google Maps and StreetView is mentioned, but the extent of the corrections is not quantified.

Suggestion: Simplify the formal notation, relegating mathematical details to the supplementary materials. Including an estimate of the percentage of manually corrected edges (e.g., “approximately 3–5% of edges required manual adjustments”) would enhance methodological transparency.

Point 2.11.2 **Lines 355–367**: The description of SUMO and its use of the HBEFA3 emission model is precise, but the emission equation lacks context regarding units and coefficient interpretation.

Suggestion: Add a brief note explaining how the parameters c0,...,c5c_0, \ldots, c_5c0,...,c5 correspond to different vehicle types (e.g., diesel vs. gasoline) and

how these distinctions are integrated into the model. This would strengthen the connection between simulation output and real-world emission behavior.

Point 2.11.3 **Lines 368–384**: The selection of navigation services is well justified in terms of availability and diversity, but the comparison methodology among them could be more clearly explained.

Suggestion: Specify whether route requests were made under identical temporal and geographic conditions for all services and how discrepancies in output formats were handled. It would also be advisable to briefly discuss the potential accessibility bias introduced by using only public APIs (since services such as Waze or Apple Maps were excluded).

Point 2.11.4 **Lines 385–401**: The introduction of the randomness parameter w in the duarouter algorithm is an interesting methodological contribution, but the explanation might be difficult for non-specialist readers.

Suggestion: Include a short practical interpretation, such as: "This parameter simulates the stochastic nature of human decision-making in routing, such as preferences for familiar paths or avoidance of complex intersections." Also clarify whether the chosen value $w = 5$ was based on empirical calibration or adopted from previous studies.

Point 2.11.5 **Lines 402–438**: The description of the GPS dataset is comprehensive and one of the study's major strengths. However, this section could benefit from a more critical discussion about representativeness limitations (only 2% market penetration).

Suggestion: Add a methodological reflection on sampling bias and explain how it was mitigated (e.g., through OD matrix weighting or validation against traffic counts). It would also be valuable to justify the choice of Wednesday as a reference day, supported by a methodological citation from transportation research.

Answer 2.11

Answer 2.11.1 We agree that the mathematical formalization of the road network was unnecessary for the scope of the main text. Accordingly, we have simplified the description in the Methods section and removed the formal notation. We have also clarified the extent of the manual validation process. Corrections based on Google Maps and Street View were minimal, involving fewer than approximately 0.15% of the edges across all road networks. We now explicitly report this value in the manuscript to improve methodological transparency.

Answer 2.11.2 Following Reviewer 1's suggestion, we removed unnecessary implementation details related to SUMO and retained in the main text only the fundamental concepts required to understand its role in our framework. In addition, we have added the "Impact measures" subsection of the Methods to include a more detailed description of the emission model parameters. In the "impact measures" we further specify that the coefficients used in the emission

equation correspond ta “a gasoline powered Euro norm 4 passenger car modeled using the HBEFA3 based model” as specified in the SUMO documentation (https://sumo.dlr.de/docs/Models/Emissions.html).

Answer 2.11.3 Following Reviewer 1’s suggestion, we removed the Methods section on navigation services and integrated the relevant material into the main text. This section has been expanded to clarify that route requests were submitted under identical conditions for all services. No significant discrepancies in output formats were observed that would require reporting. We also explicitly note that APIs for some navigation services were unavailable at the time of the simulations.

Answer 2.11.4 We have added a practical interpretation of the parameter w. We also clarified that we tested w={3,5,7}, and observed no meaningful changes in the results. Accordingly, we selected w=5 (the middle value) as a representative value.

Answer 2.11.5 The GPS data used in this study have an estimated coverage of 2–5%, depending on the geographic area. We provide a more detailed description of the dataset in the Methods, citing prior studies that have extensively used this data and shown it to be representative of private-vehicle mobility patterns. We consider all trips made during morning peak hours on Wednesdays over a full year. Flows were aggregated by summing trips between OD pairs across all Wednesdays, after which infrequent flows were removed. Focusing on Wednesdays allows us to capture representative weekday traffic while avoiding anomalies associated with weekends or atypical travel days. These details are described in the GPS Data subsection of the Methods and in the Supplementary Note 1.

The concentration effects of urban navigation services

Summary of changes for revision

We thank the Referees for their insightful comments and for the overall appreciation of our manuscript. We are particularly pleased that Reviewer #1 considers that all previous concerns have been fully addressed and supports the publication of the paper. We are also grateful to Reviewer #2 for the careful evaluation of the revised manuscript and for acknowledging the improvements in clarity, methodological transparency, and overall structure.

In the revised version of the manuscript, we have addressed the remaining concern raised by Reviewer #2 and revised the manuscript accordingly. All new or modified parts with respect to the previous version are highlighted in blue in the manuscript. We have also slightly changed the title to make it more effective.

We believe that these additional revisions further strengthen the contribution of our work and improve the clarity and generalizability of our findings, making the manuscript suitable for publication in *Nature Communications*.

Kind regards,
Luca Pappalardo, on behalf of all the authors

Reviewer #1

Reviewer's comment: “This is the revised review: the authors have paid heed to all of my points, therefore the paper is fine with me and can be published.”

We sincerely thank the Reviewer for the positive evaluation of the revised manuscript and for confirming that all previous concerns have been adequately addressed. We appreciate the Reviewer’s support for the publication of our work. We also believe that the Reviewer’s comments have contributed to significantly improving the quality and clarity of the manuscript.

Reviewer #2

We thank the Reviewer for the careful reassessment of our manuscript and for the positive feedback regarding the improvements in clarity, methodology, and overall presentation.

Point 2.1

While the authors have attempted to address the comment regarding the comparison between cities, the discussion remains relatively limited and largely descriptive. Given that the study includes multiple cities with potentially different urban morphologies and traffic characteristics, a slightly deeper comparative discussion would strengthen the contribution of the paper. Expanding this section to better highlight how and why the observed effects differ (or remain consistent) across the cities analyzed would improve the generalizability and interpretation of the results. I therefore recommend that the authors further refine and expand the inter-city comparison in the results or discussion section. Aside from this point, the manuscript has satisfactorily addressed the reviewer’s previous comments

Answer 2.1

We thank the Reviewer for this comment. We have included this discussion in the section "Impact of navigation services" after presenting the results regarding route diversity and CO_2 emission. The revised paragraph is as follows:

> "A comparison of Florence, Milan, and Rome reveals both common and city-specific patterns: route concentration is a consistent outcome of increased service adoption, while CO_2 emissions vary with the spatial redistribution of traffic induced by navigation services. In Florence, navigation services shift traffic toward an arterial road that is moderately used in the no-adoption scenario (Figure 3a-b). This redistribution is associated with a substantial reduction in CO_2 emissions that stabilises as this corridor becomes saturated at high adoption levels. In Rome, traffic is redirected onto a few major roads that are already heavily used in the baseline scenario (Figure 3g-h). This pattern may explain the pronounced increase in CO_2 emissions at high adoption rates. Milan lies between these two cases, navigation services concentrate traffic onto several major roads that are lightly trafficked in the baseline scenario (Figure 3d-e), which is consistent with the moderate decreases in CO_2 emissions observed at high adoption rates."
